# Loss of Ambra1 promotes melanoma growth and invasion

**DOI:** 10.1038/s41467-021-22772-2

**Published:** 2021-05-05

**Authors:** Luca Di Leo, Valérie Bodemeyer, Francesca M. Bosisio, Giuseppina Claps, Marco Carretta, Salvatore Rizza, Fiorella Faienza, Alex Frias, Shawez Khan, Matteo Bordi, Maria P. Pacheco, Julie Di Martino, Jose J. Bravo-Cordero, Colin J. Daniel, Rosalie C. Sears, Marco Donia, Daniel H. Madsen, Per Guldberg, Giuseppe Filomeni, Thomas Sauter, Caroline Robert, Daniela De Zio, Francesco Cecconi

**Affiliations:** 1 Melanoma Research Team, Cell Stress and Survival Unit, Center for Autophagy, Recycling and Disease (CARD), Danish Cancer Society Research Center, Copenhagen, Denmark; 2 Lab of Translational Cell and Tissue Research, University of Leuven, Leuven, Belgium; 3 INSERM U981, Gustave Roussy Institute, Villejuif, France; 4 National Center for Cancer Immune Therapy, Department of Oncology, Copenhagen University Hospital, Herlev, Denmark; 5 Redox Biology Group, Danish Cancer Society Research Center, Copenhagen, Denmark; 6 Department of Biology, University of Rome Tor Vergata, Rome, Italy; 7 Department of Pediatric Hematology and Oncology, Bambino Gesù Children’s Hospital, Rome, Italy; 8 Life Sciences Research Unit, University of Luxembourg, Belvaux, Luxembourg; 9 School of Medicine, Division of Hematology and Oncology, The Tisch Cancer Institute, Icahn School of Medicine at Mount Sinai, New York, NY USA; 10 Department of Molecular and Medical Genetics, Oregon Health & Science University, Portland, OR USA; 11 Knight Cancer Institute, Oregon Health & Science University, Portland, OR USA; 12 Molecular Diagnostics Group, Danish Cancer Society Research Center, Copenhagen, Denmark; 13 Department of Cancer and Inflammation Research, Institute for Molecular Medicine, University of Southern Denmark, Odense, Denmark; 14 Center for Healthy Aging, University of Copenhagen, Copenhagen, Denmark; 15 Université Paris-Sud, Université Paris-Saclay, Kremlin-Bicêtre, France; 16 Dermato-Oncology, Gustave Roussy Cancer Campus, Villejuif, France; 17 Cell Stress and Survival Unit, Center for Autophagy, Recycling and Disease (CARD), Danish Cancer Society Research Center, Copenhagen, Denmark

**Keywords:** Melanoma, Focal adhesion, Cell invasion

## Abstract

Melanoma is the deadliest skin cancer. Despite improvements in the understanding of the molecular mechanisms underlying melanoma biology and in defining new curative strategies, the therapeutic needs for this disease have not yet been fulfilled. Herein, we provide evidence that the Activating Molecule in Beclin-1-Regulated Autophagy (Ambra1) contributes to melanoma development. Indeed, we show that *Ambra1* deficiency confers accelerated tumor growth and decreased overall survival in *Braf/Pten*-mutated mouse models of melanoma. Also, we demonstrate that *Ambra1* deletion promotes melanoma aggressiveness and metastasis by increasing cell motility/invasion and activating an EMT-like process. Moreover, we show that *Ambra1* deficiency in melanoma impacts extracellular matrix remodeling and induces hyperactivation of the focal adhesion kinase 1 (FAK1) signaling, whose inhibition is able to reduce cell invasion and melanoma growth. Overall, our findings identify a function for AMBRA1 as tumor suppressor in melanoma, proposing FAK1 inhibition as a therapeutic strategy for AMBRA1 low-expressing melanoma.

## Abstract (web summary)

The absence of scaffold protein Ambra1 leads to hyperproliferation and growth in mouse models. Here the authors show that Ambra1 deficiency accelerates melanoma growth and increases metastasis in mouse models of melanoma through FAK1 hyperactivation.

## Introduction

Melanoma is the most aggressive and deadly type of skin cancer. If not diagnosed early, melanoma becomes highly metastatic. Despite its heterogeneity, which is largely due to its high mutational burden, alterations of key genes of the mitogen-activated protein pinase (MAPK) and the phosphatidylinositol-3-kinase/AKT serine/threonine kinase 1 (PI3K/AKT) pathways have been identified as hallmarks of melanoma and have contributed to improve patient stratification^1,2^. Among these genes, v-raf murine sarcoma viral oncogene homolog (*BRAF*) mutations (e.g., the V600E substitution) are the most frequent and reported in >50% of patients with melanoma^3^. Additionally, further genetic modifications may alternatively or concomitantly affect other players of the MAPK and PI3K/AKT pathway, such as neuroblastoma RAS viral oncogene homolog (*NRAS*), Neurofibromin 1 (*NF1*) and phosphatase and tensin homolog (*PTEN*)^3^.

In recent years, efforts to understand the molecular mechanisms underlying melanoma ontogenesis and progression, as well as develop new therapeutic approaches, have been intensified^4–6^. Treatment of advanced melanoma has been improved considerably with the use of (i) targeted therapy, designed to target the most common oncogenic mutations (e.g., BRAF/MEK inhibitors); and (ii) immunotherapy, which aims at stimulating the anti-tumor immune response mainly by T-cell reactivation (e.g., immune checkpoint inhibitors)^4,5^. Despite these efforts, melanoma treatment remains challenging because of the strong metastatic propensity and the acquisition of resistance to therapy, which are distinctive features of this disease.

Ambra1 is a multifunctional scaffold protein whose functional deficiency induces neuroepithelial hyperplasia associated with autophagy impairment in mouse embryos^7,8^. Indeed, AMBRA1 is considered as a pro-autophagic protein, since it facilitates autophagy initiation by regulating Beclin1 and Unc-51 like autophagy activating kinase (ULK1) activity^7–9^. Furthermore, due to the great flexibility of its structure, which explains its ability to interact with many molecular partners, AMBRA1 can regulate a plethora of biological processes, spanning from apoptosis to cell proliferation^10^. Importantly, Ambra1 has been proposed to act as tumor suppressor, since *Ambra1* absence and haploinsufficiency, respectively, lead to cellular hyperproliferation and onset of spontaneous tumors in animal models^11^. In particular, we discovered that AMBRA1 controls cell proliferation and cell cycle progression by regulating c-Myc stability through its interaction with the protein phosphatase 2A (PP2A)^11^ and, more recently, also the Cyclin D1 stability by interacting with the E3 ligase DDB1/Cullin4^12^.

Recently, it was reported that concomitant loss of AMBRA1 and Loricrin (a major component of cornified cells) expression in the peritumoral epidermis represents a prognostic biomarker for early-stage melanoma and high-risk tumor subsets, independently from tumor invasion depth^13,14^. Although this can argue for AMBRA1 being prognostic of melanoma outcome, a direct role of AMBRA1 in this disease has not yet been investigated. Indeed, AMBRA1 expression levels in the tumor tissue have not yet been associated with any stage/feature of melanoma.

Here, by applying the preclinical *Braf*^*V600E*^/*Pten*-deleted mouse model of melanoma^15^ combined with the conditional knock-out of *Ambra1*^16^, we provide evidence that *Ambra1* impacts on melanoma development. In particular, we show that the absence of *Ambra1* promotes the formation of melanocytic nevi and accelerates melanoma growth, eventually enhancing metastatic potential.

## Results

### *Ambra1* deficiency promotes melanoma development in *Braf*^*V600E/+*^*;Pten*^*−/−*^ mice

**Fig. 1:**
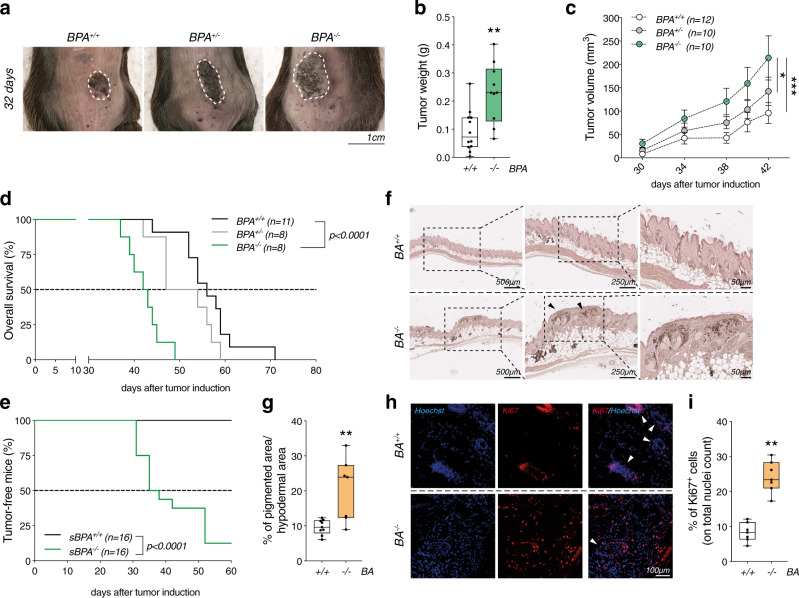
*Ambra1* deletion promotes melanoma development in *Braf*^*V600E/+*^*;Pten*^*−/−*^ mice. **a** Representative pictures of tumors from *BPA*^*+/+*^, *BPA*^*+/−*^ and *BPA*^*−/−*^ mice at 32 days after treatment. **b** Weight of *BPA*^*+/+*^ (*n* = 12) and *BPA*^*−/−*^ (*n* = 10) tumors at endpoint (42 days) (**p = 0.0053, two-tailed unpaired *t*-test, *BPA*^*−/−*^ vs. BPA^*+/+*^). **c** Kinetics of tumor growth in *BPA*^*+/+*^ (*n* = 12), *BPA*^*+/−*^ (*n* = 10) and *BPA*^*−/−*^ (*n* = 10) mice. Each data point corresponds to the average tumor volume ± SEM for each experimental group (*p = 0.0128; ***p = 0.001, one-way ANOVA). **d** Kaplan–Meier overall survival curves of *BPA*^*+/+*^ (*n* = 11), *BPA*^*+/−*^ (*n* = 8) and *BPA*^*−/−*^ (*n* = 8) mice (*p* < *0.0001*, Two-sided log-rank test, *BPA*^*−/−*^ vs. *BPA*^*+/+*^) and **e** of mice subcutaneously injected with 2×10^6^ *BPA*^*+/+*^- (*sBPA*^*+/+*^; *n* = 16) or *BPA*^*−/−*^- (*sBPA*^*−/−*^; *n* = 16) tumor-derived cells. Tumors appearance was monitored for 60 days (*p* < *0.0001*, Two-sided log-rank test, *sBPA*^*−/−*^ vs. *sBPA*^*+/+*^). **f** Areas of hyperplasia (black arrows) and of pigmentation in skin sections of *BA*^*+/+*^ (*n* = 9) and *BA*^*−/−*^ (*n* = 7) mice are shown and **g** quantified. Each data point represents one mouse and corresponds to the average percentage ± SEM of the pigmented area (at least four regions analyzed for each mouse) (**p = 0.0033, two-tailed unpaired *t*-test, *BA*^*−/−*^ vs*. BA*^*+/+*^). **h** Representative images of Ki67 immunostaining (red) in skin sections of *BA*^*+/+*^ (*n* = 7) and *BA*^*−/−*^ (*n* = 7) mice. Nuclei are shown in blue (Hoechst). White arrows indicate hair bulbs. **i** Quantification of the signal shown in **h**. Each data point represents one mouse and corresponds to the average ratio ± SEM of Ki67^+^ cells on the nuclei count (at least three fields analyzed for each mouse) (**p = 0.006, two-tailed unpaired *t*-test, *BA*^*−/−*^ vs. *BA*^*+/+*^). Box-and-whisker plots shown in (**b**, **g**, **i**) represent values from minimum to maximum. Top and bottom whiskers denote upper (Q_3_) and lower (Q_1_) quartiles, respectively. Boxes refer to interquartile ranges. Medians are denoted as horizontal line in the middle of the boxes.

To assess the role of *Ambra1* in *Braf/Pten*-driven melanoma, *Ambra1*^*flox/flox*^ mice^16^ were crossed with mice carrying *Braf*^*V600E*^ mutation and *Pten* deletion (*Braf*^*V600E/+*^*;Pten*^*flox/flox*^)^15^, a genetic combination that induces melanoma with 100% penetrance in mice^17,18^. In this genetically engineered mouse model (GEMM), melanocyte-specific Cre recombination is controlled by the tyrosinase promoter (*Tyr::CreER*^*T2/+*^) and occurs only upon 4-hydroxytamoxifen (4-OHT) administration. Three-week-old *Tyr::CreER*^*T2/+*^*;Braf*^*V600E/+*^*;Pten*^*−/−*^*;Ambra1*^*+/+*^ (*BPA*^*+/+*^), *Tyr::CreER*^*T2/+*^*;Braf*^*V600E/+*^*;Pten*^*−/−*^*;Ambra1*^*+/−*^ (*BPA*^*+/−*^) and *Tyr::CreER*^*T2/+*^*;Braf*^*V600E/+*^*;Pten*^*−/−*^*;Ambra1*^*−/−*^ (*BPA*^*−/−*^) mice were locally treated with 4-OHT on the skin of the lower back to initiate melanoma (Supplementary Figs. 1a and 2a). Noticeably, *BPA*^*−/−*^ mice developed larger tumors (Fig. 1a, b) with a higher growth rate (Fig. 1c) than *BPA*^*+/+*^ mice. The median survival of *BPA*^*−/−*^ mice also significantly decreased from 56 to 42 days (Fig. 1d). However, the morphology of these tumors did not differ in terms of myxoid, S100^+^ and pigmented cells (Supplementary Fig. 2b, c). Notably, we observed an *Ambra1* dose-dependent impact on melanoma development, as showed by tumor growth and median survival of *Ambra1* heterozygous (*BPA*^*+/−*^) mice (Fig. 1a, c, d). Nevertheless, as the complete depletion of *Ambra1* resulted in stronger effects, *BPA*^*+/−*^ tumors were excluded from further analyses.

Additionally, the faster kinetics of growth was confirmed in a syngeneic mouse model, in which mice were subcutaneously injected with two different amounts of cells derived from *BPA*^*+/+*^ (*sBPA*^*+/+*^) and *BPA*^*−/−*^ (*sBPA*^*−/−*^) tumors and followed up for 60 days (Fig. 1e) and 120 days (Supplementary Fig. 2d). In this timeframe, 87.5% and 80% of *sBPA*^*−/−*^ mice developed tumors, respectively, whereas no tumors were observed in *sBPA*^*+/+*^ mice.

To test whether the loss of *Ambra1* was sufficient to lead to melanoma formation in *Braf*^*V600E*^-mutated melanocytes^18^, *Tyr::CreER*^*T2/+*^*;Braf*^*V600E/+*^*;Ambra1*^*+/+*^ (*BA*^*+/+*^) and *Tyr::CreER*^*T2/+*^*;Braf*^*V600E/+*^*;Ambra1*^*−/−*^ (*BA*^*−/−*^) mice were treated with 4-OHT on the dorsal skin at postnatal days 1, 3, and 5 (Supplementary Fig. 1b). Thirteen weeks after 4-OHT induction, skin sections from *BA*^*−/−*^ mice displayed a significant increase in pigmentation (melanocytic nevi) and hyperplastic regions at higher magnification (Fig. 1f, g). These phenotypes were associated with a marked increase in Ki67 immunostaining, suggesting that *BA*^*−/−*^ melanocytes proliferated more than *BA*^*+/+*^ counterparts (Fig. 1h, i). Ki67^+^ cells were mainly located at the highly pigmented areas in *BA*^*−/−*^ skin sections, whereas Ki67 positivity was mostly detected at the hair bulbs of *BA*^*+/+*^ mice (Supplementary Fig. 2e). Considering the role of Ambra1 on c-Myc degradation via interaction with PP2A^11^ and its newly discovered function in controlling cell cycle progression by regulating Cyclin D1 stability^12^, we sought to investigate whether the hyperproliferation observed in *Braf*-mutated melanocytes in the absence of *Ambra1* could be related to c-Myc and Cyclin D1 regulation. Interestingly, *Ambra1*-deficient nevi displayed a significant increase in Cyclin D1 levels, and a slight not significant upregulation of phosphorylated c-Myc (p-c-Myc-S62) (Supplementary Fig. 2f–h). *BA*^*+/+*^ and *BA*^*−/−*^ mice were observed over a long period of time (up to 350 days) to monitor for melanoma onset. Tumor penetrance was low in *BA*^*−/−*^ mice (12.5%) (Supplementary Fig. 2k, Supplementary Table 1), and no tumors were observed in *BA*^*+/+*^ mice (Supplementary Table 1).

These results suggest that *Ambra1* deficiency promotes melanoma growth in a *Braf*^*V600E/+*^;*Pten*^*−/−*^ genetic background. Also, it affects pigmentation and induces hyperplasia in the skin in combination with the *Braf*^*V600E*^ mutation alone.

### *Ambra1* deficiency affects proliferation in *Braf*^*V600E/+*^*;Pten*^*−/−*^ mice

**Fig. 2:**
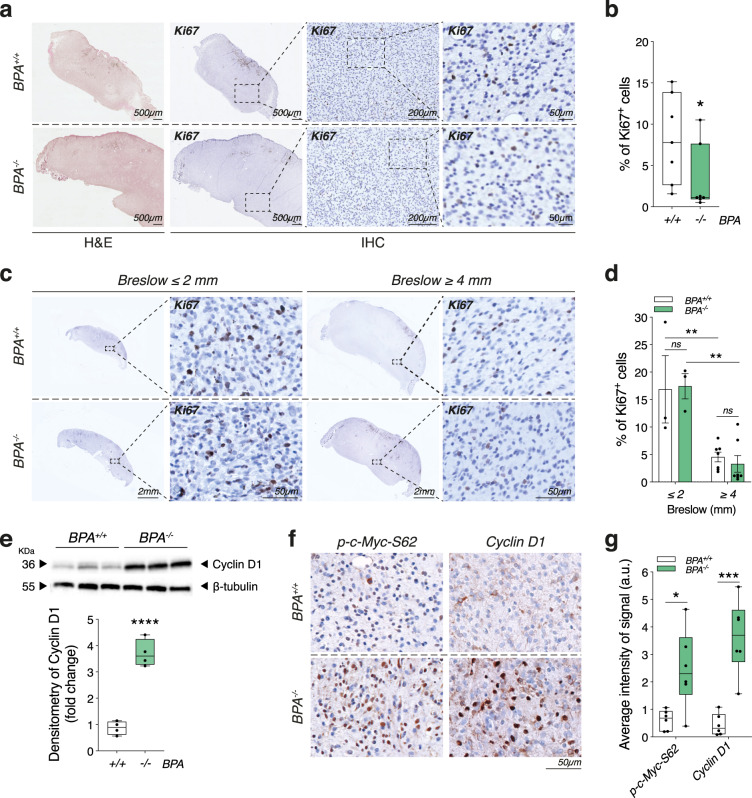
Cell proliferation upon *Ambra1* deletion. **a** Representative IHC staining of Ki67 on FFPE-tumor sections of *BPA*^*+/+*^ (*n* = 7) and *BPA*^*−/−*^ (*n* = 7) mice. **b** Quantification of the Ki67^+^ cells shown in **a**. Each data point represents one mouse and corresponds to the average ratio ±  SEM of Ki67^+^ cells on nuclei count (at least three sections analyzed for each mouse) (*p = 0.0262, two-tailed unpaired *t*-test, *BPA*^*−/−*^ vs. *BPA*^*+/+*^). **c** Representative IHC staining of Ki67 on tumors collected at Breslow ≤2 mm (average = 1.79 ± 0.28 mm; *BPA*^*+/+*^ *n* = 3, *BPA*^*−/−*^ *n* = 3) and ≥4 mm (average = 4.24 ± 0.69 mm; *BPA*^*+/+*^ *n* = 7, *BPA*^*−/−*^ *n* = 7). **d** Quantification of Ki67^+^ cells shown in **c**. Each data point represents one mouse and corresponds to the average ratio ±  SEM of Ki67^+^ cells on nuclei count for each mouse (**p = 0.0045 *BPA*^*+/+*^ ≥ 4 mm vs. *BPA*^*+/+*^ ≤ 2 mm; **p = 0.0015 *BPA*^*−/−*^ ≥ 4 mm vs. *BPA*^*−/−*^ ≤ 2 mm, two-way ANOVA). **e** Representative WB analysis and quantification (box plot) of Cyclin D1 in *BPA*^*+/+*^ (*n* = 4) and *BPA*^*−/−*^ (*n* = 4) bulk tumor lysates. ß-tubulin was used as loading control (****p < 0.0001, two-tailed unpaired *t*-test, *BPA*^*−/−*^ vs. *BPA*^*+/+)*^. **f** Representative IHC staining of Cyclin D1 and p-c-Myc-S62 in FFPE-tumor sections of *BPA*^*+/+*^ (*n* = 6) and *BPA*^*−/−*^ (*n* = 6) mice. **g** Quantification of Cyclin D1 and p-c-Myc-S62 signals shown in **f** (*p = 0.0117; ***p = 0.0002, two-tailed unpaired *t*-test, *BPA*^*−/−*^ vs*. BPA*^*+/+*^). Box-and-whisker plots shown in **b**, **e**, **g** represent values from minimum to maximum. Top and bottom whiskers denote upper (Q_3_) and lower (Q_1_) quartiles, respectively. Boxes refer to interquartile ranges. Medians are denoted as horizontal line in the middle of the boxes.

Despite the faster growth kinetics of *Ambra1*-deficient tumors, the proliferation rate of *BPA*^*−/−*^ mice analyzed at the final endpoint (i.e., after 42 days, when maximum tolerated tumor size was reached) appeared slightly, but significantly, decreased (Fig. 2a, b). The relative amount of Ki67^+^ cells in *Ambra1*-deficient tumors was, indeed, reduced if compared to their *wild-type* counterparts at the same time point (42 days) (Fig. 2a, b). To assess whether this seemingly reduced cell proliferation was due to increased cell death, we performed TUNEL assays and western blot (WB) analyses of the apoptotic marker Casp3 in *BPA*^*+/+*^ and *BPA*^*−/−*^ tumor sections and extracts. No significant difference between the two genotypes was observed (Supplementary Fig. 2l, m), except for a lower number of total nuclei present in *BPA*^*−/−*^ tumors (Supplementary Fig. 2n), which was in line with the reduced proliferation rate. However, when Ki67 analysis was performed in *BPA*^*+/+*^ and *BPA*^*−/−*^ tumors of same size (e.g., Breslow thickness)—either after tumor appearance (≤2 mm), or when tumors reached maximum allowed size (≥ 4 mm)—we did not observe any significant difference between the two groups (Fig. 2c, d), suggesting that *BPA*^*+/+*^ and *BPA*^*−/−*^ tumors actually showed similar proliferation rates. Nevertheless, it should be noted that, independently on the genotype, bigger tumors (i.e., ≥4 mm) were characterized by an overall reduction of proliferation rate than smaller tumors (≤2 mm) (Fig. 2c, d). These results clearly indicate that the number of proliferating melanoma cells do not change if normalized on tumor size. Therefore, the larger volume of *BPA*^*−/−*^ tumors (Fig. 1c) can be only ascribed to a proliferative advantage conferred by the loss of *Ambra1*, that allows them to reach the maximum size in a shorter timeframe. In support to this hypothesis, we observed that *BPA*^*−/−*^ tumors showed increased c-Myc phosphorylation and Cyclin D1 levels (Fig. 2e–g). This additional evidence is in agreement with results provided elsewhere^11,12^, and indicates that loss of *Ambra1* likely promotes melanoma growth by increasing cell proliferation.

### *Ambra1* deficiency promotes an invasive phenotype in *Braf*^*V600E/+*^;*Pten*^*−/−*^ mice

**Fig. 3:**
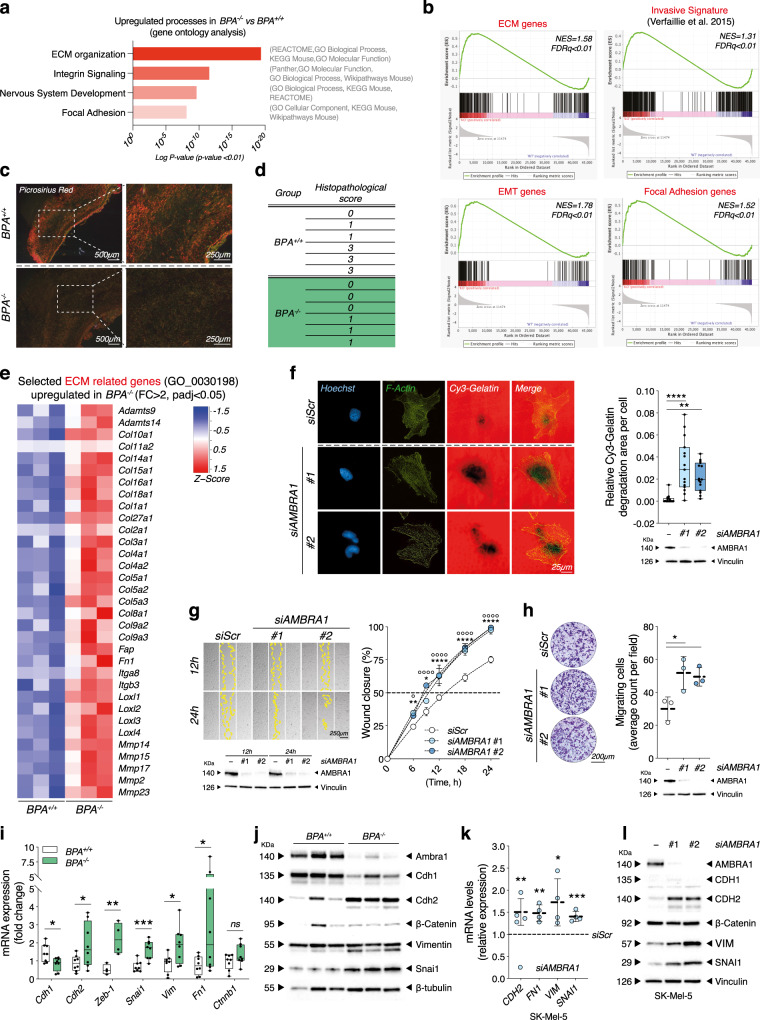
*Ambra1* deletion induces an invasive phenotype in *Braf*^*V600E/+*^*;Pten*^−/−^ mice. **a** Upregulated processes (*p* < 0.01) and **b** GSEA (*NES*, normalized enrichment score; *FDR*, false discovery rate) in *BPA*^−/−^ (*n* = 3) vs. *BPA*^*+/+*^ (*n* = 3) tumors. **c** Representative images and **d** histopathological evaluation of *Picrosirius Red* staining in *BPA*^*+/+*^ *(n* = 6) and *BPA*^−/−^ (*n* = 6) tumors. **e** Differential expression of selected ECM-related genes in tumors (*n* = 3 each group, *FC* > 2, *padj* < 0.05.) **f** Gelatin degradation in AMBRA1-silenced SK-Mel-5 cells. F-Actin and nuclei were counterstained using Phalloidin and Hoechst. Images are representative. Each dot represents the average count per field ± SD (*n* = 3; **p = 0.0022; ****p < 0.0001, one-way ANOVA). **g** Wound closure area in AMBRA1-silenced SK-Mel-5. Quantification is shown as percentage ± SD vs. *T*_0_ at indicated times. White and yellow lines outline wound edge at *T*_0_ and at the times indicated. Images are representative (*n* = 4; **p = 0.0021 6 h; *p = 0.026 9 h; ****p < 0.0001 12 h, 18 h and 24 h, *siAMBRA1#1* vs. *siScr*. *n* = 3; *°p* = 0.0234 6 h; *°°°°p* < 0.0001 9 h, 12 h, 18 h and 24 h, *siAMBRA1#2* vs*. siScr*, two-way ANOVA). **h** Count of migrating cells in AMBRA1-silenced SK-Mel-5. Data are expressed as mean ± SD, images representative (*n* = 3; *p = 0.0289 *siAMBRA1#1* vs*. siScr*, *p = 0.0454 *siAMBRA1#2* vs. *siScr*, one-way ANOVA). WB in **f**–**h** indicate silencing efficiency. **i** RT-qPCR analyses of *Cdh1*, *Cdh2*, *Zeb-1*, *Snai1*, *Vim*, *Fn1* and *Ctnnb1* in *BPA*^−/−^ (*n* = 4*–*8) and *BPA*^*+/+*^ (*n* = 4–8). Data normalized to *L34* are expressed as fold-change (*p = 0.0158 Chd1; *p = 0.0417 Chd2; **p = 0.0096 Zeb-1; ***p = 0.0006 Snai1; *p = 0.0239 Vim; *p = 0.0458 *Fn1*, two-tailed unpaired *t*-test, *BPA*^−/−^ vs. *BPA*^*+/+*^). **j** WB analyses of Cdh1, Cdh2, ß-Catenin, Vimentin and Snai1 in bulk tumors (*n* = 3 each group). **k** RT-qPCR analyses of *CDH2*, *FN1*, *VIM* and *SNAI1* i*n* AMBRA1-silenced SK-Mel-5. Data normalized to *L34* are expressed as fold-change ± SD (*n* = 4; **p = 0.0045 *CDH2*; **p = 0.0022 *FN1*; *p = 0.0353 *VIM*; ***p = 0.0005 *SNAI1*, two-tailed unpaired *t*-test, *siAMBRA1* vs. *siScr*, dashed line). **l** Representative (*n* = 4) WB analyses of CDH1, CDH2, ß-Catenin, VIM and SNAI1 in AMBRA1-silenced SK-Mel-5. Box-and-whisker plots in **f**, **i** represent minimum-to-maximum values. Top and bottom whiskers denote upper (Q_3_) and lower (Q_1_) quartiles, respectively. Boxes refer to interquartile ranges. Medians are denoted as horizontal line in the middle of the boxes.

Next, to gain further insights into the processes affected by *Ambra1* deficiency, we performed RNA sequencing of *BPA*^*+/+*^ and *BPA*^*−/−*^ tumors at the 42-day endpoint. Gene ontology analysis revealed that a number of processes, namely extracellular matrix organization (ECM), integrin signaling, nervous system development and focal adhesion genes were significantly upregulated in *BPA*^*−/−*^ tumors, as confirmed by various ontology libraries (*p* < 0.01) (Fig. 3a; Supplementary Fig. 3a–d and Supplementary Data 1). Gene set enrichment analysis (GSEA) also displayed an enrichment of gene sets related to invasiveness (invasive melanoma signature from Verfaillie et al.^19^), ECM organization (GO:0030198), epithelial-to-mesenchymal transition (EMT) (Hallmark gene set) and assembly of focal adhesions (GO:0005925) in *BPA*^*−/−*^ melanomas (Fig. 3b). Prompted by these findings, we evaluated the ECM structure of *BPA*^*+/+*^ and *BPA*^*−/−*^ tumor sections by Picrosirius Red (PR) staining. In general, tumors variably present areas with thick confluent collagen fibers, similar to the normal reticular dermis, areas where the thick collagen fibers were isolated and separated by a thin and fibrillary stroma, and areas with only thin fibrillary stroma. Histopathological analyses revealed that *BPA*^*−/−*^ melanomas had a predominant fine fibrillar stroma, whereas *BPA*^*+/+*^ tumors more often displayed longer and thicker collagen fibers (Fig. 3c, d). An upregulation of ECM-related genes, including ECM-degrading proteases, was also evident from RNAseq analyses of *BPA*^*−/−*^ melanomas (Fig. 3e), as further validated by quantitative reverse transcription PCR (RT-qPCR) for a selection of genes (Supplementary Fig. 4a). Notably, gelatin invadopodia assay showed that human SK-Mel-5 melanoma cells silenced for AMBRA1 (*siAMBRA1*) were characterized by a higher ability to degrade gelatin matrix, when compared to control (*siScr*) cells (Fig. 3f), thus highlighting possible implications of AMBRA1 deficiency in increased invasive and migratory capacity of melanoma cells. To further unravel this aspect, we performed wound-healing and transwell migration assays both ex vivo, i.e., in melanoma cells isolated from *BPA*^*+/+*^ and *BPA*^*−/−*^ mice (Bdmc^+/+^ and Bdmc^−/−^, respectively) (Supplementary Fig. 4b, c), and in vitro*,* i.e., in human melanoma cell lines upon *siAMBRA1*-mediated knockdown (Fig. 3g, h and Supplementary Fig. 4d–g). In all different setting combinations, we observed that AMBRA1-deficiency elicited faster kinetics of wound closure as well as increased migratory capacity. The gain of invasive capability was also associated with an increased expression of EMT genes (e.g., *CDH2*, *FN1*, *VIM*, *SNAI1)*, as revealed by both RT-qPCR and WB analyses of bulk tumors (Fig. 3i, j; Supplementary Fig. 4h), Bdmc^+/+^ and Bdmc^−/−^ cells (Supplementary Fig. 4i) and *siAMBRA1* human melanoma SK-Mel-5 (Fig. 3k, l and Supplementary Fig. 4j), MeWo and SK-Mel-2 cells (Supplementary Fig. 4k–m).

Altogether, these results indicate that loss of *Ambra1* promotes melanoma invasion by inducing ECM remodeling and an EMT-like phenotype.

### *Ambra1* deficiency promotes melanoma metastasis in mouse models

**Fig. 4:**
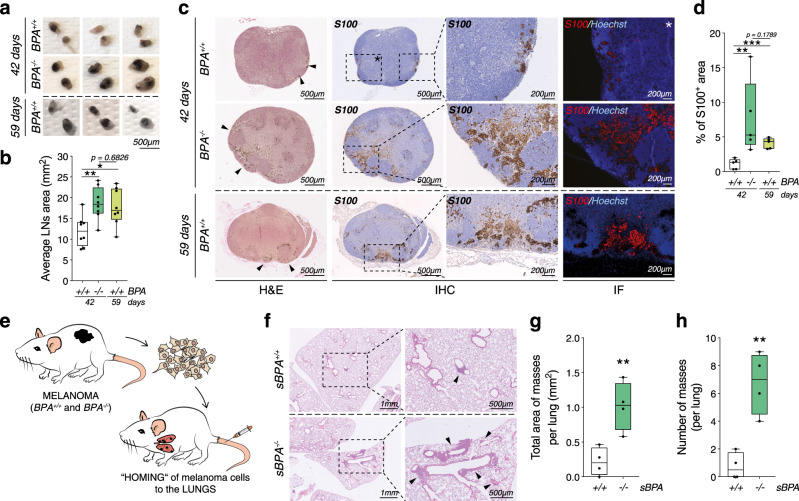
*Ambra1* deletion promotes melanoma metastases. **a** Macroscopic evaluation of inguinal lymph nodes (iLNs) collected when *BPA*^−/−^ tumors reached maximum tolerated size (42 days, *BPA*^*+/+*^, *n* = 4; *BPA*^−/−^, *n* = 4) or at maximum allowed volume of *BPA*^*+/+*^ tumors (59 days, *n* = 4). Three representative images are shown for each group. **b** Average area of iLNs was calculated at 42 (*BPA*^*+/+*^, *n* = 4; *BPA*^−/−^, *n* = 4) and 59 (*BPA*^*+/+*^, *n* = 4) days. Each point represents the average area of the iLNs for each mouse. Data are shown as mean ± SEM (*p = 0.0172; **p = 0.0063, one-way ANOVA). **c** FFPE-iLNs sections were stained with H&E (left panels) and anti-S100 (IHC, middle panels; IF, rightmost panels). Areas of metastases are indicated by black arrows. Nuclei were counterstained with Hoechst in IF staining. Images are representative of each group (*n* = 5). **d** Quantification of S100 IHC. Each data point represents the average percentage of S100^+^ area of the iLNs of each mouse ± SEM (*n* = 5, **p = 0.0079; ***p = 0.0002, one-way ANOVA). **e** Schematic representation of the syngeneic model for lung homing of melanoma cells; 0.25 × 10^6^ cells/mouse were intravenously injected. **f** Perfused FFPE-lungs were stained with H&E and areas of cells homing indicated by black arrows. Images are representative of each group (*n* = 4). **g** Total area (**p = 0.0075) and **h** number of masses (**p = 0.0025) in lungs of s*BPA*^*+/+*^ (*n* = 4) and s*BPA*^−/−^ (*n* = 4) mice were quantified (two-tailed unpaired *t*-test, *sBPA*^−/−^ vs. *sBPA*^*+/+*^). Box-and-whisker plots shown in **b**, **d**, **g**, **h**) represent values from minimum to maximum. Top and bottom whiskers denote upper (Q_3_) and lower (Q_1_) quartiles, respectively. Boxes refer to interquartile ranges. Medians are denoted as horizontal line in the middle of the boxes.

In order to address the relevance of these findings in vivo, we analyzed the ability of *Ambra1*-deficient melanoma cells to colonize other organs. Macroscopic observations and histological analyses revealed that inguinal lymph nodes (iLNs) of *BPA*^*−/−*^ mice harbored larger pigmented areas compared to *BPA*^*+/+*^ mice at 42-day endpoint (Fig. 4a–d), and showed a higher number of S100^+^ melanoma cells (Fig. 4c, d). Interestingly, histological analyses of iLNs of *BPA*^*+/+*^ mice at maximum tolerated tumor size (59 days versus 42 days of *BPA*^*−/−*^) showed a trend of increase in the pigmented areas of *BPA*^*+/+*^ similar, yet lower than that observed in *BPA*^*−/−*^ mice (Fig. 4a–d). This evidence supports the hypothesis that *Ambra1* deletion accelerates melanoma progression and promotes the invasive state of melanoma. However, when the maximum tolerated size of the primary tumors was reached, no lung metastases could be observed in either of the two groups. Therefore, to assess whether distant organs were more efficiently colonized by *Ambra1*-deficient melanoma cells, we took advantage of a syngeneic mouse model, i.e., C57Bl/6 mice injected with freshly dissociated cells from *BPA*^*+/+*^ and *BPA*^*−/−*^ tumors through the tail vein (*sBPA*^*+/+*^ and *sBPA*^*−/−*^, respectively) (Fig. 4e). The health status of *sBPA*^*+/+*^ and *sBPA*^*−/−*^ mice was monitored for 3 months, after which they were euthanized and lungs collected. Although metastases were not macroscopically visible, histological analyses revealed that lungs from *sBPA*^*−/−*^ mice exhibited increased homing and larger nodules than *sBPA*^*+/+*^ mice (Fig. 4f–h), suggesting that *Ambra1* depletion promotes melanoma metastasis.

### *Ambra1* deficiency induces hyperactivation of FAK1 signaling in melanoma

**Fig. 5:**
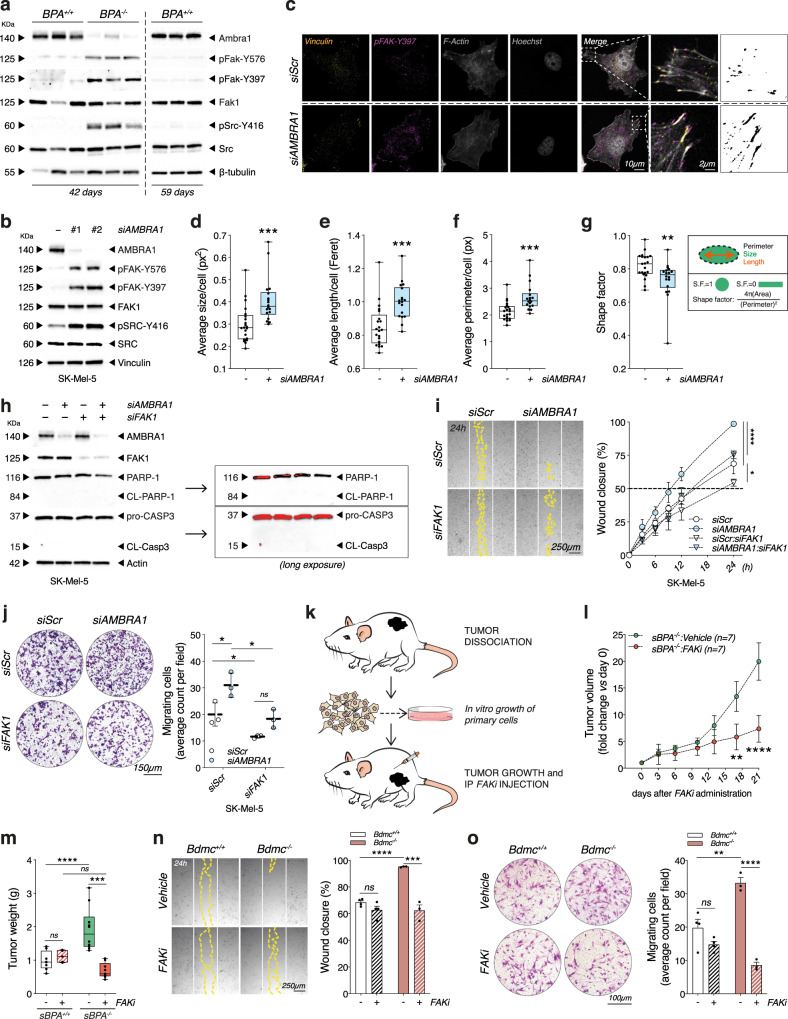
*Ambra1*-deficient melanoma displays FAK1 signaling hyperactivation. **a** WB analyses of pFak-Y576, pFak-Y397, Fak1, pSrc-Y416 and Src in 42-day *BPA*^*+/+*^/*BPA*^−/−^ and in 59-day *BPA*^*+/+*^ tumors (*n* = 3 each group) and **b** in AMBRA1-silenced SK-Mel-5 (images are representative of *n* = 4). **c** Representative (*n* = 3) IF staining of pFAK-Y397 in AMBRA1-silenced SK-Mel-5. Phalloidin, Vinculin, and Hoechst were used to counterstain F-Actin fibers, cytoskeleton and nuclei. Rightmost panels display thresholds used for quantifying **d** average size (***p = 0.001), **e** length (Feret) (***p = 0.0003), **f** perimeter (***p = 0.0005) and **g** shape factor (***p = 0.0098) of FAs (*n* = 3, two-tailed unpaired *t*-test, *siAMBRA1* vs. *siScr*). **h** Representative (*n* = 3) WB analyses of cleaved PARP-1 (CL-PARP-1) and CASP-3 (CL-CASP3) (*Long exposure*: red bands identify signal saturation) in AMBRA1/FAK1 co-silenced SK-Mel-5. **i** Wound closure was assessed in the conditions specified in **h** and shown as percentage ± SD vs. *T*_0_ at indicated times. White and yellow lines outline wound edge at *T*_0_ and at 24 h. Statistics and representative images are provided at 24 h (*n* = 3; *p = 0.037; ****p < *0.0001*, two-way ANOVA). **j** Count of migrating cells in AMBRA1/FAK1 co-silenced SK-Mel-5. Data are expressed as mean ± SD, images representative (*n* = 3; *p = 0.0246 *siScr:siAMBRA1* vs*. siScr:siScr*; *p = 0.0116 *siFAK1:siAMBRA1* vs*. siScr:siAMBRA1*; *p = 0.0304 *siScr:siFAK1* vs. *siScr:siScr*, two-way ANOVA). **k** Schematic representation of *FAKi* treatment. **l** Tumor growth kinetics in *Vehicle*- (*n* = 5) or *FAKi*- (*n* = 5) treated *sBPA*^−/−^ mice. Data points represent average volume vs*.* day 0 ± SEM (**p = 0.0022; ****p < 0.0001, two-way ANOVA). **m** Weight of *sBPA*^*+/+*^*:Vehicle* (*n* = 8), *sBPA*^*+/+*^*:FAKi* (*n* = 5), *sBPA*^*−/−*^:*Vehicle* (*n* = 10) and *sBPA*^−/−^*:FAKi* (*n* = 9) tumors at sacrifice (***p = 0.0007; ****p < 0.0001, two-way ANOVA). **n** Wound closure of Bdmc^+/+^ (*n* = 3) and Bdmc^−/−^ (*n* = 3) cells treated with 1 µM *FAKi* for 24 h. Lines are defined as in **i**, data shown as percentage ± SEM vs*. T*_0_ at 24 h, images representative (***p = 0.0003; ****p < 0.0001, two-way ANOVA). **o** Count of migrating Bdmc^+/+^ (*n* = 3) and Bdmc^−/−^ (*n* = 3) cells treated as in **n**. Data are expressed as mean ± SEM, images representative (**p = 0.0047; ****p < 0.0001, two-way ANOVA). Box-and-whisker plots in **d**–**g**, **m** represent minimum-to-maximum values. Top/bottom whiskers denote upper (Q_3_)/lower (Q_1_) quartiles. Boxes refer to interquartile ranges. Medians are denoted as horizontal line in the middle of the boxes.

Bulk RNAseq and GSEA analyses showed that focal adhesion assembly genes (GO:0005925) were upregulated in *Ambra1*-depleted tumors (Fig. 3a, b and Supplementary Fig. 3d). Recently, it has been demonstrated that AMBRA1 controls the focal adhesion kinase 1 (FAK1)-mediated signaling pathway by interacting with FAK1 and SRC ﻿in mouse squamous cell carcinoma cells^20^. Therefore, we investigated whether the invasive phenotype observed in *Ambra1*-deficent tumors correlated with the phospho-activation of FAK1 signaling axis, which was already shown to contribute to a more aggressive phenotype in melanoma^21–24^. WB analyses revealed a marked increase of the phospho-active forms of FAK1 (pFAK-Y397 and -Y576) and SRC (pSRC-Y416) in *BPA*^*−/−*^ tumors (Fig. 5a and Supplementary Fig. 5a) and in human melanoma cells silenced for AMBRA1 (Fig. 5b and Supplementary Fig. 5b–d), as well as in iLN metastasis of *BPA*^*−/−*^ (Supplementary Fig. 5e) and in melanocytic nevi of *BA*^*−/−*^ mice (Supplementary Fig. 2f, i). Immunofluorescence analyses further confirmed the hyperactivation of FAK1 in *siAMBRA1* SK-Mel-5 cells (Fig. 5c and Supplementary Fig. 5f) and revealed that pFAK-Y397 signal colocalized at the focal adhesion (FA) with FA-associated vinculin at the edge of F-Actin fibers (Fig. 5c and Supplementary Fig. 5f). Of note, AMBRA1 deficiency was associated with an increased size and length and a decreased shape factor of FAs, a feature of cancer cells characterized by a faster motility (Fig. 5d–g and Supplementary Fig. 5g–i)^25,26^.

Immunoprecipitation analyses of both endogenous (Supplementary Fig. 5j) and AMBRA1-myc-tagged-expressing (Supplementary Fig. 5k) extracts of SK-Mel-5 cells confirmed the previously reported interaction of FAK1 and AMBRA1^20^. Intriguingly, when we expressed the FAK1^*P876A/P882A*^ mutant (HA-FAK1.miRes^*AA*^)—previously reported to abrogate the interaction with AMBRA1^20^— in FAK1 depleted SK-Mel-5 melanoma cells (*siFAK1*), we observed a reduction in AMBRA1/FAK1 interaction (Supplementary Fig. 5l). This phenomenon was accompanied by an increased phospho-activation of FAK1 signaling (Supplementary Fig. 5m), and resembled what we previously observed in AMBRA1-silenced cells (Fig. 5b), suggesting that the abrogation of the FAK1/AMBRA1 interaction is responsible for FAK1 hyperphosphorylation.

It was previously reported that PP2A interacts with AMBRA1^11^ and regulates focal adhesion^27,28^. Based on this evidence, we sought to investigate whether PP2A activity could contribute to the increased phosphorylation of both FAK1 and SRC via AMBRA1. To this end, we knocked-down AMBRA1 levels in SK-Mel-5 by siRNA selectively targeting the 5’-UTR region of *AMBRA1* messenger RNA (mRNA) (*siAMBRA1 #2*) and, next, induced the expression of the PXP mutant of AMBRA1 (*AMBRA1*^*PXP*^), which has already been reported to abrogate the binding of PP2A to AMBRA1^11,16^. The expression of *AMBRA1*^*PXP*^, as well as the re-expression of WT AMBRA1 (*AMBRA1*^*WT*^), did not affect the phosphorylation state of either FAK1 or SRC (Supplementary Fig. 5n), thus excluding any involvement of AMBRA1-related PP2A activity in the regulation of FAK1 phosphorylation upon AMBRA1 depletion.

Finally, to evaluate whether the AMBRA1 pro-autophagic function participates to the hyperactivation of FAK1, we silenced the key autophagy gene *ATG7* (*siATG7*) in melanoma cells. Of note, neither the activation of FAK1 signaling (Supplementary Fig. 6a–c) nor an increase of the invasive and migratory propensity were observed in *siATG7* cells (Supplementary Fig. 6d–i), suggesting that these phenomena were not related to autophagy.

Overall, these results indicate that AMBRA1 regulates FAK1 signaling in melanoma cells independently from its PP2A- and autophagy-related functions.

### *Ambra1*-mediated regulation of FAK1 affects melanoma progression

In order to understand whether or not FAK1 played a role in the invasive and migratory ability observed upon AMBRA1 deficiency (Fig. 3g, h), we performed wound healing and transwell migration assays in *siAMBRA1* melanoma cells concomitantly downregulating FAK1 (*siAMBRA1*:*siFAK1* double knocked down) (Fig. 5h and Supplementary Fig. 7a). Optic microscopy analyses indicated that FAK1 silencing caused a rescue of the phenotype distinctive of *siAMBRA1* cells (Fig. 5i, j and Supplementary Fig. 7b, c), which, however, was unrelated to any apoptotic events (Fig. 5h and Supplementary Fig. 7a). On the other hand, the re-expression of the HA-FAK1.miRes^*AA*^ mutant resulted in a higher migratory capacity than that induced by WT FAK (HA-FAK1.miRes) (Supplementary Fig. 7d), supporting the relevance of the AMBRA1-FAK1 interaction in determining the invasive ability of melanoma cells in our settings.

Next, in order to address the therapeutic relevance of this finding, we tested whether the pharmacological inhibition of FAK1 (*FAKi*) could specifically affect the tumor growth in mice subcutaneously injected with melanoma cells derived from *BPA*^*−/−*^ (*sBPA*^*−/−*^) tumors (Fig. 5k). Since *sBPA*^*+/+*^ mice were refractory to develop tumors (Fig. 1e and Supplementary Fig. 2d), we decided to increase the numbers of cells (up to 5 × 10^6^) to produce detectable tumors in both genetic backgrounds. Remarkably, the kinetics of tumor growth was dramatically reduced upon *FAKi* administration in *sBPA*^*−/−*^ mice (Fig. 5l and Supplementary Fig. 7e–g), as also demonstrated by reduced tumor weight at the time of collection (Fig. 5m), underlining the key role of the FAK1 signaling axis in sustaining *Ambra1*-deficient tumors.

To determine whether the inhibition of FAK1 could also affect the invasive/migratory capacity ex vivo, Bdmc^+/+^ and Bdmc^−/−^ cells were treated with the maximum non-lethal dose of *FAKi* (1 µM) for 24 h (Supplementary Fig. 7h). Consistent with our hypothesis, wound-healing and transwell migration assays revealed a statistically significant rescue of the migrative and invasive phenotypes only in Bdmc^−/−^ cells treated with *FAKi* (Fig. 5n, o).

Overall, these findings suggest that AMBRA1-mediated regulation of FAK1 signaling affects the invasive capacity of melanoma cells and impacts tumor growth in vivo.

### Low expression of AMBRA1 in human melanoma is associated with an invasive phenotype and sensitivity to FAK inhibition

**Fig. 6:**
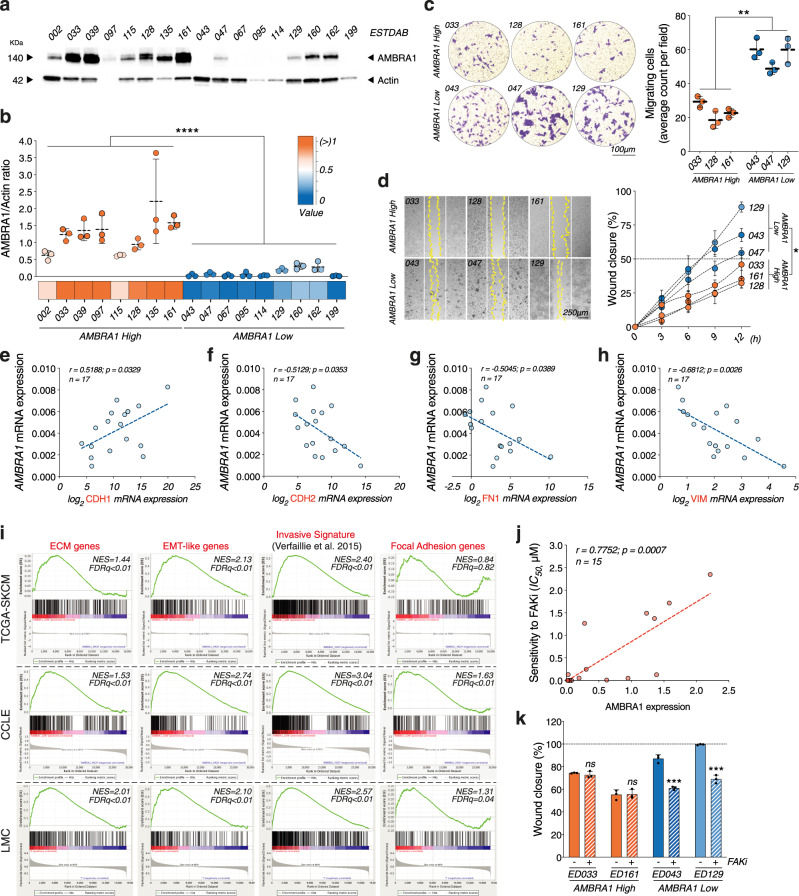
Low AMBRA1 expression in human melanoma correlates with invasive gene signature and sensitivity to FAKi. **a** WB analyses of AMBRA1 in lysates from 17 melanoma cell lines. Actin was used as a loading control. Image is representative of *n* = 3 gels. **b** AMBRA1 High and Low groups were determined by densitometric analyses of AMBRA1, expressed as AMBRA1/Actin ratio ±  SD (*n* = 3; ****p < *0.0001*, two-tailed unpaired *t*-test). **c** Count of migrating AMBRA1 High (*n* = 3) and Low (*n* = 3) cells. Data are expressed as mean ± SD and images representative (**p = 0.0026, two-tailed unpaired *t*-test, AMBRA1 Low vs. AMBRA1 High). **d** Wound closure of AMBRA1 High (*n* = 3) and Low (*n* = 3) cells is shown as percentage ± SD vs. *T*_0_ at 12 h. White and yellow lines outline wound edge at *T*_0_ and at 24 h. Statistics and representative images are provided at 12 h (*p = 0.0387, two-tailed unpaired *t*-test, AMBRA1 Low vs*.* AMBRA1 High). **e**–**h** Correlative RT-qPCR analyses of *AMBRA1* and **e** *CDH1* (*n* = 17; *r* = 0.5188; *p* = 0.0329, two-tailed Pearson correlation), **f** *CDH2* (*n* = 17; *r* = −0.5129; *p* = 0.0353, two-tailed Pearson correlation), **g** *FN1* (*n* = 17; *r* = −0.5045; *p* = 0.0389, two-tailed Pearson correlation) and **h** *VIM* (*n* = 17; *r* = *−*0.6812; *p* = 0.0026, two-tailed Pearson correlation). Data were normalized to *L34*. **i** GSEA of transcriptomic data from melanoma samples of The Cancer Genome Atlas (TCGA-SKCM), melanoma cell lines of The Cancer Cell Line Encyclopedia (CCLE) datasets and melanoma samples of the Leeds Melanoma Cohort (LMC). ECM organization (GO:0030198), Invasive signature^19^, EMT (Hallmark gene set) and Focal Adhesion Assembly (GO:0005925) gene sets in AMBRA1_Low vs. AMBRA1_High in TCGA-SKCM (*n* = 70 for each subgroup), CCLE (*n* = 7 for each subgroup) and LMC (*n* = 105 for each subgroup). *NES*, normalized enrichment score; *FDR*, false discovery rate. **j** Correlative dose-response analyses between AMBRA1 expression and sensitivity to *FAKi* (IC_*50*_, µM) in *n* = 15 melanoma cell lines (*r* = 0.7752; *p* = 0.0007, two-tailed Pearson correlation). **k** Area of wound closure of two selected AMBRA1 High and Low cells upon *FAKi*, shown as percentage ± SD with respect to *T*_0_ at 24 h. (*n* = 3; ***p = 0.0003 for ED043; ***p = 0.0001 for ED129, two-tailed unpaired *t*-test, *FAKi* vs*. Vehicle*).

To strengthen the relevance of our findings, 17 human melanoma cell lines were examined for AMBRA1 expression. Nine of these cell lines were characterized by reduced expression of AMBRA1 (Fig. 6a, b). Transwell migration and wound-healing assay of selected AMBRA1 High- and Low-expressing cell lines revealed a clear inverse correlation between AMBRA1 expression and invasive/migratory capacity (Fig. 6c, d). Also, *AMBRA1* expression negatively correlated with the expression of the mesenchymal genes *CDH2*, *FN1* and *VIM*, whereas a positive correlation was evident for the epithelial marker *CDH1* (Fig. 6e–h), hence suggesting that an active EMT-like process is associated with low AMBRA1 expression in human melanoma cells.

Transcriptomic data from melanoma samples of The Cancer Genome Atlas (TCGA-SKCM) dataset (*n* = 473), melanoma cell lines of The Cancer Cell Line Encyclopedia (CCLE) dataset (*n* = 49) and primary melanoma samples of the Leeds Melanoma Cohort (LMC, *n* = 703)^29^ were also analyzed. In all datasets, samples were ranked according to their *AMBRA1* mRNA levels. AMBRA1_High and AMBRA1_Low subgroups were defined considering the top 15% and the bottom 15% of the population in terms of *AMBRA1* mRNA expression, respectively. Intriguingly, GSEA of all datasets revealed that AMBRA1_Low subgroups were enriched for ECM, EMT and FA genes, as well as for genes linked to invasiveness in melanoma^19^ (Fig. 6i).

Finally, we sought to assess whether the expression levels of AMBRA1 could differently affect the sensitivity of human melanoma cell lines to *FAKi*. To this aim, we treated a panel of 15 melanoma cell lines with increasing doses of *FAKi* for 24 h (Supplementary Fig. 8a) and revealed a striking correlation between AMBRA1 protein expression levels and sensitivity to *FAKi* (Fig. 6j). Moreover, we also observed that non-lethal doses of *FAKi* were effective (Supplementary Fig. 8b, c) and distinctly altered the invasive/migratory capacity of selected AMBRA1-low-expressing human melanoma cell lines (Fig. 6k and Supplementary Fig. 8d).

Overall, this evidence indicates that low expression of *AMBRA1* correlates with an invasive phenotype and increased sensitivity to *FAKi* in human melanoma cells.

## Discussion

**Fig. 7:**
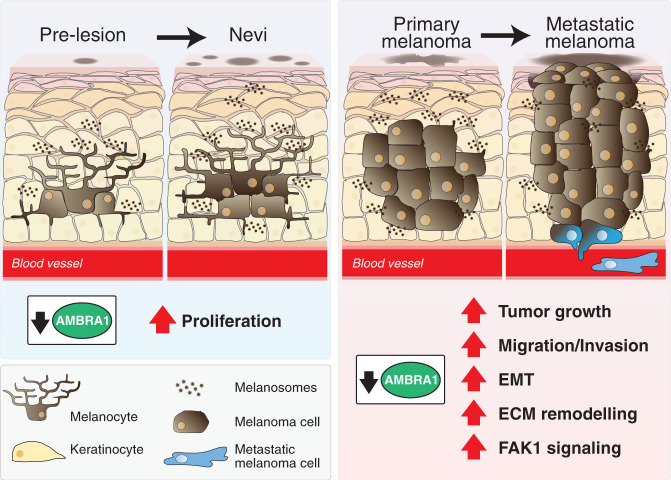
*Ambra1* impacts on melanoma development. *Ambra1* deficiency increases the formation of nevi by affecting melanocytes proliferation in *Braf*^*V600E*^ mutated mice. When melanoma is induced upon *Braf*^*V600E*^ and *Pten* ablation, the absence of *Ambra1* promotes tumor growth and metastasis by increasing cell migration and invasion, activating the EMT process, remodeling the ECM and triggering the FAK1 signaling pathway.

The results herein reported argue for a major role of *Ambra1* in the different phases of melanoma development, as demonstrated in the *Braf/Pten* mouse models of melanoma, which recapitulate key pathophysiological aspects of the human disease^15^. In particular, we found that *Ambra1* deficiency accelerates tumor growth and leads to an earlier invasive and aggressive phenotype of *Braf/Pten* melanoma. Specifically, we have shown that *Ambra1*-deficient melanomas are larger and display faster growth kinetics, with reduced overall mice survival, and melanoma cells showing higher capability to affect ECM architecture, migrate and invade, eventually enhancing melanoma metastasis (Fig. 7).

AMBRA1 exerts most of its functions as a scaffold protein, interacting with different partners and modulating various cellular processes, including apoptosis, autophagy and cell proliferation^10,12^. Consistent with AMBRA1 involvement in many aspects of a cell life, it has emerged that mouse haploinsufficiency of *Ambra1* promotes spontaneous tumorigenesis in several organs. Indeed, Ambra1’s role as tumor suppressor gene has been directly linked to its interaction with PP2A and DDB1/Cullin4 with their respective regulation of c-Myc^11^ and Cyclin D1^12^, thus indicating Ambra1 as a negative regulator of cell proliferation. Considering that we observed: (i) increased hyperpigmentation in the murine skin of mice with *Braf*^*V600E*^ background, and (ii) large-sized and early tumors in *Braf/Pten*-driven mouse models of melanoma upon *Ambra1* deficiency, we investigated and related the proliferative advantage of *Ambra1*-deficient melanocytes and melanoma to both c-Myc and Cyclin D1.

It is well known that melanoma is characterized by a high tumor heterogeneity and plasticity and that a phenotype rewiring from a high-proliferative/low-invasive to a low-proliferative/high-invasive state may occur^30,31^. These two states are associated with different phases of melanoma progression and response to therapy^32,33^. In particular, it has been shown that phenotype switching towards a slow-cycling/high-invasive state correlates with increased melanoma aggressiveness, metastasis formation and resistance to BRAF inhibitors (BRAFi)^33–37^. Of the highest importance, our data clearly show that loss of *Ambra1* not only accelerates the tumor growth, but also promotes the invasive state of melanoma. Indeed, *Ambra1*-deficient tumors displayed increased metastatic dissemination to local lymph nodes and increased homing of melanoma cells to the lungs. Consistently, AMBRA1-silenced cells were more prone to invade and migrate. Phenotype switching in melanoma is regulated by key transcriptional factors, e.g., the melanoma transcriptional master regulator microphthalmia-associated transcription factor (Mitf)^38,39^ and the nerve growth factor receptor CD271 (Ngfr)^40^, the latter promoting an invasive and metastatic cell behavior accompanied by upregulation of neural crest stem cell genes^32,34,35,39^. Consistent with the anti-invasive role of Ambra1, *Ambra1*-deficient tumors show an increase in the expression of *Ngfr* and other neural crest-related genes, which was confirmed by the upregulation of the nervous system development GO process (Fig. 3a and Supplementary Fig. 3e). This suggests that, at least in the case of melanoma, the pro-invasive phenotype of *Ambra1*-null cells may associate with tumors in a more de-differentiated state.

The more invasive state of *Ambra1*-deficient tumors was accompanied by other key processes specific to high-invasive melanomas, such as ECM proteolysis/remodeling, EMT activation and FA activation^19,23,41^. Schoenherr and colleagues have recently shown that AMBRA1 is involved in SRC/FAK1-dependent polarization and chemotactic invasion of cancer cells through its interaction with FAK1 and several trafficking proteins, thus regulating adhesion and migration^20^. In agreement with these data, here we have shown the in vitro and in vivo relevance of *Ambra1*-mediated regulation of FAK1 in melanoma development. Our results reveal that FAK1 signaling is hyperactivated in *BPA*^*−/−*^ melanomas, either directly or indirectly contributing to most of the phenotypes observed. Specifically, FAK1 signaling activation in cancer can modulate a plethora of oncogenic processes including EMT, cell motility and metalloproteases (MMPs) expression^42^. Consistently, we have shown that *Ambra1* loss deeply affects the melanoma microenvironment, marked by the upregulation of ECM-related genes, such as *Mmps* and lysyl-oxidases (*Loxl*). The role of these enzymes in modifying ECM architecture and promoting cancer progression has already largely been demonstrated for various tumor types^41,43,44^. Along with this, an upregulation of several types of collagen and an altered ECM architecture were observed in *Ambra1*-null melanomas, thus arguing for a more tumor-permissive environment^44,45^. As for this aspect, we have provided evidence that AMBRA1-deficient melanoma cells efficiently degrade gelatin matrix, thus pointing to their direct involvement in ECM remodeling. Although the exact mechanism(s) by which Ambra1 may regulate the ECM structure are still elusive, the massive modulation of gene expression observed in bulk RNAseq of *BPA*^*−/−*^ tumors strongly suggests that a transcriptional rewiring occurs upon *Ambra1* knock out. Of note, though these changes could be largely related to the stromal composition of the tumor (e.g., fibroblasts), the evidence that increased expression of EMT markers occurs both in human melanoma cells silenced for AMBRA1 and in *Ambra1* KO tumor-derived primary cells argues for a contribution of melanoma cells. In line with this, it is worth mentioning that a novel transcriptional function of Ambra1 in the nucleus has recently been reported^46^. Indeed, its association to chromatin and interaction with transcriptional factors may likely hint at a direct contribution of Ambra1 to the transcriptional reprogramming observed in *Ambra1*-deficient tumors.

Autophagy was suggested to have a role during melanoma development and progression^47,48^. However, we were not able to correlate either the pro-invasive state of *BPA*^*−/−*^ melanoma or the hyperactivation of FAK1 signaling to the AMBRA1 pro-autophagy functions^7^. In fact, while autophagy flux was affected in AMBRA1-silenced cells (Supplementary Fig. 6j, k), bulk autophagy was not altered in *Ambra1*-null *Braf*-mutated melanocytes (Supplementary Fig. 2f, j) and melanomas (Supplementary Fig. 6l, m), thus implying compensatory events taking place in the tumor.

On the other hand, we demonstrated that the pharmacological inhibition of FAK1 signaling was sufficient to revert the growth of *Ambra1*-deficient *Braf/Pten*-driven melanomas in vivo. Moreover, a reduced invasiveness as well as an increased sensitivity to *FAKi* was observed in null or low-expressing AMBRA1 melanoma cells treated with *FAKi* in vitro. Although this evidence should be validated in vivo through *FAK1* genetic ablation in order to circumvent possible off-target effects of pharmacological inhibition, concomitant silencing of FAK1 and AMBRA1 in vitro confirmed the reduced propensity of melanoma cells to migrate and invade. This discovery paves the way for a possible therapeutic application in *AMBRA1*-deficient melanomas of FAK1 inhibitors, which are already exploited in preclinical models of melanoma with acquired resistance to BRAFi^49^, and in clinical trials for solid tumors^42,50,51^. In support to this, we have also observed that *AMBRA1-*low-expressing human melanoma cell lines and tumors (patient data from the TCGA database and LMC cohort) exhibit upregulation of FAK1 signaling, ECM remodeling, EMT and invasiveness genes.

Taken together, our discoveries pinpoint AMBRA1 as tumor suppressor in melanoma, and suggest the potential use of FAK1 inhibitors in current melanoma therapy for AMBRA1-low tumors.

## Methods

### Study design

To determine sample size of the in vivo studies involving transgenic mice, a qualitative power analysis was performed using the formula:

1 $${\rm{sample}}\; {\rm{size}}=\frac{2{({Z}^{{\rm{\alpha }}/2}+{Z}^{{\rm{\beta }}})}^{2}\times P(1-P)}{{({p}_{1}-{p}_{2})}^{2}}$$

where *Z*^α/2 ^= type I error of 5% (=1.96, from *Z* table), *Z*^β ^= 0.842 at power 80% (from *Z* table), *p*_1 _– *p*_2_ = difference in proportion of events in two groups, *P* =  pooled prevalence, i.e., (prevalence in case group [*p*_1_] + prevalence in the control group [*p*_2_])/2. Sample size values were adjusted for 10% attrition. No randomization, blinding or gender distinctions were applied in this instance. We performed RNAseq analysis on murine tumors and staining of tumor sections, which were evaluated by a pathologist (F.M.B.) blinded to experimental conditions. All the in vitro experiments were repeated in at least triplicates, unless otherwise indicated, to ensure enough variation and statistical significance. We evaluated the causative role of FAK1 signaling hyperactivation to the phenomena observed upon AMBRA1 depletion first in vitro and then on tumor growth in vivo. To do so, we used syngeneic models for melanoma in which animals were treated with a FAK inhibitor when tumors were measurable. Sample size was calculated quantitatively using the formula:

2 $${\rm{sample}}\; {\rm{size}}=\frac{2{{\rm{SD}}}^{2}\times {({Z}^{{\rm{\alpha }}/2}+{Z}^{{\rm{\beta }}})}^{2}}{{d}^{2}}$$

where SD = standard deviation (from previous studies^52^, *Z*^α/2^ = type I error of 5% (=1.96, from *Z* table), *Z*^β^ = 0.842 at power 80% (from *Z* table), *d* = effect size. All sample size values were adjusted for 10% attrition. Randomization, gender restriction, but no blinding, were applied to the syngeneic models. For all the in vivo experiments, endpoints were established before experiments were performed according to the Danish regulations.

### Genetically engineered mouse models

All animal care and mice experiments were performed in compliance with institutional guidelines and with protocols approved by the Danish animal experiments inspectorate (Dyreforsøgstilsynet, 2015-15-0201-00586, 2020-15-0201-00578). All mice were maintained in pathogen-free, ventilated cages in the Animal facility at the Danish Cancer Society, and allowed free access to food and water in a 12 h light/dark cycle, with room temperature at 21–24 °C, humidity 55 ± 10%. All cages contained wood shavings, bedding, nest material and cardboard tube for environmental enrichment. All the mice used for the study are female and male, C57Bl/6N strain (RRID:MGI:5651595). *Ambra1*^*flox/flox*^, previously generated in our laboratory as described in Becher et al.^16^, were crossed with *Tyr::CreER*^*T2/+*^;*Braf*^*V600E/+*^*;Pten*^*flox/flox*^^15^. For the analysis of melanocytic lesions, breedings were set to generate *Tyr:CreER*^*T2/+*^*;Braf*^*V600E/+*^ background with different Ambra1 flox copy number herein referred to as *BA*^*+/+*^ (*Ambra1*^*+/+*^), *BA*^*+/−*^ (*Ambra1*^*+/−*^) or *BA*^*−/−*^ (*Ambra1*^*−/−*^). For the analysis of melanomas, breeding pairs were set to generate *Tyr:CreER*^*T2/+*^*;Braf*^*V600E/+*^*;Pten*^*−/−*^ background with different *Ambra1 flox* copy number herein referred to as *BPA*^*+/+*^ (*Ambra1*^*+/+*^), *BPA*^*+/−*^ (*Ambra1*^*+/−*^) or *BPA*^*−/−*^ (*Ambra1*^*−/−*^). Animals were maintained in an inbred background.

Melanocytic lesions in *BA*^*+/+*^, *BA*^*+/−*^ and *BA*^*−/−*^ mice were induced by topical application of 10 µl (50 mg/ml) of (Z)-4-Hydroxytamoxifen (4-OHT) (Sigma-Aldrich, MO, USA; cat# H6728) solution in dimethyl sulfoxide (DMSO) with a paintbrush onto the dorsal skin of both female and male pups at postnatal days 1, 3, and 5^53^. Local administration of 4-OHT in in *BPA*^*+/+*^, *BPA*^*+/−*^ and *BPA*^*−/−*^ mice was induced by local application of 1.5 µl (7.8 mg/ml) of 4-OHT in EtOH onto the shaved dorsal skin of 3-week-old mice^53^. Tumors were measured using electronic calipers, and volume (*V*) was calculated using the formula:

3 $$V=(L\times W\times H)\times \frac{{\rm{\pi }}}{6}$$

where *L* = Length; *W* = Width; *H* = Height. For the tumor growth curves, each data point corresponds to the average tumor volume in each experimental group for a given day after 4-OHT induction. Tumor weights were measured at endpoint. *BPA*^*+/+*^, *BPA*^*+/−*^, and *BPA*^*−/−*^ mice were euthanized at various time-points after 4-OHT application or when tumor reached maximum volume allowed by Danish legislation [(*L* + *W*)/2 ≥ 12] (maximum tolerated tumor).

### Determination of Cre-recombinase efficiency

DNA was extracted from 10 mg of bulk tumors from *BPA*^*+/+*^, *BPA*^*+/−*^ and *BPA*^*−/−*^ mice using the E.Z.N.A. Tissue DNA Kit (Omega Bio-Tek, GA, USA; cat# D3396/02). Briefly, samples were digested for 2 h at 55 °C with Proteinase K provided in the kit. DNA was then precipitated and bound to the column. After washing, DNA was eluted in 5 mM Tris-HCl. The efficiency of the Cre recombination was determined by PCR for *Braf* and *Pten*, as described in Dankort et al.^54^ and Yao et al.^55^, respectively. Efficiency of Ambra1 recombination was assessed by western blot (WB) analyses (see below).

### Tissue processing and paraffin sectioning

At the time of collection, mice tissue samples (perfused lungs, dorsal skin, inguinal lymph nodes or tumors) were collected and fixed overnight in PFA 4% at 4 °C. Samples were then washed three times in PBS, processed in an STP 120 Spin Tissue Processor (ThermoFisher Scientific, MA, USA; cat# 813150) overnight and embedded in paraffin (Hounisen, DK; cat# 2270.6060) using a Microm EC 350 modular tissue embedding center (ThermoFisher Scientific, MA, USA). Unless otherwise indicated, sections of 4 µm were cut using an HM325 rotary microtome (ThermoFisher Scientific, MA, USA; cat# 902100), MX35 Premier blades (ThermoFisher Scientific, MA, USA; cat# 3051835) and ThermoScientific™ SuperFrost Plus™ Adhesion slides (ThermoFisher Scientific, MA, USA; cat# J1800AMNZ). Sections were deparaffinized with xylene and graded ethanol, and rehydrated. Sections were then stained according to various staining procedures.

### Hematoxylin & eosin (H&E) staining

After deparaffinization and rehydration, tumors, lungs, lymph nodes or skin sections were stained with hematoxylin (Sigma-Aldrich, MO, USA; cat# GHS232) and eosin (Sigma-Aldrich, MO, USA; cat# HT110232) according to manufacturer’s instructions, dehydrated and mounted with xylene. All bright field images (skin, lymph nodes and tumor sections) were acquired using a Leica DMLS microscope (Leica, DE) coupled with a Leica ICC50 HD camera (Leica, DE) and Leica Application Suite v4.2.0, or using a NanoZoomer-XR Digital slide scanner (Hamamatsu, JP) and NDP.view 2 software (version 2.7).

### Picrosirius Red staining

After deparaffinization and rehydration, sections were stained with Weigert’s hematoxylin solution for 5 min. The solution was prepared following standard protocols with hematoxylin (Merck, NJ, USA; cat# 1.15938.0100), iron(III) chloride hexahydrate (Merck, NJ, USA; cat# 1.03943.0250) and hydrochloric acid (Merck, NJ, USA; cat# 1.00317.1000). After rinsing the sections with water for 10 min, the sections were stained for 1 h with Sirius Red 0.1% in Picric Acid Saturated (Ampliqon, DK; cat# AMPQ40522.1000), rinsed again with water for 1 min, dehydrated and mounted with xylene. The staining was assessed under polarized light with a Leika microscope by a pathologist (F.M.B.) blinded to the experimental conditions and a histopathological score assigned as follows: *0* for prevalence of fine fibrillar stroma, *1* for fibrillar stroma with occasional presence of isolated thick collagen fibers, *3* for presence of long and thick confluent collagen fibers.

### Immunohistochemistry (IHC) and immunofluorescence (IF) on tissue sections

Tissue sections were deparaffinized, rehydrated and washed in PBS Tween 0.05 % (PBS-T). Antigen retrieval was performed in Target Retrieval Solution, Citrate pH 6 (Dako, CA, USA; cat# S236984) (S100 IHC/IF for 4 min, p-c-Myc-S62 IHC/IF for 15 min, Cyclin D1 IHC/IF for 4 min, p-Fak-Y576 IF for 4 min, p62 IF for 4 min) or Tris-EDTA buffer pH 9.0 (Ki67 IHC/IF for 4 min) using a microwave. Endogenous peroxidases were blocked with 3% H_2_O_2_ in water for 10 min, sections were washed in PBS-T and blocked with Protein Block Serum-Free (Dako, CA, USA; cat# X0909) for 30 min at room temperature. Primary antibodies in antibody diluent (Dako, CA, USA; cat# S0809) were applied to the sections overnight at 4 °C. For IHC, stainings were visualized using EnVision^+^ (Dako, CA, USA; cat# K4003) or Histofine® One-Step Polymer Detection System (for p-c-Myc-S62; Nacalai, USA; cat# 414311F) and DAB^+^ (Dako, CA, USA; cat# K3468). Sections were counterstained with hematoxylin (Dako, CA, USA; cat# S3309) according to manufacturer’s instructions, dehydrated and mounted with xylene. Digital images were acquired with a NanoZoomer-XR Digital slide scanner (Hamamatsu, JP) and NDP.view 2 software (version 2.7). For IF, incubation of the primary antibody was followed by incubation with a Goat anti-Rabbit IgG (H+L) Cyanine5 (1:400, ThermoFisher Scientific, MA, USA; cat# A-10523; RRID: AB_2534032) or with a Goat anti-Rabbit IgG (H+L) Alexa Fluor 568 (1:400, ThermoFisher Scientific, MA, USA; cat# A-11011; RRID:AB_143157) for 2 h at room temperature. Nuclear staining was performed for 10 min at room temperature with 1 µg/ml Hoechst 33342 (ThermoFisher Scientific, MA, USA; cat# H3570). Sections were mounted with fluorescence mounting medium (Dako, CA, USA; cat# S3023). The primary antibodies used for both IHC and IF are as follows: anti-S100 (ready-to-use, Dako, CA, USA; cat# IS504; RRID:AB_2811056), anti-Ki67 (IHC 1:1000, IF 1:250, Abcam, UK; cat# ab16667; RRID:AB_302459), p-c-Myc-S62 (1:150, antibody developed and validated by the Prof. Sears laboratory^56^), Cyclin D1 (IHC 1:400, IF 1:500, Abcam; cat# ab16663), p-Fak-Y576 (IF 1:200, ThermoFisher Scientific, MA, USA; cat# PA5-104964), p62 (IF 1:500, MBL, MA, USA; cat# PM045). All the IHC and Breslow thickness of tumors were assessed by a pathologist (F.M.B.) blinded to the experimental conditions. IF were imaged using a fluorescent AxIo Imager.A2 microscope (Carl Zeiss AG, DE) equipped with an AxioCaM HRM (Carl Zeiss AG, DE) and provided with a ZEISS ZEN microscope software v2.3 (Carl Zeiss AG, DE; RRID:SCR_018163).

Morphologically, melanomas produced with this model were composed of three different components, with peculiar melanocytic populations and features of the stroma. The most superficial component was composed of heavily pigmented melanocytes arranged in nests, positive for S100 in the cytoplasmic compartment, with scarce stroma between the cells (“pigmented population”). The deeper part of the melanoma was divided between two coexisting melanocytic populations. The first of these, with pleomorphic nuclei and stronger cytoplasmic S100 positivity, with loss of the pigmentation that characterized the superficial component but retained growth in nests with little admixed stroma (“S100^+^ population”). The last, characterized by smaller, monomorfic cells with weaker or lost S100 expression and separated by a loose, myxoid matrix. The QuPath software (RRID:SCR_018257) version v0.1.2 was used to calculate the amount of these three morphological categories, the percentage of Ki67-positive cells in a given area of the section, the intensity of signal of p-c-Myc-S62 and of Cyclin D1 in IHC analyses, the nuclear or cytosolic IF intensity of the signal of Cyclin D1 and p-c-Myc-S62 and of p-Fak-Y576 and p62 (respectively) and the number/area of tumor masses in the lungs. To assess the presence of micrometastases in the lymph nodes, both inguinal lymph nodes were collected from mice from both sexes 42 days after 4-OHT induction, fixed, embedded in paraffin, stained with anti-S100 and analyzed with ImageJ version 1.52.n (University of Wisconsin, WI, USA; RRID:SCR_003070). For each lymph node, three sections were quantified and the average percentage of S100-positive area per lymph node was calculated. The values obtained for the lymph nodes of the same mouse were summed in order to obtain a single data point per mouse. Five mice were analyzed in each group and each data point corresponds to the average % of S100-positive area. Area of iLNs was calculated using ImageJ version 1.52.n (University of Wisconsin, WI, USA; RRID:SCR_003070). For each mouse both iLNs were counted. Data are expressed as average ± SEM.

### Melanocytic nevi quantification

Paraffin-embedded skin sections from both female and male mice samples collected at 13 weeks after 4-OHT application were stained with H&E and analyzed with ImageJ version 1.52.n (University of Wisconsin, WI, USA; RRID:SCR_003070). For each section, the hypodermal area of the specimen was defined. This area corresponded to the hypodermal layer of the skin where most melanocytic nevi were located. Within the hypodermal area, a threshold was set to detect the pigmented area. Each data point represents one mouse and corresponds to the average percentage of the pigmented area out of the total hypodermal area for that mouse (at least four sections were analyzed for each mouse to ensure variability).

### TUNEL assay

To assess cell death, the TUNEL Assay Kit-BrdU-Red (Abcam, UK; cat# ab66110) was used following manufacturer’s instructions. Tissue sections were deparaffinized, rehydrated and treated with 20 µg/ml Proteinase K (ThermoFischer Scientific, MA, USA; cat# AM2548) for 5 min according to the kit’s protocol. Nuclear staining was performed for 10 min at room temperature with 1 µg/ml Hoechst 33342. Images were taken using a using a fluorescent AxIo Imager.A2 microscope (Carl Zeiss AG, DE) equipped with an AxioCaM HRM (Carl Zeiss AG, DE) and provided with a ZEISS ZEN microscope software v2.3 (Carl Zeiss AG, DE).

### Nuclei count

Tissue sections were deparaffinized, rehydrated and nuclei stained for 10 min at room temperature with 1 µg/ml Hoechst 33342. Nuclei were imaged using a fluorescent AxIo Imager.A2 microscope (Carl Zeiss AG, DE) equipped with an AxioCaM HRM (Carl Zeiss AG, DE) and provided with a ZEISS ZEN microscope software v2.3 (Carl Zeiss AG, DE). At least four different fields were analyzed for each sample and the number of nuclei determined with ImageJ version 1.52.n (University of Wisconsin, WI, USA; RRID:SCR_003070).

### Primary melanoma cells isolation

The procedure herein described was carried out in sterile conditions in a tissue culture hood. Tumors were excised from animals (both genders), weighted and the skin carefully removed with a rounded blade scalpel. Tumors were washed in 70% EtOH for 10 s, followed by two quick washes in sterile PBS (ThermoFisher Scientific, MA, USA; cat# 14190-094) supplemented with 100 U/ml P/s. Tumors were mechanically dissociated in 1–2 mm^3^ fragments with a rounded blade scalpel and digested in freshly prepared digestion buffer (RPMI 1640 Medium supplemented with 3 mg/ml Collagenase A –Roche, CH; cat# 10103586001, and 1.5 mg/ml porcine pancreatic trypsin –Sigma-Aldrich, MO, USA; cat# T1426) (5:1, v/w) upon gentle rotation (20 rpm) for 1 h at room temperature (RT). After digestion, the suspension was further dissociated with a serological pipette and non-digested tissue debris removed by centrifugation at 800 × *g* for 10 min using a 70 µm Falcon^®^ Cell Strainer (Corning, NY, USA; cat# 352350). Supernatant was removed and cell pellets washed twice with RPMI 1640. Viability was determined by Trypan Blue exclusion test and cells were kept in culture in RPMI 1640 supplemented with 100 U/ml P/s and 2% fetal bovine serum (FBS) for the first 5–7 days, to negatively select fibroblasts, and eventually in RPMI 1640 supplemented with 20% FBS. *BPA*^*+/+*^*-* or *BPA*^*−/−*^-derived melanoma primary cells (Bdmc^+/+^ and Bdmc^−/−^, respectively) were kept in culture no longer than 3 weeks at 37 °C in an atmosphere of 5% CO_2_ in air. Growth medium was replaced every 48 h and a subcultivation ratio of 1:3 used every 3–4 days.

### Intravenous tail injection and melanoma cells homing

Tumor pieces from *BPA*^*+/+*^ or *BPA*^*−/−*^ mice were mechanically dissociated in 1–2 mm^3^ fragments with dissection scissors in two separate falcon tubes (tumor pieces with the same genotype were pooled to obtain a single cell suspension for each genotype) and digested in 2 ml freshly prepared sterile-filtered digestion buffer (RPMI 1640 Medium supplemented with 2.1 mg/ml Collagenase type I, 75 µg/ml DNAse I, 5 mM CaCl_2_ and 1% Penicillin-Streptomycin, P/s –ThermoFisher Scientific, MA, USA; cat# 15140-122) upon gentle rotation overnight at 4 °C. Tumor samples were then incubated at 37 °C for 1 h. The suspension was further dissociated with a serological pipette and non-digested tissue debris removed by centrifugation at 800 × *g* for 10 min using a 70 µm Falcon^®^ Cell Strainer (Corning, NY, USA; cat# 352350). Viability was determined by Trypan Blue exclusion test and cells were resuspended in an appropriate amount of isotonic solution. Ten to 15-week-old C57Bl/6N (RRID:MGI:5651595) females were acclimated for 1 week in the facility and randomly injected with 1.5 × 10^5^ *BPA*^*+/+*^*-* or *BPA*^*−/−*^-derived tumor cells in the tail vein to assess homing of melanoma cells to the lungs. Mice were monitored daily and were euthanized 3 months after tail vein injection. At the time of collection, lungs were perfused with formaldehyde 4% (PFA 4%) aqueous solution (VWR, PA, USA; cat# 9713.1000) and processed for histological analysis. Lungs were blindly analyzed by a pathologist (F.M.B.) for quantification of tumor masses and size.

### Subcutaneous injections and FAKi treatment

Six to 8 and 11–12-week-old C57Bl/6N (RRID:MGI:5651595) female mice were purchased from Taconic Biosciences A/S and allowed to acclimate in the facility for 1–2 weeks before randomization. At the day of injection, Bdmc^+/+^ and Bdmc^-/-^ cells were washed in PBS, detached by trypsin and counted. Either 7 × 10^5^, 2 × 10^6^ (in 6–8-week-old mice) or 5 × 10^6^ (in 11–12-week-old mice) cells resuspended in 100 µl of saline solution supplemented with 1 mM EDTA were injected in the right flank of each mouse (*sBPA*^*+/+*^: mice injected with Bdmc^+/+^ cells; *sBPA*^*−/−*^: mice injected with Bdmc^−/−^ cells). Mice were monitored over a long period of time to assess tumor appearance (Tumor-free mice). When the tumors of the mice injected with 2 × 10^6^ and 5 × 10^6^ cells were measurable (ca. 30 mm^3^), mice were randomly divided in subgroups and received every day for 21 and 10 days, respectively, an IP injection of either 20 mg/kg FAK inhibitor 14 (*FAKi*; Y15, Sigma-Aldrich, MO, USA, cat# SML0837) dissolved in saline solution or saline solution, as control (*Vehicle*). Mice were monitored/observed on daily basis and weight determined twice a week to assess health status upon *FAKi* administration. Tumor volumes were measured twice a week or daily, respectively, by a digital caliper and mice euthanized, respectively, after 21 or 10 days from the first IP. Tumor volumes (*V*) were determined using the formula:

4 $$V=\frac{(W\times {L}^{2})}{2}$$

where *W* > *L* and *W* = width and *L* = length, and are expressed as fold-change with respect to the first day of treatment.

### Cell lines and treatments

Human melanoma cell lines MeWo (ATCC^®^ HTB-65^™^; RRID: CVCL_0445), SK-Mel-2 (ATCC^®^ HTB-68^™^; RRID: CVCL_0069) and SK-Mel-5 (ATCC^®^ HTB-70^™^; RRID: CVCL_0527) were purchased from ATCC^®^ (Manassas, VA, USA) and cultivated in Advanced Minimum Essential Media (MEM) (ThermoFisher Scientific, MA, USA; cat# 12492-021) supplemented with 2 mM GlutaMAX™ (ThermoFisher Scientific, MA, USA; cat# 35050-038). European Searchable Tumor Line Database (ESTDAB, https://www.ebi.ac.uk/ipd/estdab; RRID:SCR_007746) cell lines were cultivated in RPMI 1640 Medium (ThermoFisher Scientific, MA, USA; cat# 61870-010). All culture media were supplemented with 10% FBS (ThermoFisher Scientific, MA, USA; cat# 10270-106) and 100 U/ml P/s. Cells were cultured in an atmosphere of 5% CO_2_ in air at 37 °C and passaged no >18 times before a low-passage cryovial was thawed. Cells were expanded, cryopreserved and used within few months of resuscitation. All the cell lines were routinely tested for *Mycoplasma* contamination at thawing, during subculture and prior to cryopreservation using a PCR-based detection method (eurofins Genomics, DE). During the experiments, cells were plated at a density of 2 × 10^5^ cells/ml, unless otherwise indicated. Chloroquine (CQ) was dissolved in DMSO and used at a final concentration of 40 µM for 2 h, while DMSO was used in control cells. *FAKi* was used at a final concentration of 1 µM in Bdmc cells and at 0.004 µM, 0.02 µM, 0.1 µM, 0.5 µM, and 2.5 µM final concentrations in the ESTDAB cells, while PBS was used in control cells (*Vehicle*).

### siRNAs and transfection methods

siRNA transfection was performed at time of seeding (reverse transfection) using a 20 nM concentration for a total of 48 h, unless otherwise indicated. Custom-designed siRNA sequences for AMBRA1 are as follows: *siAMBRA1 (#1)*: *5’-* GGC CUA UGG UAC UAA CAA A *-3’*; *siAMBRA1 5‘-UTR (siAMBRA #2): 5’- GGA CAA CUU ACA AGG ACC -3’*. Unless otherwise indicated, *siAMBRA1* refers to *siAMBRA1 #1*. Custom-designed siRNA sequence for ATG7 is: *siATG7*: *5’-* CAG UGG AUC UAA AUC UCA AAC UGA U *-3’*. Silencing of FAK1 was obtained using either the MISSION^®^ esiRNA human PTK2 (*siFAK1 #1*, Sigma-Aldrich, MO, USA; cat# EHU077321) or the custom-designed siRNA (*siFAK1 #2*) *5’-* AAC CUC GCA GUC AUU UAU CAU *-3’*. Unless otherwise indicated, *siFAK1* refers to *siFAK1 #1*. Negative control cells (*siScr*) were transfected with the MISSION^®^ siRNA Universal Negative Control #1 (Sigma-Aldrich, MO, USA; cat# SIC001). The DNA constructs coding for AMBRA1, myc-flagged-AMBRA1 and HA-tagged-FAK1 were cloned in the pcDNA™3.1 Mammalian Expression Vector (ThermoFisher Scientific, MA, USA; cat# V79020). The coding sequences were amplified by PCR and cloned in the acceptor vector by means of EcoRI and NotI restriction sites. HA-FAK1 plasmid construct was mutagenized (using FAK1 as template and custom-designed primers) in the same region (exon 11) targeted by *siFAK1 #2* in order to make it *siFAK1 #2*-resistant (HA-FAK1.miRes). The AMBRA1^*PXP*^ and the HA-FAK1.miRes^*P876A/P882A*^ (HA-FAK1.miRes^*AA*^) mutants were obtained by site-directed mutagenesis using AMBRA1 and FAK1 as template, respectively, and custom-designed primers. The overexpression of AMBRA1 and FAK1 plasmid constructs was carried out for 24 h, 24 h after cells had been reversely transfected with the *siAMBRA1-5’UTR* and the *siFAK1 #2* in order to knock-down endogenous AMBRA1 and FAK1 levels, respectively. ß-Gal-myc- and HA-expressing plasmids were used as negative controls. All transfections were performed using Lipofectamine™ 2000 Transfection Reagent (ThermoFisher Scientific, MA, USA; cat# 11668-019), according to the instructions.

### Immunoprecipitation

Immunoprecipitation was performed in HEMG Buffer (25 mM Hepes pH 8.0, 12.5 mM MgCl_2_, 0.1 mM EDTA pH 8.0, 10% Glycerol, 100 mM NaCl, 0.5% Triton X-100). Cells were lysed in HEMG buffer completed with phosphatase (1 mM Sodium fluoride and 1 mM Sodium orthovanadate, VWR) and protease (Protease Inhibitors Cocktail, Sigma-Aldrich) inhibitors. After clarification (10,000 × *g* for 15 min) protein extracts were quantified by DC Protein Assay (BIO-RAD) and 0.5–1.5 mg of protein extracts for each condition pre-cleared by 30 min incubation with 0.2 mg of protein G magnetic beads (Dynabeads^TM^, Invitrogen). Next, the pre-cleared lysates were incubated overnight with 0.4 mg of protein G magnetic beads and 1 µg of primary antibodies (mouse immunoglobulins for IP Ctrl, anti-HA (Sigma-Aldrich, cat# H3663) and anti-AMBRA1 (Santa Cruz Biotechnology, cat # sc-398204)). Purified complexes were washed three to four times and denatured with Laemmli Sample Buffer 4X (NuPAGE^TM^ LDS Sample Buffer 4X plus NuPAGE^TM^ Sample Redung Agent 10X, Invitrogen) and boiled at 95 °C for 5 min. Protein complexes were then analyzed by western blot.

### Protein expression analysis

Total protein lysates were obtained by resuspending cell pellets in RIPA Buffer (50 mM Tris-HCl pH 7.5, 150 mM NaCl, 1 mM EDTA, 5 mM MgCl_2_, 1% Triton X-100, 0.25% Sodium Deoxycholate, 0.1% SDS, 5 mM β-glycerophosphate, 5 mM NaF, 2 mM Na_3_VO_4_, 1:100 protease inhibitor cocktail) on ice for 20 min. Mice tumors were disrupted in RIPA Buffer (30:1, v/w), after the skin was carefully removed, using a Qiagen Tissuelyser II (Qiagen, DE) (frequency 20/s, 2 min) with stainless steel beads. All lysates were sonicated (three cycles, 5 s ON/OFF) and centrifuged at 15,000 × *g* for 10 min at 4 °C. Protein extracts from the ESTDAB cells #002, #033, #039, #043, #047, #067, #095, #097, #114, #115, #128, #129, #135, #160, #161, #162, #199 were obtained using the AllPrep DNA/RNA/Protein Mini Kit (Qiagen, DE; cat# 80004) according to the instructions. Protein concentration was determined according to the Lowry’s method and protein extracts separated by sodium dodecyl sulphate–polyacrylamide gel electrophoresis using Criterion™ TGX™ Precast Gels (Bio-Rad Laboratories, CA, USA) and blotted on PVDF using a Trans-Blot^®^ Turbo™ Transfer System (Bio-Rad Laboratories, CA, USA). Primary antibodies used can be found in the Supplementary Table 2 of the Supplementary Information file. Images were taken using a ChemiDoc™ MP System (Bio-Rad Laboratories, CA, USA; cat# 1708-280) provided with the Image Lab 6.0.1 Software (Bio-Rad Laboratories, CA, USA). Densitometry analyses were performed using ImageJ version 1.52.n (University of Wisconsin, WI, USA; RRID:SCR_003070). All uncropped blots can be found in Source Data.

### RNA isolation

For RT-qPCR analyses, total RNA was isolated directly from culture cells using the NucleoSpin™ RNA Columns (Macherey-Nagel™, DE; cat# 740955) according to the instructions of the manufacturer. Mice tissues were disrupted using a Qiagen Tissuelyser II (as previously described) after the skin was accurately removed. Total RNA from the ESTDAB cells #002, #033, #039, #043, #047, #067, #095, #097, #114, #115, #128, #129, #135, #160, #161, #162, #199 was obtained using the AllPrep DNA/RNA/Protein Mini Kit (Qiagen, DE; cat# 80004) according to the instructions. For RNA-seq analyses, RNA from tissues (disrupted with Qiagen Tissuelyser II) were isolated using the RNeasy Plus Mini Kit (Qiagen, DE; cat# 74134), following the instructions of the manufacturer. When RNA from tissues could not be isolated at the moment, tumor pieces were preserved in RNA*later*™ Stabilization Solution (ThermoFisher Scientific, MA, USA; cat# AM7020), according to the instructions.

### Reverse transcription and quantitative RT-PCR

Reverse transcription was performed for 1 h at 37 °C with 500 ng of total RNA with the M-MLV reverse transcriptase (Promega, WI, USA; cat# M1705) and diluted 3x before any RT-qPCR analysis. mRNA expression levels were measured using the PowerUp™ SYBR™ Green Master Mix (ThermoFisher Scientific, MA, USA; cat# A25742), according to the instructions, on a ViiA 7 Real-Time PCR System v1.3 (Applied Biosystems, CA, USA). Fold changes in mRNA levels relative to the control were calculated after normalizing to an internal housekeeping (*L34*). All reactions were run as triplicates. The specific primer pairs were custom designed and tested with Primer-BLAST (NCBI; RRID:SCR_003095). Primers used were obtained from TAG Copenhagen A/S (Copenhagen, DK) and are listed in the Supplementary Table 3 of the Supplementary Information file.

### RNA sequencing and analysis

RNA sequencing was performed on bulk tumor extracts from *BPA*^*+/+*^ and *BPA*^*−/−*^ mice by Genomix4Life Srl (Baronissi, SA, IT). Samples were sequenced with Illumina NextSeq in paired-end mode with a read length of 76 bp. Quality control of the reads and base-calling was performed with FastQC v0.11.9 (Babraham Institute, UK; RRID:SCR_014583). Removal of adapter and very short reads was achieved with cutadapt v1.18 (TU Dortmund University, DE; RRID:SCR_011841). Reads were aligned to *Mus Musculus* (mm10) using the STAR software version 2.5.3a (Cold Spring Harbor Laboratory, NY, USA; RRID:SCR_015899) with standard parameters for paired-reads. Transcriptome expression was quantified with featureCount Release 1.6.3 (Walter and Eliza Hall Institute of Medical Research, AU; RRID:SCR_012919). The reference track was the assembly mm10 obtained from Ensembl (RRID:SCR_002344) using iGenomes (Illumina, CA, USA). Differential expression analysis was performed with R (R project for Statistical Computing, version 3.6.0; RRID:SCR_001905) Bioconductor (RRID:SCR_006442) package DESeq2 version 1.4.02 (RRID:SCR_015687), pathway analysis was performed with the online tool EnrichR (Icahn School of Medicine at Mount Sinai, NY, USA; RRID:SCR_001575) and gene set enrichment analysis was performed with the GSEA java desktop application for Mac version 4.0.3 (Broad Institute, MA, USA; RRID:SCR_003199). A detailed description of the parameters used for each analysis and the processing of the RNAseq data can be found in the Supplementary Data 2.

### In silico analyses using publicly available datasets

RNAseq data for skin cutaneous melanoma (SKCM) samples was downloaded from The Cancer Genome Atlas (TCGA; RRID:SCR_003193) and pre-processed with the TCGAbiolinks R package 2.18.0 (RRID:SCR_017683). RNAseq (RPKM values) data for melanoma cell lines was downloaded directly from The Cancer Cell Line Encyclopedia (CCLE) website (portals.broadinstitute.org/ccle; RRID:SCR_013836). Raw mRNA expression data for Leeds Melanoma Cohort was downloaded from European Genome Archive using the accession number EGAS00001002922. Raw data was normalized using R package *LUMI*^57^ (version 2.38.0) followed by quantile normalization and log2 transformation. Probe set identifiers were mapped to official gene symbols using annotation package for Illumina’s DASL-HT12-v4 array. Melanoma patient samples or cell lines were classified in AMBRA1_Low or AMBRA1_High groups according to their AMBRA1 mRNA expression levels. Arbitrary cut-offs were chosen to define those groups. For all three datasets, the top 15% of the population in terms of AMBRA1 mRNA expression defined the AMBRA1_High group while the bottom 15% defined the AMBRA1_Low group. As a result, for the (i) TCGA-SKCM data, both groups contained *n* = 70 patients; (ii) CCLE data, both groups contained *n* = 7 cell lines; and (iii) Leeds Melanoma Cohort, both groups contained *n* = 105 samples. A detailed description of the parameters used for each analysis and the processing of the public data can be found in the Supplementary Data 2.

### Extracellular matrix digestion assay

Twenty-four hours after transfection, cells were washed, detached and counted. Five thousand cells were plated in 8-well Nunc™ Lab-Tek™ II Chamber Slide™ System (ThermoFisher Scientific, MA, USA; cat# 154534PK) coated with red-fluorescent Cy3-Gelatin, just before use. Cy3-Gelatin coating was performed according to the instruction of the QCM™ Gelatin Invadopodia Assay (Red) kit (Merck-Millipore, MA, USA; cat# ECM671). Twenty-four hours after seeding, medium was removed, cells washed with cold PBS and fixation performed with a 4% formaldehyde solution in PBS for 30 min at room temperature. Cells were rinsed twice with PBS and F-Actin and nuclei, respectively, stained with 2 µg/mL FITC-phalloidin and 1 µg/mL Hoechst 33342 in a solution of 2% BSA/0.25% Triton X-100 in PBS for 1 h at room temperature. Cells and areas of matrix degradation were digitalized using a fluorescent AxIo Imager.A2 microscope (Carl Zeiss AG, DE) equipped with an AxioCaM HRM (Carl Zeiss AG, DE) and provided with a ZEISS ZEN microscope software v2.3 (Carl Zeiss AG, DE) using a x40 magnification. For each sample, five different fields containing at least three to four cells were acquired and used for following quantifications. Quantifications were performed using the ImageJ version 1.52.n (University of Wisconsin, WI, USA; RRID:SCR_003070). FITC-phalloidin and Cy3-gelatin signals were, respectively, thresholded for high and low intensity to measure cell area and matrix degradation area. The same threshold was used for all the images. Data are expressed as relative matrix degradation area per cell, i.e., the ratio of the area of gelatin degradation on cell area.

### Transwell invasion assay

Ten-thousand cells from monoculture were seeded onto Nunc™ Polycarbonate Cell Culture Inserts in Multi-Well Plates (ThermoFisher Scientific, MA, USA; cat# 140629) in FBS-free growth medium 24 h after transfection or at the moment of seeding (ESTDAB and primary cells). Growth medium supplemented with 10% FBS was used in the bottom chamber as attractant. Cells were incubated at 37 °C for 24 h and then fixed and stained with a 0.05% (w/v) Crystal Violet solution in 20% MeOH. Cells on the top section of the insert were gently removed with a cotton swab. Four different fields were imaged for each sample with an IX71 inverted microscope (Olympus, Tokyo, JA) provided with a CellSens Imaging Software 2 (Olympus, Tokyo, JA; RRID:SCR_016238) and counted with ImageJ version 1.52.n (University of Wisconsin, WI, USA; RRID:SCR_003070). Data are showed as average count.

### Wound-healing assay

Twenty-four hours after transfection or plating, cells were seeded in 2-well silicone inserts with a defined cell-free gap of 500 µm (ibidi^®^, DE, cat# 80209) at high confluence in 6-well plates. The following day, the inserts were removed, cells washed twice with sterile PBS and complete growth medium supplied. Wound closure was followed over time, at the indicated times. Migrating cells were imaged with an IX71 inverted microscope (Olympus, Tokyo, JA) provided with a CellSens Imaging Software 2 (Olympus, Tokyo, JA; RRID:SCR_016238). The area of wound closure was calculated using the ImageJ version 1.52.n (University of Wisconsin, WI, USA; RRID:SCR_003070) with respect to the initial area (*T*_0_) and expressed as percentage of wound healing at the time-points indicated.

### Immunofluorescence analysis of focal adhesions

Twenty-four hours after transfection, cells were detached, diluted 1:3 and seeded on glass cover slips. The next day, growth medium was removed and cells washed twice in cold PBS and fixed with 4% paraformaldehyde (VWR International, Radnor, PA, USA) in PBS for 10 min at room temperature. Cover slips were incubated with a permeabilization solution (PBS/Triton X-100 0.2%, v/v), blocked for 1 h with a blocking solution (PBS/normal goat serum 5%, v/v, FBA 1%, v/v) and then incubated over night at 4 °C with anti-phospho-FAK Y397 (pFAK-Y397) (Cell Signaling #3283, 1:200; RRID:AB_10891442) and anti-Vinculin (Sigma-Aldrich, V9131, 1:500; RRID:AB_477629). Cells were then washed with cold PBS and incubated for 1 h with fluorophore-conjugated secondary antibodies (respectively AlexaFluor 568, RRID:AB_143157, and 647, RRID:AB_2535814). Nuclei were stained with 1 μg/ml Hoechst 33342 (Thermo Fisher Scientific, 62249, 1:1000) and F-actin was stained with fluorescein phalloidin (Thermo Fisher Scientific, F432, 1:100) for 30 min at room temperature. Confocal microscopy images were acquired by a LSM800 microscope (ZEISS; RRID:SCR_015963) equipped with an oil-immersion 63X objective and ZEN lite 2012 software Service Pack 2 (RRID:SCR_018163). Fluorescence images were deconvoluted using the software Huygens Professional engine 20.10.0p2 (Scientific Volume Imaging B.V.; RRID:SCR_014237) and adjusted for brightness, contrast and color balance by using Fiji (RRID:SCR_002285) analysis software (based on ImageJ v- 2.1.0/1.53c). The images represent projections and were achieved by summing the fluorescence signal of central *z*-stacks (two planes, 0.3 µm). The analysis of pFAK1-containing focal adhesions was performed by a modified version of the protocols previously published^25,58^ using Fiji imaging software. Briefly, the two central *z*-stacks of confocal images were summed, then pFAK-Y397 and Vinculin signals were merged in order to determine pFAK-Y397 containing focal adhesions. Pictures were thresholded using MaxEntropy algorithm, and particles with a size >0.1 µm^2^ were analyzed. Nuclear/perinuclear areas were excluded from the analysis. Area, perimeter, maximum Feret’s diameter of each pFAK-Y397-containing focal adhesion was analyzed and shape factor was calculated using the following formula:

5 $${\rm{Shape}}\; {\rm{factor}}=\frac{4{\rm{\pi }}\,\left({\rm{area}}\right)}{{\left({\rm{perimeter}}\right)}^{2}}$$

### Cell viability upon FAKi treatment

For each cell line (ESTDAB and Bdmc), 50,000 cells were plated in each well. After 24 h, medium was removed and replaced with fresh medium containing *FAKi* at different concentrations (0.004 µM, 0.02 µM, 0.1 µM, 0.5 µM and 2.5 µM, prepared as serial dilutions) for additional 24 h. Cell viability was assessed using the Cell Counting Kit-8 (Dojindo cat# CK04-11) at 450 nm using a VICTOR Multilabel Plate Reader (PerkinElmer, MA, USA) after 2 h of incubation, according to the manufacturer’s instructions. Data are shown as percentage of viable cells with respect to untreated cells (PBS, *Vehicle*). For ESTDAB cells, IC_50_ values were extrapolated using GraphPad/Prism8 (RRID:SCR_002798).

### Statistical analysis

For two-group comparison, two-tailed unpaired *t*-test or Wilcoxon rank-sum test (Mann–Whitney) was performed. For the Kaplan–Meyer survival curves, significance was assessed using the Log-rank (Mantel–Cox) test. Two-way-ANOVA was used for multiple comparisons in the wound-healing assay, the Crystal Violet assay and the tumor growth curves analysis (only the *p*-values at day 42 are shown in the graph). To determine correlation between AMBRA1 and EMT genes mRNA levels in ESTDAB cells, the Pearson correlation test was used. GraphPad/Prism8 (RRID:SCR_002798) was used for plotting graphs and to perform statistical analysis. Data are presented as means ± SEM or SD and significance was designated as follows: *p ≤ 0.05; **p ≤ 0.01; ***p ≤ 0.001; ****p ≤ 0.0001; *ns*, not significant.

All figures were assembled using Adobe Illustrator CC, 2020.

### Reporting summary

Further information on research design is available in the Nature Research Reporting Summary linked to this article.

## Supplementary information

Supplementary Information

Peer Review File

Description of Additional Supplementary Files

Supplementary Data 1

Supplementary Data 2

Reporting Summary

## Data Availability

The RNAseq datasets from the TCGA-SKCM analyzed during the current study are available on The Cancer Genome Atlas (TCGA, http://cancergenome.nih.gov/; RRID:SCR_003193). The RNAseq data from the CCLE melanoma cell lines analyzed during the current study are available on CCLE website (portals.broadinstitute.org/ccle). The transcriptomic data from the LMC analyzed during the current study were generated by the University of Leeds^29^ in connection with the project “The Leeds Melanoma Cohort”; otherwise known as Melanoma Follow-up and Case-Control Family Study (REC reference number 01/03/057). These data are available within the European Genome-phenome Archive at the European Bioinformatics Institute (accession number EGAS00001002922). Julia Newton-Bishop (j.a.newton-bishop@leeds.ac.uk) is the person to contact to access the EGA data. The RNAseq datasets generated from BPA^+/+^ and BPA^−/−^ samples during the current study are publicly available in GEO repository (accession number GSE151134). The authors declare that all other data supporting the findings of this study are available within the paper and its supplementary information files. Source data for Figs. 1–6, Supplementary Figs. 2–8 are provided with the paper as a separate “Source data” folder, which includes an Excel file with raw data for each figure in separate sheets, as well as a Pdf file with the uncropped versions of any gels or blots presented in this study. Source data are provided with this paper.

## Source data

Source Data
