# Supplementary material for: Loss of Ambra1 promotes melanoma growth and invasion
Source: Nat Commun. 2021 May 5;12:2550. doi: 10.1038/s41467-021-22772-2 (PMC8100102; doi:10.1038/s41467-021-22772-2)
Supplement: Supplementary file 1 — Supplementary Information [file 41467_2021_22772_MOESM1_ESM.pdf]

Loss of Ambra1 promotes melanoma growth and invasion

Di Leo *et al.*

Supplementary Information

Supplementary Figure 1

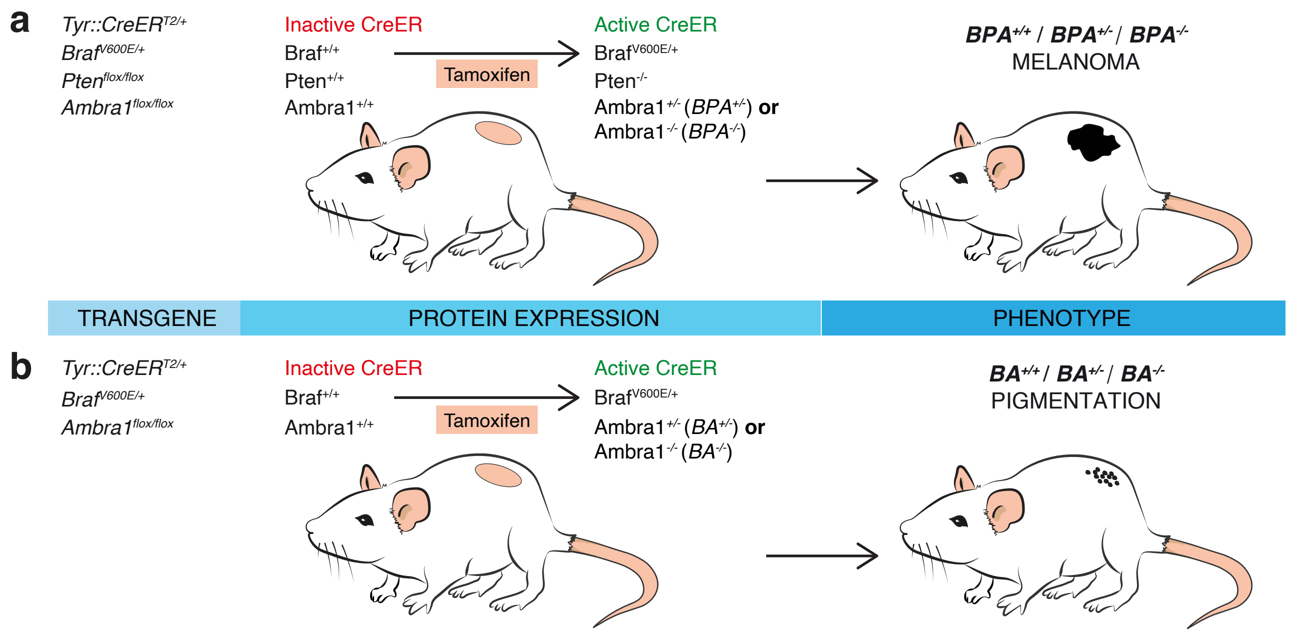

Supplementary Figure 1. GEMM models for melanoma and pigmentation. (a) Schematic representation of the GEMM used for melanoma induction. *Tyr::CreER*$^{T2/+}$*;Braf*$^{V600E/+}$*;Pten*$^{-/-}$*;Ambra1*$^{+/+}$ (*BPA*$^{+/+}$), *Tyr::CreER*$^{T2/+}$*;Braf*$^{V600E/+}$*;Pten*$^{-/-}$*;Ambra1*$^{+/-}$ (*BPA*$^{+/-}$) and *Tyr::CreER*$^{T2/+}$*;Braf*$^{V600E/+}$*;Pten*$^{-/-}$*;Ambra1*$^{-/-}$ (*BPA*$^{-/-}$) mice were administered 4-OHT topically at the age of 3 weeks on the skin of the lower back to initiate melanoma. 4-OHT-induced nuclear translocation of the CreER recombinase results in expression of mutated *Braf* (*Braf*$^{V600E/+}$), *Pten* deletion (*Pten*$^{-/-}$) and either single- (*Ambra1*$^{+/-}$) or double-allele deletion (*Ambra1*$^{-/-}$) of *Ambra1*. **(b)** Schematic representation of the GEMM used for melanocytic lesion formation. *Tyr::CreER*$^{T2/+}$*;Braf*$^{V600E/+}$*;Ambra1*$^{+/+}$ (*BA*$^{+/+}$), *Tyr::CreER*$^{T2/+}$*;Braf*$^{V600E/+}$*; Ambra1*$^{-/-}$ (*BA*$^{+/-}$) and *Tyr::CreER*$^{T2/+}$*;Braf*$^{V600E/+}$*; Ambra1*$^{-/-}$ (*BA*$^{-/-}$) mice were administered 4-OHT topically at postnatal days 1, 3 and 5 on the dorsal skin. 4-OHT-induced nuclear relocation of the CreER recombinase results in expression of mutated *Braf* (*Braf*$^{V600E/+}$) and either single- (*Ambra1*$^{+/-}$) or double-allele deletion (*Ambra1*$^{-/-}$) of *Ambra1.*

Supplementary Figure 2

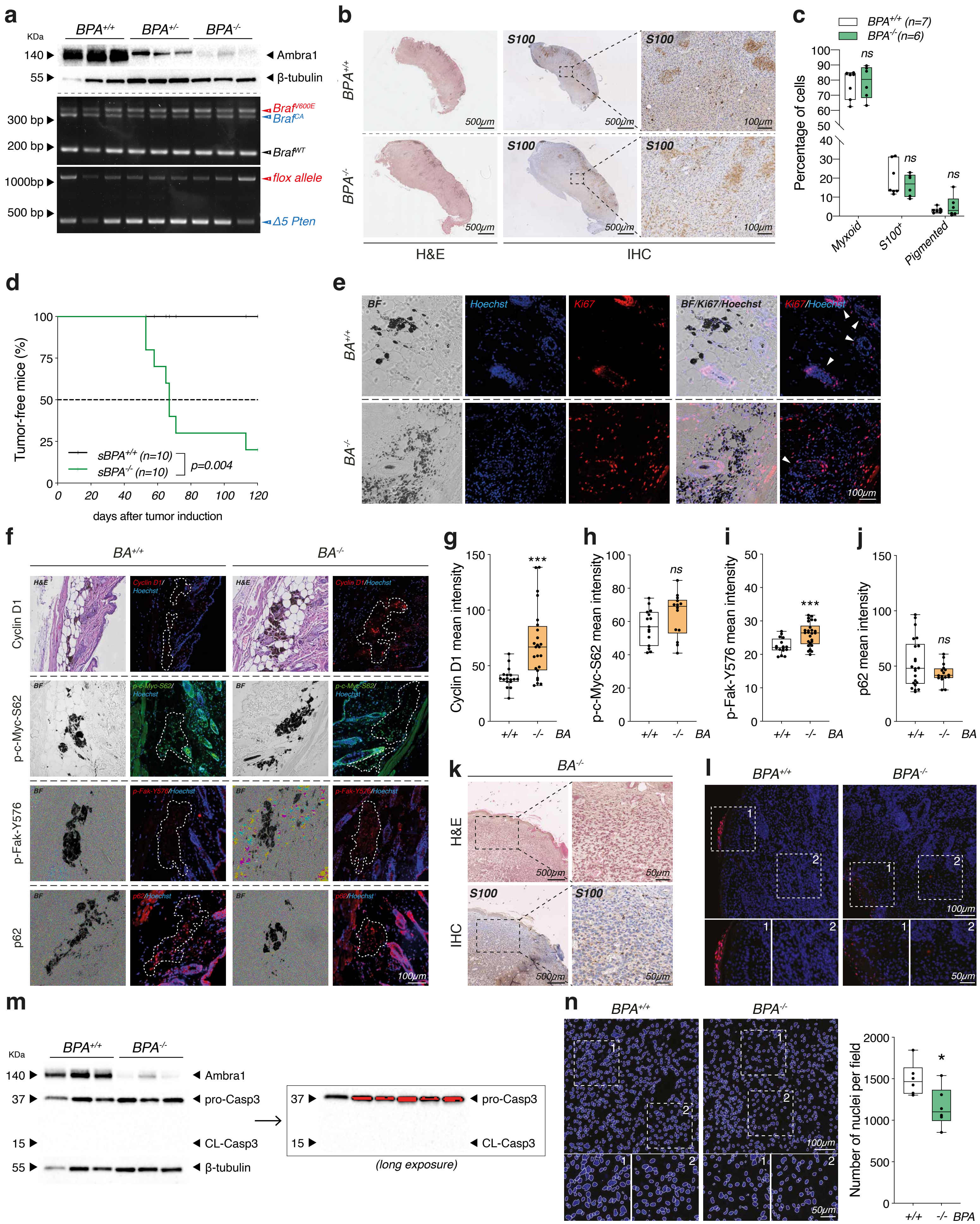

Supplementary Figure 2. Characterization of tumors and of hyperproliferation in the different GEMMs. (a) The Cre-mediated recombination of AMBRA1 (WB), *Braf* (*Braf*^{CA}, PCR) and *Pten* (*Δ5 Pten*, PCR) was determined in tumors from *BPA*$^{+/+}$ (*n*=3), *BPA*$^{+/-}$ (*n*=3) and *BPA*$^{-/-}$ (*n*=3) mice. ß-tubulin was used as loading control for AMBRA1. **(b)** H&E and S100 IHC staining and **(c)** corresponding quantification of FFPE-tumor sections from *BPA*$^{+/+}$ (*n*=7) and *BPA*$^{-/-}$ (*n*=6) mice. The S100 staining, associated with cell morphology, was used to evaluate the relative percentage of the three melanocytic cell types: pigmented (heavily pigmented melanocytes arranged in nests, positive for S100 in the cytoplasmic compartment); S100^{+} (low pigmentation, pleomorphic nuclei, growth in nests, stronger cytoplasmic S100 positivity); myxoid (no pigmentation, small monomorphic cells, weak/lost S100 expression). Histopathological analysis assessed tumors as Clark level V in both groups and no statistically significant difference was detected in the percentage of myxoid, S100^{+} and pigmented cells between groups (*ns*= not significant, two-tailed unpaired *t* test). **(d)** Mice were subcutaneously injected with 7×10^{5} of either *BPA*$^{+/+}$- (*sBPA*$^{+/+}$; *n*=10) or *BPA*$^{-/-}$- (*sBPA*$^{-/-}$; *n*=10) tumor-derived cells. Tumors appearance was monitored for 120 days. Two-sided log-rank test for comparisons of Kaplan-Meier survival curves indicates faster tumor incidence in *sBPA*$^{-/-}$ (7 out of 10) *vs sBPA*$^{+/+}$ mice (*p=0.004*). **(e)** Proliferation was assessed by Ki67 immunostaining (red) in FFPE-skin sections of *BA*$^{+/+}$ (*n*=7) and *BA*$^{-/-}$ (*n*=7) mice. Nuclei were counterstained with Hoechst (blue). Bright field (BF) images indicate areas of pigmentation and hair bulbs (white arrows) in *BA*$^{+/+}$ and *BA*$^{-/-}$ mice, respectively. Images are representative of each group. **(f)** IF analyses of Cyclin D1 (red), p-c-Myc-S62 (green), p-Fak-Y576 (red) and p62 (red) were performed on FFPE-skin sections of *BA*$^{+/+}$ (*n*=3) and *BA*$^{-/-}$ (*n*=3) mice. Nuclei were counterstained with Hoechst (blue). H&E or Bright field (BF) images indicate areas of pigmentation, which are outlined with a white dashed line in the merged IF pictures. Images are representative for each group. **(g)** The intensity of the nuclear signal of Cyclin D1 and of **(h)** p-c-Myc-S62 and the intensity of the cytosolic signal of **(i)** p-Fak-Y576 and of **(j)** p62 were determined (****p=0.0003 in* **(g)**; ****p=0.0009 in* **(i)**, *p>0.05 in* (***h***, ***j***), two-tailed unpaired *t* test, *BA*$^{-/-}$ *vs BA*$^{+/+}$). **(k)** H&E and S100 staining of the tumor that developed on the head of *BA*$^{-/-}$ mouse (1 out of 8), collected at endpoint (max size) 233 days after 4-OHT treatment. **(l)** TUNEL assay was used to determine cell death in FFPE-tumor sections from *BPA*$^{+/+}$ (*n*=6) and *BPA*$^{-/-}$ (*n*=6) mice. Areas of cell death (red) are shown at higher magnification. Nuclei were counterstained with Hoechst (blue). Images are representative of each group. **(m)** WB analyses of the apoptotic marker Casp3 and of its cleaved form (CL-Casp3) in bulk *BPA*$^{+/+}$ (*n*=3) and *BPA*$^{-/-}$ (*n*=3) tumor lysates. Ambra1 and ß-tubulin were used as genotype and loading control, respectively, and are as shown in Fig. 3f due to simultaneous detection of these markers in the same WB. A *long exposure* is shown for Casp3 (red bands identify signal saturation). **(n)** Nuclei of FFPE-tumor sections of *BPA*$^{+/+}$ (*n*=6) and *BPA*$^{-/-}$ (*n*=6) mice were counterstained with Hoechst and quantified. Each data point represents one mouse and corresponds to the average nuclei count ± SEM (at least five fields evaluated for each mouse) (**p=0.0411*, two-tailed unpaired *t* test, *BPA*$^{-/-}$ *vs BPA*$^{+/+}$). The box-and-whisker plots shown in **(c)**, **(g-j)** and **(n)** represent values from minimum to maximum. Top and bottom whiskers denote upper (Q_3) and lower (Q_1) quartiles, respectively, whereas boxes refer to interquartile ranges. Medians are denoted as horizontal line in the middle of the boxes.

Supplementary Figure 3

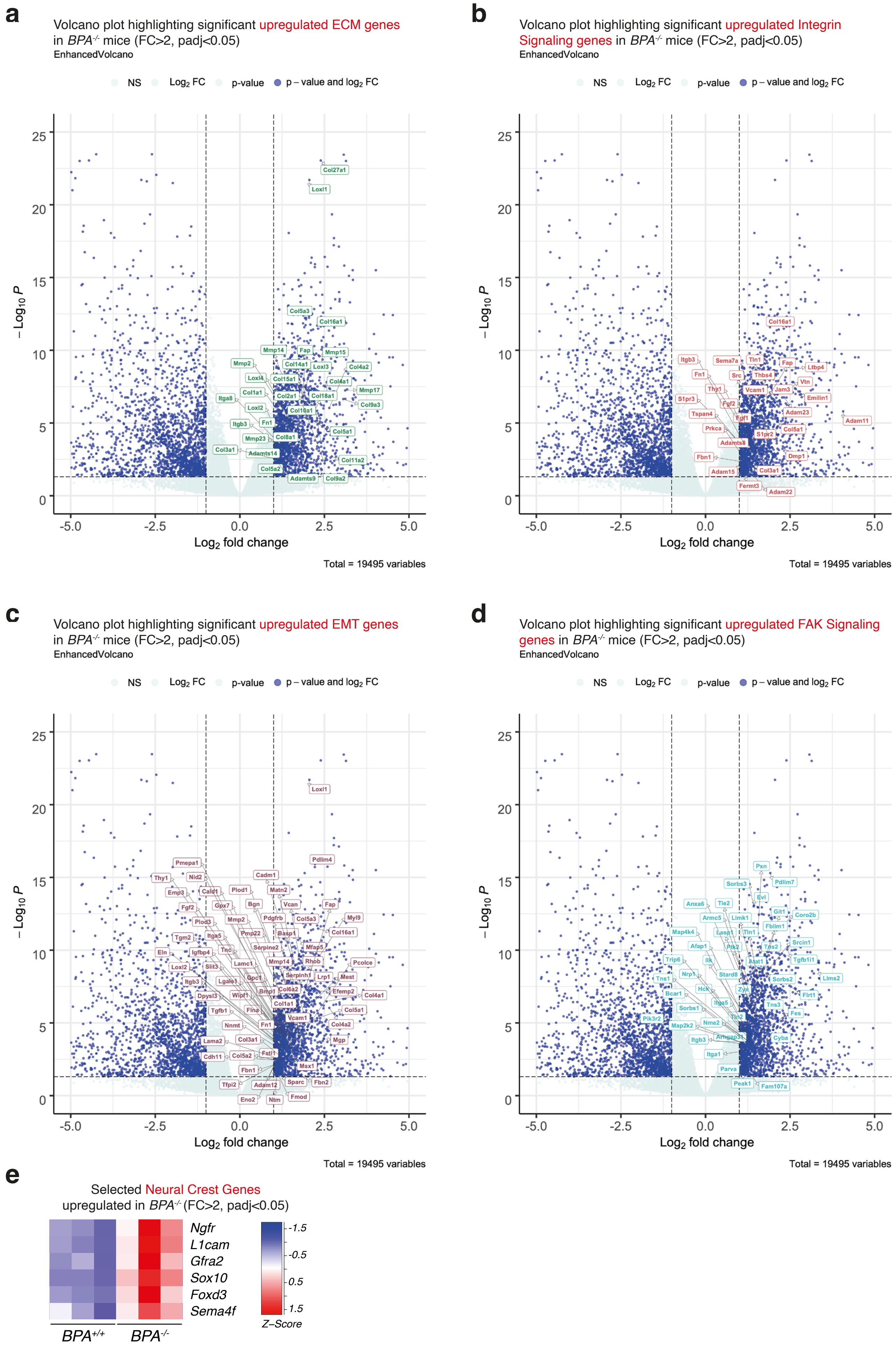

Supplementary Figure 3. Upregulated genes in bulk *BPA* tumors as from RNAseq analyses. (a) Volcano plots of differentially expressed genes from RNAseq analyses of *BPA*$^{+/+}$ (*n*=3) and *BPA*$^{-/-}$ (*n*=3) tumors. Upregulated genes related to **(a)** ECM organization, **(b)** Integrin signaling, **(c)** EMT and **(d)** focal adhesion kinase assembly in *BPA*$^{-/-}$ *vs BPA*$^{+/+}$ mice are highlighted in the boxes. *p*-values obtained by the Wald test are corrected for multiple testing using the Benjamini and Hochberg method. **(e)** The heatmap shows gene expression profile (RNAseq) of *BPA*$^{-/-}$ (*n*=3) *vs BPA*$^{+/+}$ (*n*=3) tumors for selected genes related to Nervous System Development. *FC*>2, *padj*<0.05.

Supplementary Figure 4

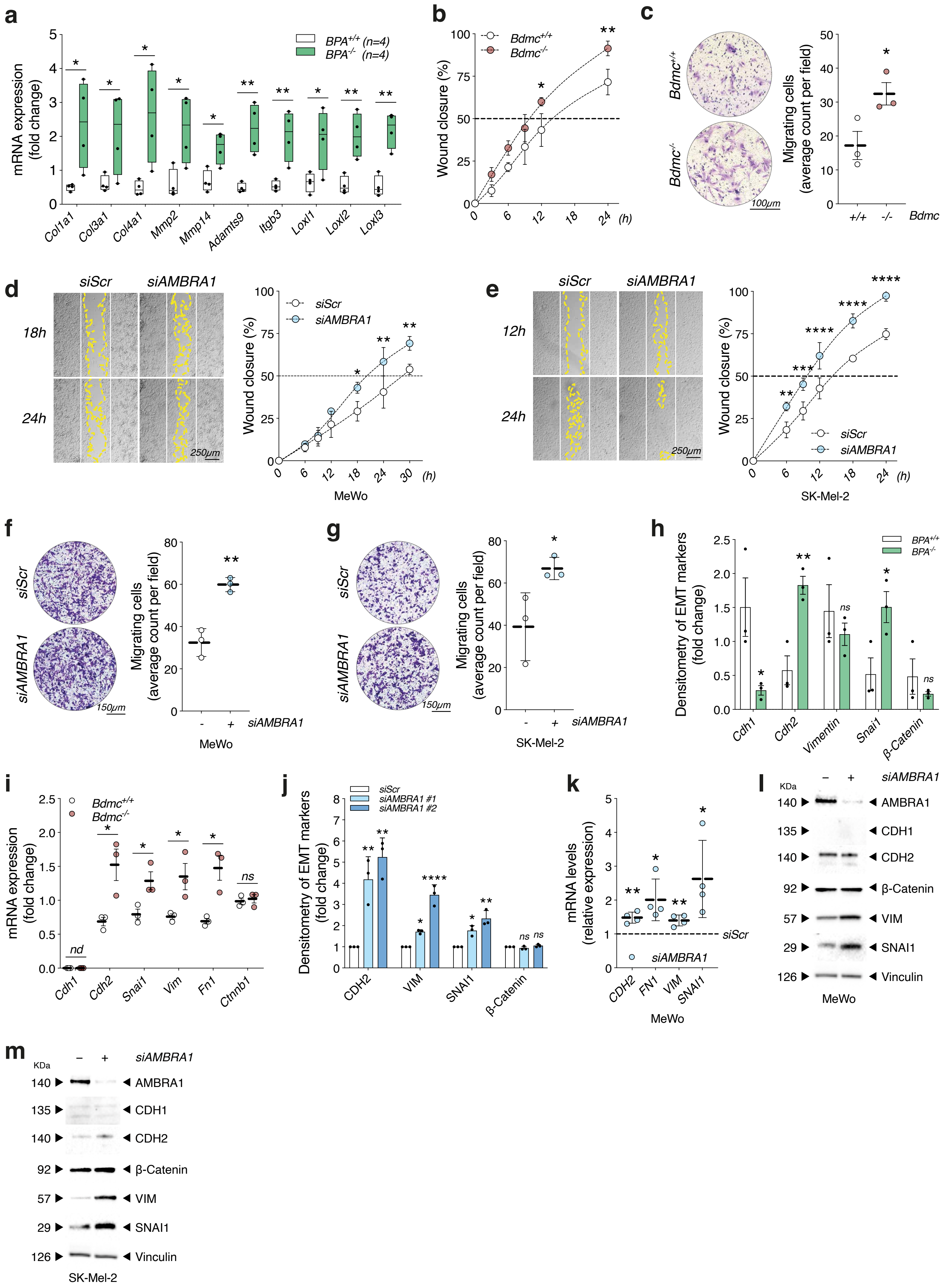

Supplementary Figure 4. Invasion and migration *ex vivo* and *in vitro* upon *AMBRA1* silencing. (a) RT-qPCR analyses of the ECM-related genes *Col1a1*, *Col3a1*, *Col4a1*, *Mmp2*, *Mmp14*, *Adamts9*, *Itgb3*, *Loxl1*, *Loxl2* and *Loxl3* in $BPA^{-/-}$ (*n*=4) and $BPA^{+/+}$ (*n*=4) mice. Data were normalized to the internal control *L34* and expressed as fold change with respect to the average C_t of $BPA^{+/+}$ mice (**p=0.0289 Col1a1*; **p=0.0498 Col3a1*; **p=0.0232 Col4a1*; **p=0.0249 Mmp2*; **p=0.0129 Mmp14*; ***p=0.0032 Adamts9*; ***p=0.0079 Itgb3*; **p=0.0337 Loxl1*; ***p=0.0058 Loxl2; **p=0.0016 Loxl3*, two-tailed unpaired *t* test, $BPA^{-/-}$ *vs* $BPA^{+/+}$). The box-and-whisker plot represents values from minimum to maximum. Top and bottom whiskers denote upper (Q_3) and lower (Q_1) quartiles, respectively, whereas boxes refer to interquartile ranges. Medians are denoted as horizontal line in the middle of the boxes. **(b)** Migration capacity of $Bdmc^{+/+}$ (*n*=3) and $Bdmc^{-/-}$ (*n*=3) cells was determined by Wound Healing Assay. The area of wound closure is shown as percentage ± SEM with respect to T_0 at 3h, 6h, 9h, 12h, 24h (**p=0.0102*; ***p=0.002*, two-way ANOVA, $Bdmc^{-/-}$ *vs* $Bdmc^{+/+}$). **(c)** Invasive capacity of $Bdmc^{+/+}$ (*n*=3) and $Bdmc^{-/-}$ (*n*=3) cells was assessed by Transwell Migration Assay after 24h. Cells were stained with Crystal Violet and the average count per field was determined, as shown in the dot plot to the right. Four different fields were evaluated for each condition and data are expressed as mean ± SEM. Images are representative of each group (**p=0.0452*, two-tailed unpaired *t* test, $Bdmc^{-/-}$ *vs* $Bdmc^{+/}$). **(d)** AMBRA1 was silenced in MeWo and **(e)** SK-Mel-2 cells and migration of cells determined by Wound Healing Assay. Images are representative on *n*=4 independent experiments. White and yellow lines outline the edge of the wound at T_0 and at the times indicated in the pictures, respectively. The area of wound closure is shown as percentage ± SD with respect to T_0 at the times indicated, as depicted in the graphs to the right (*n*=3; **p=0.0137* at 18h; ***p=0.001* at 24h; ***p=0.0053* at 30h, *siAMBRA1 vs siScr* in **(d)**, two-way ANOVA. *n*=3; ***p=0.0028* at 6h; ****p=0.0007* at 9h; *****p<0.0001* at 12h, 18h and 24h, *siAMBRA1 vs siScr* in **(e)**, two-way ANOVA). **(f)** AMBRA1 was silenced in MeWo and **(g)** SK-Mel-2 cells and invasive capacity of the cells was assessed by Transwell Migration Assay after 24h. Cells were stained with Crystal Violet and the average count per field was determined, as shown in the dot plots to the right. Four different fields for each condition were considered and data are expressed as mean ± SD. Images are representative of *n*=3 independent experiments (*n*=3; ***p=0.003* in **(f)**; **p=0.0476* in **(g)**, two-tailed unpaired *t* test, *siAMBRA1 vs siScr*). **(h)** Densitometry analyses of the EMT markers Cdh1, Cdh2, Vimentin, Snai1 and ß-Catenin detected in the bulk $BPA^{+/+}$ (*n*=3) and $BPA^{-/-}$ (*n*=3) tumor lysates shown in Fig. 3j. Protein levels were normalized on ß-tubulin and are shown as fold change ± SEM (**p=0.0487* Cdh1; ***p=0.0076* Cdh2; *p>0.05* Vimentin; **p=0.0409* Snai1; *p>0.05* ß-Catenin, two-tailed unpaired *t* test, $BPA^{-/-}$ *vs* $BPA^{+/+}$). **(i)** RT-qPCR analyses of the EMT genes *Cdh1*, *Cdh2*, *Snai1*, *Vim*, *Fn1* and *Ctnnb1* in $Bdmc^{+/+}$ (*n*=3) and $Bdmc^{-/-}$ (*n*=3) cells. Data were normalized to the internal control *L34* and expressed as fold change ± SEM with respect to the average C_t of $Bdmc^{+/+}$ cells (*nd= not detected*; **p=0.0417 Cdh2*; ****p=0.0006 Snai1*; **p=0.0239 Vim*; **p=0.0458 Fn1*; *p>0.05* ß-Catenin, two-tailed unpaired *t* test, $Bdmc^{-/-}$ *vs* $Bdmc^{+/+}$). **(j)** Densitometry analyses of the EMT markers CDH2, VIM, SNAI1 and ß-Catenin detected in the lysates of melanoma cells silenced for AMBRA1 shown in Fig. 3l. Protein levels were normalized on Vinculin and are shown as fold change ± SD (***p=0.006* CDH2; **p=0.0428* VIM; **p=0.0218* SNAI1, one-way ANOVA, *siAMBRA#1 vs siScr; **p=0.001* CDH2; *****p<0.0001* VIM; ***p=0.0015* SNAI1, one-way ANOVA, *siAMBRA#2 vs siScr*). **(k)** AMBRA1 was silenced in MeWo cells for 48h and mRNA expression of the EMT markers *CDH2*, *FN1*, *VIM* and *SNAI1* detected by RT-qPCR. Data were normalized on *L34* and expressed as fold change ± SD *vs siScr* (*n*=4; ***p=0.0042 CDH2*; **p=0.0212 FN1*; ***p=0.0037 VIM*; **p=0.0314 SNAI1*, two-tailed unpaired *t* test, *siAMBRA1 vs siScr*, represented by a dashed line). **(l)** WB analyses of the EMT markers CDH1, CDH2, ß-Catenin, VIM and SNAI1 in cell lysates from AMBRA1-silenced MeWo and **(m)** SK-Mel-2 cells after 48h silencing. AMBRA1 and Vinculin were used as transfection and loading control, respectively. Images are representative of *n*=4 independent experiments.

Supplementary Figure 5

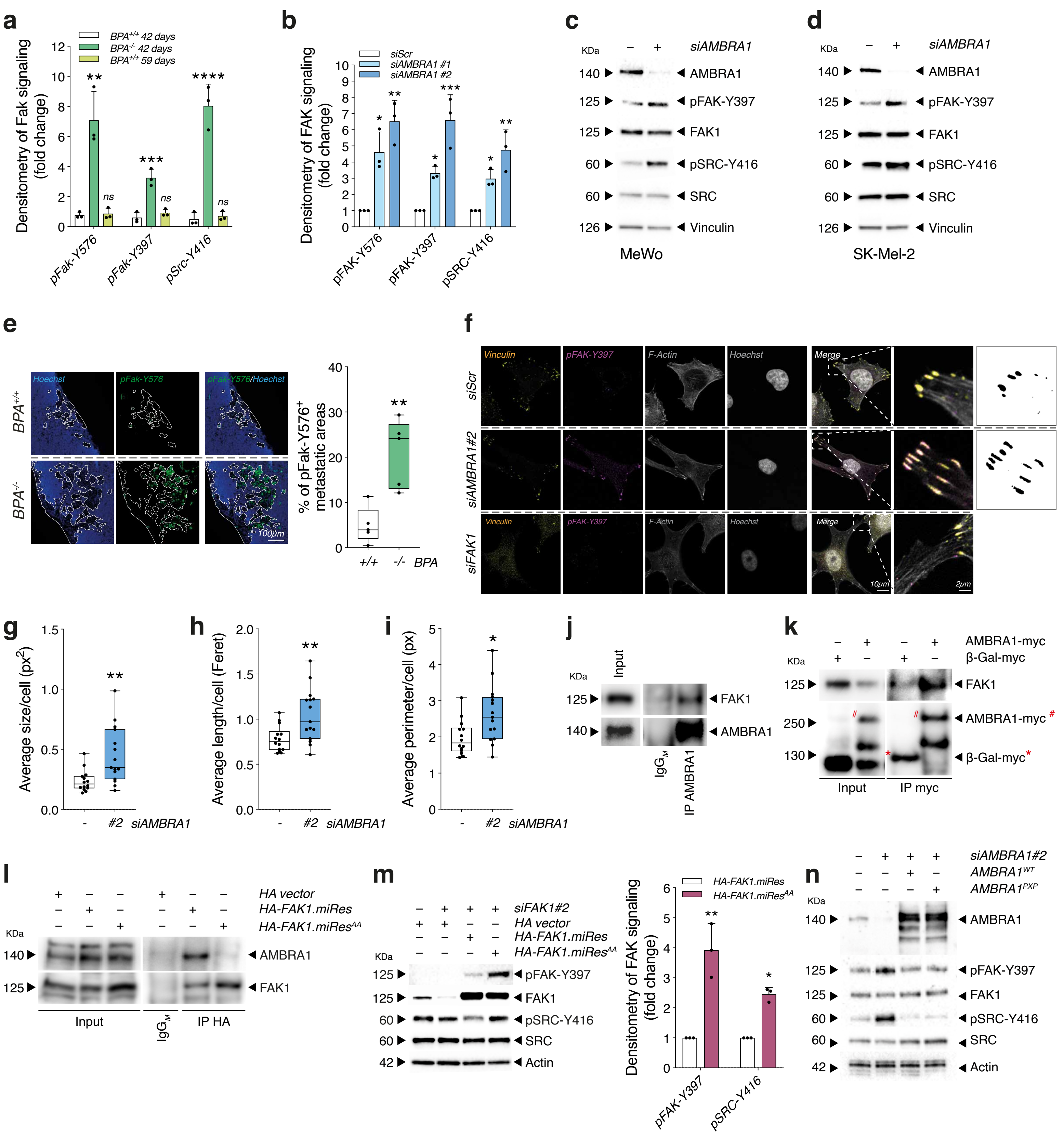

Supplementary Figure 5. Hyperactivation and characterization of FAK1 signaling in melanoma. (a) Densitometry analyses of pFak-Y576, pFak-Y397 and pSrc-Y416 detected in the bulk tumor lysates shown in Fig. 5a. The pFak-Y576/Fak1, pFak-Y397/Fak1 and pSrc-Y416/Src ratios were calculated and normalized on ß-tubulin and are shown as fold change ± SEM (*$**p=0.001$* pFak-Y576; *$***p=0.0005$* pFak-Y397; *$****p<0.0001$* pSrc-Y416, one-way ANOVA, *vs $BPA^{+/+}$ 42 days*). **(b)** Densitometry analyses of pFAK-Y576, pFAK-Y397 and pSRC-Y416 detected in the lysates of melanoma cells silenced for AMBRA1 shown in Fig. 5b. The pFAK-Y576/FAK1, pFAK-Y397/FAK1 and pSRC-Y416/SRC ratios were calculated and normalized on Vinculin and are shown as fold change ± SD (*$*p=0.0113$* pFAK-Y576; *$*p=0.0452$* pFAK-Y397; *$*p=0.045$* pSRC-Y416, one-way ANOVA, *siAMBRA#1 vs siScr; $**p=0.0013$* pFAK-Y576; *$***p=0.0007$* pFAK-Y397; *$**p=0.0023$* pSRC-Y416, one-way ANOVA, *siAMBRA#2 vs siScr*). **(c)** WB analyses of pFAK-Y397, FAK1, pSRC-Y416 and SRC in cell lysates from AMBRA1-silenced MeWo and **(d)** SK-Mel-2 cells after 48h silencing. AMBRA1 and Vinculin were used as transfection and loading control, respectively. Images are representative of *n*=4 independent experiments. **(e)** Immunostaining for p-Fak-Y576 (green) was performed in iLNs of $BPA^{+/+}$ (*n*=5) and $BPA^{-/-}$ (*n*=5). Nuclei were counterstained with Hoechst and shown in blue. Areas of pigmentation (metastases) are outlined in white. Images are representative for each group. The graph to the right refers to the percentage of p-Fak-$Y576^{+}$ area (signal) on area of pigmentation (*$**p=0.0079$*, two-tailed unpaired *t* test, *$BPA^{-/-}$ vs $BPA^{+/+}$*). **(f)** AMBRA1 was silenced in SK-Mel-5 (*siAMBRA1 #2*) as shown in Fig. 5b and IF staining of pFAK-Y397 performed to detect active FAs. Phalloidin was used to counterstain F-Actin fibers. Vinculin and Hoechst were used to counterstain cytoskeleton and nuclei, respectively. Images are representative of *n*=3 independent experiments. Panels on the right indicate thresholded images used for the quantifications displayed in **(g-i)**. To assess the immunostaining specificity also shown in Fig. 5c, FAK1 was also silenced in SK-Mel-5 cells (*siFAK1*) for 48h and cells stained as described in **(f)**. **(g)** The average size per cell, **(h)** the average length per cell (Feret), **(i)** the average perimeter per cell were determined (*n*=3; *$**p=0.0041$* in **(g)**; *$**p=0.0053$* in **(h)**; *$*p=0.0111$* in **(i)**, two-tailed unpaired *t* test, *siAMBRA1 #2 vs siScr*). **(j)** Immunoprecipitation was performed in endogenous protein extracts from SK-Mel-5 using an anti-AMBRA1 antibody (IP AMBRA1) and mouse immunoglobulins as control (IgG_M), **(k)** in extracts from AMBRA1-myc-tagged-expressing SK-Mel-5 cells using an anti-myc antibody (IP myc) and ß-Gal-myc-tagged-expressing extracts as control and **(l)** in extracts from HA-FAK1.miRes- and HA-FAK1.$miRes^{AA}$-expressing SK-Mel-5 cells using an anti-HA (IP HA) and an HA-expressing plasmid as control (IP Ctrl). Purified complexes and corresponding total extracts (Input) were analyzed by WB using anti-AMBRA1, anti-MYC and anti-FAK1 antibodies. **(m)** After 24 h from FAK1 silencing (*siFAK1#2*), SK-Mel-5 were transfected with HA-FAK1.miRes- and HA-FAK1.$miRes^{AA}$-expressing plasmids and WB analyses performed to detect pFAK-Y397, FAK1, pSRC-Y416 and SRC levels. Actin was used as loading control. Images are representative of *n*=3 independent experiments. The graph to the right refers to the densitometry analyses of pFAK-Y397 and pSRC-Y416. The pFAK-Y397/FAK1 and pSRC-Y416/SRC ratios were calculated and normalized on Actin and are shown as fold change ± SD (*n*=3; *$**p=0.003$* pFAK-Y397; *$*p=0.0364$* pSRC-Y416, one-way ANOVA, *HA-FAK1.$miRes^{AA}$ vs HA-FAK1.miRes*). **(n)** Sk-Mel-5 were treated with an siRNA targeting the 5'-UTR region of AMBRA1 (*siAMBRA1#2*) for 24 h and transfected with $AMBRA1^{WT}$- and $AMBRA1^{PXP}$-expressing plasmids for additional 24 h. WB analyses were performed to detect pFAK-Y397, FAK1, pSRC-Y416 and SRC levels. AMBRA1 and Actin were used as transfection and loading control, respectively. Images are representative of *n*=3 independent experiments. The box-and-whisker plots shown in **(e)** and **(g-i)** represent values from minimum to maximum. Top and bottom whiskers denote upper (Q_3) and lower (Q_1) quartiles, respectively, whereas boxes refer to interquartile ranges. Medians are denoted as horizontal line in the middle of the boxes.

Supplementary Figure 6

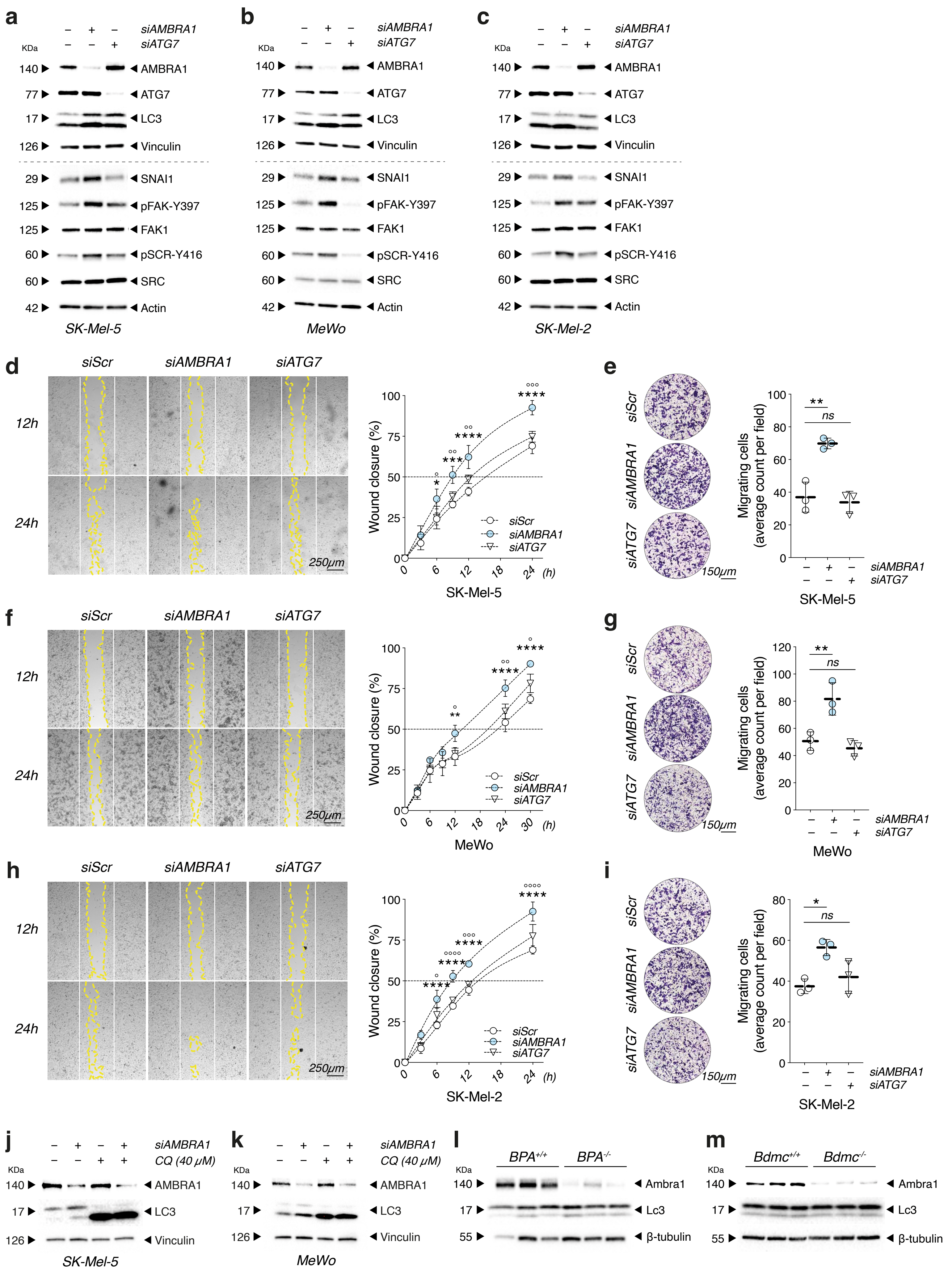

Supplementary Figure 6. The activation of the FAK1 signaling axis is independent from the autophagy functions of AMBRA1. (a) AMBRA1 (*siAMBRA1*) and ATG7 (*siATG7*) were silenced in SK-Mel-5, **(b)** MeWo and **(c)** SK-Mel-2 cells for 48h and the autophagy marker LC3, the FAK1 signaling markers (pFAK-Y397, FAK1, pSRC-Y416 and SRC) and the EMT marker SNAI1 were detected by WB. AMBRA1 and ATG7 were used as transfection controls, whilst Vinculin and Actin were used as loading controls. Images are representative of *n*=3 independent experiments. **(d)** AMBRA1 and ATG7 were silenced in SK-Mel-5, **(f)** MeWo and **(h)** SK-Mel-2 cells, as previously described, and migration capacity was determined by Wound Healing Assay. Images are representative on *n*=3 independent experiments. White and yellow lines outline the edge of the wound at T_0 and at the times indicated in the pictures, respectively. Area of wound closure is shown as percentage ± SD with respect to T_0 at the times indicated (in **(d)** *n*=3, **p=0.019* at 6h; ****p=0.0001* at 9h; *****p<0.0001* at 12h and 24h, *siAMBRA1 vs siScr*; °p=0.0376 at 6h; °°p=0.0079 at 9h; °°p=0.0057 at 12h; °°°p=0.0002 at 24h, *siAMBRA1 vs siATG7.* In **(f)** *n*=3, ***p=0.025* at 12h; *****p<0.0001* at 24h and 30h, *siAMBRA1 vs siScr*; °p=0.0*118* at 12h; °°p=0.00*25* at 24h; °p=0.0*185* at 30h, *siAMBRA1 vs siATG7.* In **(h)** *n*=3, *****p<0.0001* at 6h, 9h, 12h and 24h, *siAMBRA1 vs siScr*; °p=0.0197 at 6h; °°°°p<0.0001 at 9h and 24h; °°°p=0.0006 at 12h, *siAMBRA1 vs siATG7.* Two-way ANOVA). **(e)** Invasive capacity was also assessed under similar conditions in SK-Mel-5, **(g)** MeWo and **(i)** SK-Mel-2 by Transwell Migration Assay after 24h. Cells were stained with Crystal Violet and the average count per field was determined, as shown in the dot plots to the right. Four different fields were analyzed for each condition and data are expressed as mean ± SD. Images are representative of *n*=3 independent experiments (*n*=3; ***p=0.0022* in **(e)**; ***p=0.0089* in **(g)**; **p=0.0118* in **(i)**, one-way ANOVA). **(j)** AMBRA1 was silenced in SK-Mel-5 and **(k)** MeWo for 24h and cells were treated with 40 μM chloroquine (*CQ*) for 2h to block autophagy flux. WB of the autophagy marker LC3 is shown. AMBRA1 and Actin were used as transfection and loading control, respectively (*n*=3). **(l)** WB analyses of the autophagy marker Lc3 in bulk $BPA^{+/+}$ (*n*=3) and $BPA^{-/-}$ (*n*=3) tumor lysates. Ambra1 and ß-tubulin were used as genotype and loading control, respectively. Ambra1 and ß-tubulin images are as shown in Fig. 5a since Lc3 was detected simultaneously in the same WB. **(m)** WB analyses of the autophagy marker Lc3 in $Bdmc^{+/+}$ (*n*=3) and $Bdmc^{-/-}$ (*n*=3) cells. Ambra1 and ß-tubulin were used as genotype and loading control, respectively.

Supplementary Figure 7

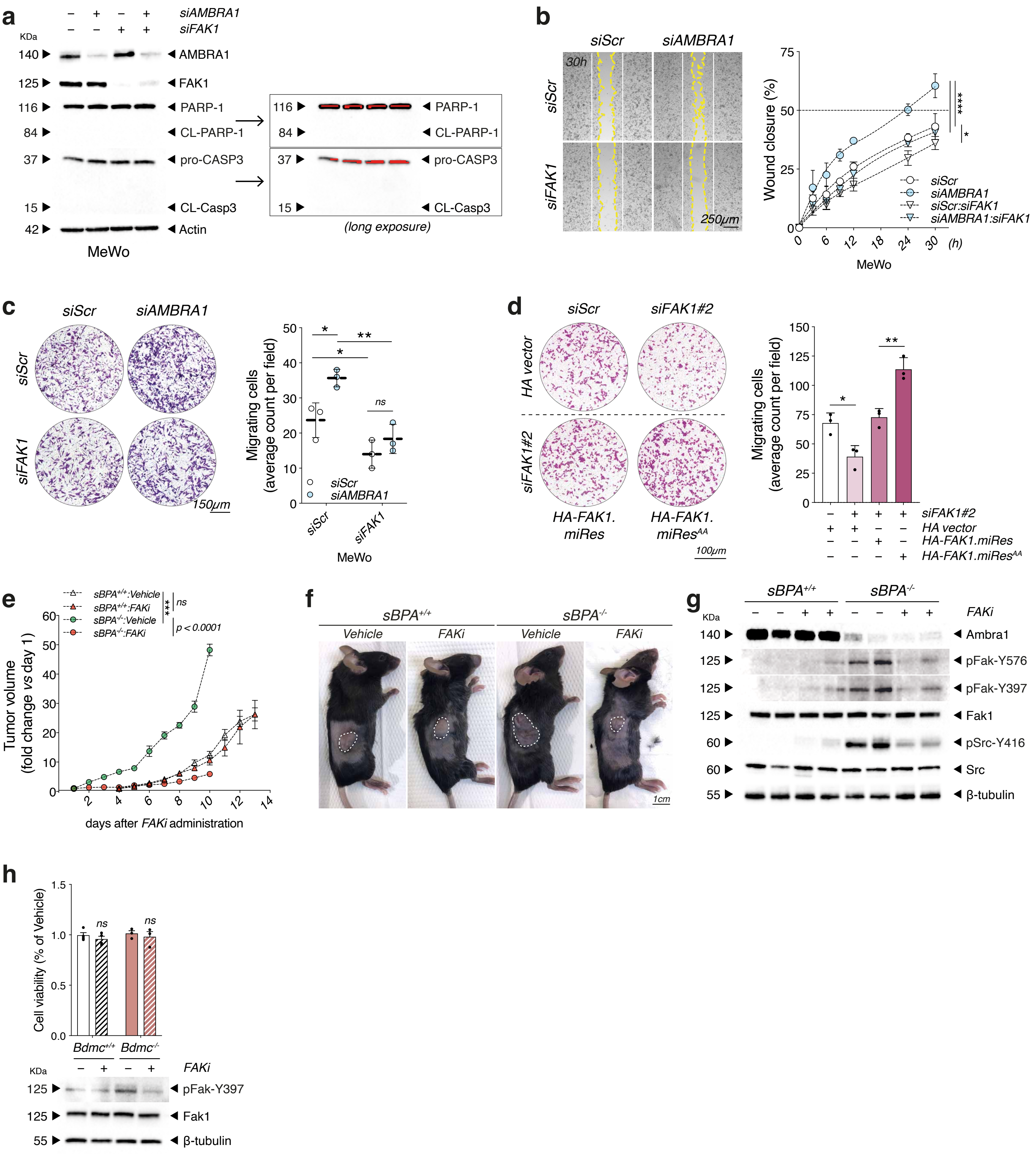

Supplementary Figure 7. Effect of FAK1 modulation *in vitro* and *in vivo.* (a) MeWo were co-silenced for both AMBRA1 and FAK1 for 48h. Cleavage of the apoptotic markers PARP-1 (CL-PARP-1) and CASP-3 (CL-CASP3) were detected by WB. *Long exposure* for PARP-1 and CASP3 is provided (red bands identify signal saturation). AMBRA1 and FAK1 were used as transfection controls, whilst Actin was used as loading control. Images are representative of *n*=3 independent experiments. **(b)** MeWo were transfected as in **(a)** and migration was determined by Wound Healing Assay. Images are representative on *n*=3 independent experiments. White and yellow lines outline the edge of the wound at T_0 and at 30h, respectively. Area of wound closure is shown as percentage ± SD with respect to T_0 at the times indicated (T_0, 6h, 9h, 12h, 24h, 30h). For clarity, statistical analyses and images are only shown for time point 30h (**p=0.037*; *****p<0.0001*, two-way ANOVA). **(c)** MeWo were transfected as in **(a)** and invasive capacity of the cells was assessed by Transwell Migration Assay after 24h. Cells were stained with Crystal Violet and the average count per field was determined, as shown in the dot plot to the right. Four different fields were counted for each condition and data are expressed as mean ± SD. Images are representative of *n*=3 independent experiments (*n*=3; **p=0.0259 siScr:siAMBRA1 vs siScr:siScr*; ***p=0.0032 siFAK1:siAMBRA1 vs siScr:siAMBRA1*; **p=0.0429 siScr:siFAK1 vs siScr:siScr*, two-way ANOVA). **(d)** After 24 h from FAK1 silencing (*siFAK1#2*), SK-Mel-5 were transfected with HA-FAK1.miRes- and HA-FAK1.miRes^{AA}-expressing plasmids and invasive capacity of the cells was assessed by Transwell Migration Assay after 24h. Cells were stained with Crystal Violet and the average count per field was determined, as shown in the graph to the right. Four different fields were counted for each condition and data are expressed as mean ± SD. Images are representative of *n*=3 independent experiments (*n*=3; **p=0.0275*; ***p=0.0032*, two-way ANOVA). **(e)** C57Bl/6 mice were injected with 5×10^6 of either *BPA$^{+/+}$*- (*sBPA$^{+/+}$*) or *BPA$^{-/-}$*- (*sBPA$^{-/-}$*) tumor-derived cells. When tumors were measurable (3×3 mm), mice were IP-administered with either *Vehicle* or *FAKi* (*sBPA$^{+/+}$:Vehicle n*=8; *sBPA$^{+/+}$:FAKi n*=6; *sBPA$^{-/-}$:Vehicle n*=3; *sBPA$^{-/-}$:FAKi n*=2-5) and tumor volumes were calculated using the formula: $V=(W\times L^2)/2$, where W>L and W=Width, L=Length. Each data point corresponds to the average fold change of tumor volume with respect to day 1 ± SEM, as determined for each experimental group. For clarity, statistical analysis is shown only at endpoint 10 days after first IP administration (*ns*= not significant; ****p=0.0004*, two-way ANOVA) **(f)** Representative images of the tumors of the kinetics shown in **(e)** at endpoint. **(g)** WB analyses were performed on bulk tumors from *sBPA$^{+/+}$* and *sBPA$^{-/-}$* mice treated with either *Vehicle* or *FAKi* to assess the levels of pFak-Y576, pFak-Y397, Fak1, pSrc-Y416 and Src. Ambra1 and ß-tubulin were used as genotype and loading control, respectively. **(h)** Bdmc$^{+/+}$ (*n*=3) and Bdmc$^{-/-}$ (*n*=3) cells were treated with 1 μM final concentration of *FAKi* for 24 h and WB analyses of p-Fak-Y397 and Fak1 performed to assess the efficiency of inhibition. ß-tubulin was used as loading control. Images are representative of *n*=3 independent experiments. Cell viability was also assessed in the same conditions and expressed as percentage ± SEM *vs Vehicle* (*n*=3; *ns*= not significant, two-way ANOVA, *FAKi vs Vehicle*).

Supplementary Figure 8

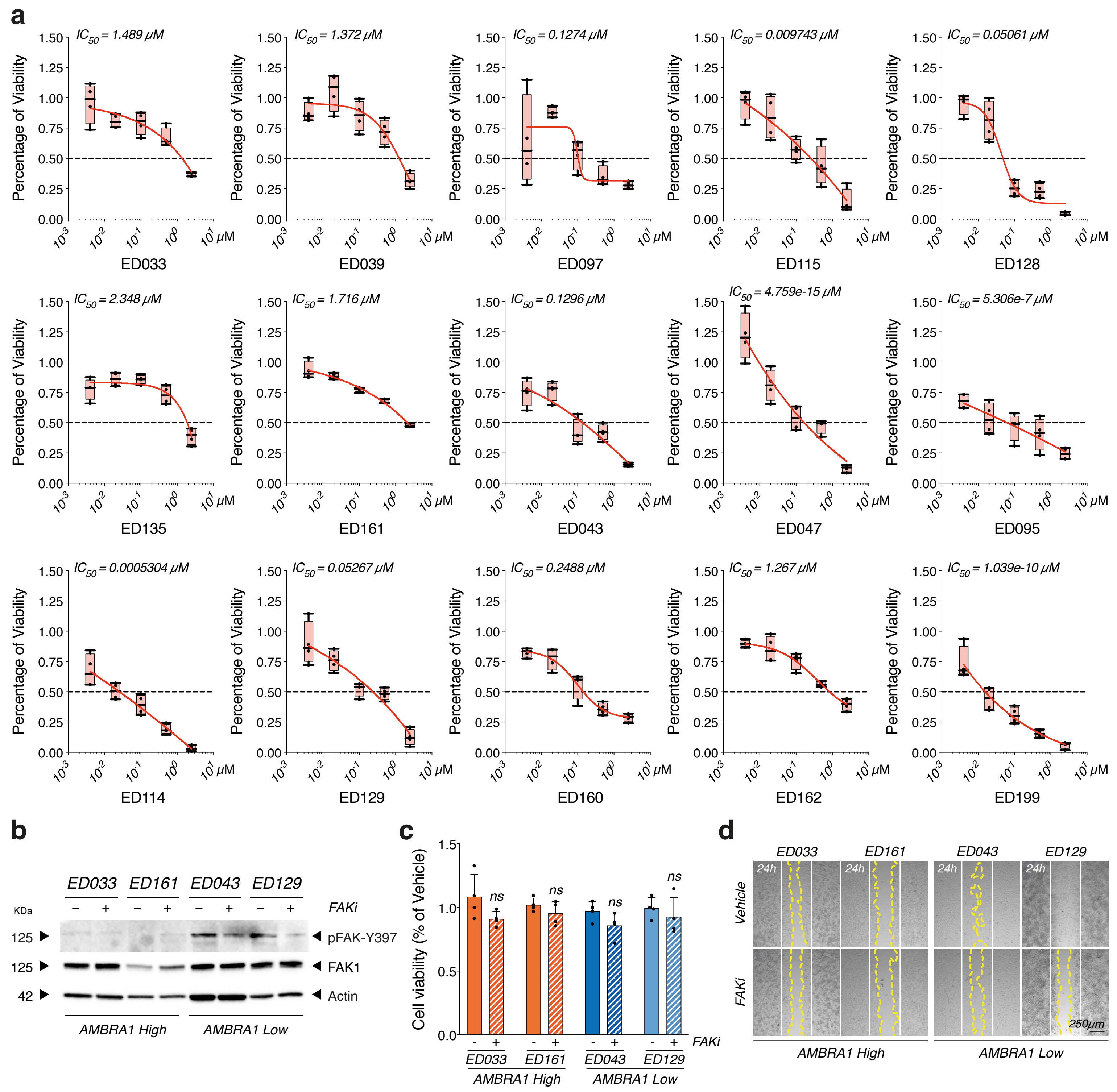

Supplementary Figure 8. FAKi sensitivity of ESTDAB cells. ***(a)*** AMBRA1 High ED033, ED039, ED097, ED115, ED128, ED135 and ED161 and AMBRA1 Low ED043, ED047, ED095, ED114, ED129, ED160, ED162 and ED199 melanoma cells were treated with *FAKi* at the final concentrations of 0.004 μM, 0.02 μM, 0.1 μM, 0.5 μM and 2.5 μM for 24 h and cell viability assessed. Data are shown as percentage of living cells with respect to untreated (*Vehicle*) cells. For each cell line, the IC_{50} was calculated and reported on the respective graph (*n*=4). The box-and-whisker plot represents values from minimum to maximum. Top and bottom whiskers denote upper (Q_3) and lower (Q_1) quartiles, respectively, whereas boxes refer to interquartile ranges. Medians are denoted as horizontal line in the middle of the boxes. **(b-d)** A selection of 2 AMBRA1 High (ED033, ED161) and of 2 AMBRA1 Low (ED043, ED129) melanoma cell lines were treated with non-lethal doses of *FAKi* (ED033: 1 μM; ED161; 1 μM; ED043: 0.1 μM; ED129: 0.01 μM) for 24 h. **(b)** WB analyses of p-FAK-Y397 and FAK1 were performed to assess the efficiency of inhibition. Actin was used as loading control. Images are representative of *n*=3 independent experiments. **(c)** Cell viability was also assessed upon *FAKi* treatment in the same cells shown in **(b)** and expressed as percentage ± SD *vs Vehicle* (*n*=3; *ns*= not significant, two-tailed unpaired *t* test, *FAKi vs Vehicle*). **(d)** Migration capacity of the selected AMBRA1 High and Low cells was also evaluated upon *FAKi* by Wound Healing Assay. Images are representative of *n*=3 independent experiments. White and yellow lines outline the edge of the wound at T_0 and at 24h, respectively.

Supplementary Table 1. Summary of outcomes for 4-OHT induction in *BA*$^{-/-}$ and *BA*$^{+/+}$ mice, related to Figure 1. Mice showing signs of sickness (paralyzed hind legs, not moving, cyst on hind leg or in the abdomen) were sacrificed at the timepoints indicated in observance of the legislation regarding the health status of mice.

Group	***Endpoint***	***Timepoint***
BA$^{+/+}$	No tumor/Paralyzed hind legs	233 days
	No tumor	350 days
	No tumor	350 days
	No tumor/Sick, not moving	233 days
	No tumor	350 days
	No tumor	350 days
	No tumor/Cyst on hind leg	334 days
	No tumor	350 days
	No tumor	350 days
	No tumor	350 days
	No tumor	350 days
	No tumor	350 days
	No tumor	350 days
BA$^{+/-}$	No tumor	350 days
	No tumor	350 days
	No tumor	350 days
	No tumor	350 days
	No tumor	350 days
	No tumor	350 days
	No tumor	350 days
BA$^{-/-}$	Primary tumor burden (**Supplementary Figure 2k**)	233 days
	No tumor/Abdominal cyst	304 days
	No tumor/Abdominal cyst	307 days
	No tumor	350 days
	No tumor	350 days
	No tumor	350 days
	No tumor	350 days
	No tumor	350 days

Supplementary Table 2. List of primary antibodies used for WB analyses.

Target	*Dilution*	*Distributor*	*Cat#*	*RRID*
β-Catenin	1:1,000	Cell Signaling Technology, MA, USA	8480	AB_11127855
β-tubulin	1:5,000	Cell Signaling Technology, MA, USA	2128S	AB_823664
Actin	1:40,000	Novus Biologicals, CO, USA	NB600-501	AB_10077656
AMBRA1	1:1,000	Santa Cruz Biotechnology, TX, USA	sc-398204	*NA*
Ambra1	1:1,000	Merck-Millipore MA, USA	ABC131	AB_2636939
Atg7	1:2,000	Cell Signaling Technology, MA, USA	8558S	AB_10831194
c-myc	1:1,000	Santa Cruz Biotechnology, TX, USA	sc-40	*NA*
CASP-3	1:1,000	Cell Signaling Technology, MA, USA	9662S	AB_331439
CDH1	1:1,000	Cell Signaling Technology, MA, USA	3195S	AB_2291471
CDH2	1:1,000	Cell Signaling Technology, MA, USA	13116S	AB_2687616
Cyclin D1	1:2,000	Abcam, UK	ab16663	AB_443423
FAK	1:1,000	Cell Signaling Technology, MA, USA	13009S	AB_2798086
LC3	1:2,500	Cell Signaling Technology, MA, USA	3868S	AB_2137707
PARP-1	1:1,000	Enzo Life Sciences, NY, USA	BML-SA250	AB_2160745
pFAK-Y397	1:1,000	Cell Signaling Technology, MA, USA	8556S	AB_10891442
pFAK-Y576	1:750	ThermoFisher Scientific, MA, USA	PA5-104964	AB_2816437
pSRC-Y416	1:1,000	Cell Signaling Technology, MA, USA	6943S	AB_10013641
SNAI1	1:1,500	Cell Signaling Technology, MA, USA	3879S	AB_2255011
SRC	1:1,000	Cell Signaling Technology, MA, USA	2123S	AB_2106047
Vimentin	1:4,000	Cell Signaling Technology, MA, USA	5741S	AB_10695459
Vinculin	1:4,000	Sigma-Aldrich, MO, USA	V4505	AB_477617

Supplementary Table 3. List of primer pairs used for RT-qPCR.

Target	*Species*	*Forward primers*	*Reverse primers*
CDH1	Human	5'- CAG GCC TCC GTT TCT GGA AT -3'	5'- GTC TCT CTT CTG TCT TCT GAG GC -3'
CDH2	Human	5'- AGG CTT CTG GTG AAA TCG CA -3'	5'- GCA GTT GCT AAA CTT CAC ATT GAG -3'
FN1	Human	5'- CGA CAC ATT CCA CAA GCG TC -3'	5'- CAT TGG TCG ACG GGA TCA CA -3'
L34	Human	5'- GGC CCT GCT GAC ATG TTT CTT -3'	5'- GTC CCG AAC CCC TGG TAA TAG A -3'
SNAI1	Human	5'- CGA GTG GTT CTT CTG CGC TA -3'	5'- CTG CTG GAA GGT AAA CTC TGG A -3'
VIM	Human	5'- GAC GCC ATC AAC ACC GAG TT -3'	5'- CTT TGT CGT TGG TTA GCT GGT -3'
Adamts9	Mouse	5'- GAC TTG TGG GCA AGG TAA GG -3'	5'- TCA GTC TCG GGG ATG TAA TCT G -3'
Cdh1	Mouse	5'- GGA CGT CCA TGT GTG TGA CT -3'	5'- GAT CAG AAT CAG CAG GGC GA -3'
Cdh2	Mouse	5'- GTG ATA CGC TTC AAC CCA CT -3'	5'- CCA TTT TTA CAG CGA GGT GG -3'
Col1a1	Mouse	5'- CCC TGG AAT GAA GGG ACA CC -3'	5'- GAG CTC CGT TTT CAC CAG GA -3'
Col3a1	Mouse	5'- CTG TAA CAT GGA AAC TGG GGA AA -3'	5'- CCA TAG CTG AAC TGA AAA CCA CC -3'
Col4a1	Mouse	5'- CTG GCA CAA AAG GGA CGA G -3'	5'- ACG TGG CCG AGA ATT TCA CC -3'
Ctnnb1	Mouse	5'- AGC TCG TGT CCT GTG AAG C -3'	5'- CAG GTC AGC TTG AGT AGC CAT -3'
Fn1	Mouse	5'- ATG TGG ACC CCT CCT GAT AGT -3'	5'- GCC CAG TGA TTT CAG CAA AGG -3'
Itgb3	Mouse	5'- CCA CAC GAG GCG TGA ACT C -3'	5'- CTT CAG GTT ACA TCG GGG TGA -3'
L34	Mouse	5'- GGT GCT CAG AGG CAC TCA GGA TG -3'	5'- GTG CTT TCC CAA CCT TCT TGG TGT -3'
Loxl1	Mouse	5'- GAG TGC TAT TGC GCT TCC C -3'	5'- GGT TGC CGA AGT CAC AGG T -3'
Loxl2	Mouse	5'- ATT AAC CCC AAC TAT GAA GTG CC -3'	5'- CTG TCT CCT CAC TGA AGG CTC -3'
Loxl3	Mouse	5'- CTA CTG CTG CTA CAC TGT CTG T -3'	5'- GAC CTT CAT AGG GCT TTC TAG GA -3'
Mmp14	Mouse	5'- CAG TAT GGC TAC CTA CCT CCA G -3'	5'- GCC TTG CCT GTC ACT TGT AAA -3'
Mmp2	Mouse	5'- CAA GTT CCC CGG CG ATG TC -3'	5'- TTC TGG TCA AGG TCA CCT GTC -3'
Snai1	Mouse	5'- TGT GTG GAG TTC ACC TTC CAG -3'	5'- GGT ACC AGG AGA GAG TCC CA -3'
Vim	Mouse	5'- CGT CCA CAC GCA CCT ACA G -3'	5'- GGG GGA TGA GGA ATA GAG GCT -3'
