# Supplementary material for: Loss of Ambra1 promotes melanoma growth and invasion
Source: Nat Commun. 2021 May 5;12:2550. doi: 10.1038/s41467-021-22772-2 (PMC8100102; doi:10.1038/s41467-021-22772-2)
Supplement: Supplementary file 3 — Description of Additional Supplementary Files [file 41467_2021_22772_MOESM3_ESM.pdf]

**Description of Additional Supplementary Files**

File Name: Supplementary Data 1
Description: Upregulated processes in $BPA^{-/-}$ mice (Fisher exact test).

File Name: Supplementary Data 2
Description: RNAseq_metadata.
