# Supplementary material for: Loss of Ambra1 promotes melanoma growth and invasion
Source: Nat Commun. 2021 May 5;12:2550. doi: 10.1038/s41467-021-22772-2 (PMC8100102; doi:10.1038/s41467-021-22772-2)
Supplement: Supplementary file 6 — Reporting Summary [file 41467_2021_22772_MOESM6_ESM.pdf]

nature research

Corresponding author(s): Daniela De Zio and Francesco Cecconi

Last updated by author(s): Mar 9, 2021

Reporting Summary

Nature Research wishes to improve the reproducibility of the work that we publish. This form provides structure for consistency and transparency in reporting. For further information on Nature Research policies, see our Editorial Policies and the Editorial Policy Checklist.

Statistics

For all statistical analyses, confirm that the following items are present in the figure legend, table legend, main text, or Methods section.

n/a	Confirmed	
☐	☒	The exact sample size (*n*) for each experimental group/condition, given as a discrete number and unit of measurement
☐	☒	A statement on whether measurements were taken from distinct samples or whether the same sample was measured repeatedly
☐	☒	The statistical test(s) used AND whether they are one- or two-sided<br>*Only common tests should be described solely by name; describe more complex techniques in the Methods section.*
☒	☐	A description of all covariates tested
☐	☒	A description of any assumptions or corrections, such as tests of normality and adjustment for multiple comparisons
☐	☒	A full description of the statistical parameters including central tendency (e.g. means) or other basic estimates (e.g. regression coefficient) AND variation (e.g. standard deviation) or associated estimates of uncertainty (e.g. confidence intervals)
☐	☒	For null hypothesis testing, the test statistic (e.g. *F*, *t*, *r*) with confidence intervals, effect sizes, degrees of freedom and *P* value noted<br>*Give P values as exact values whenever suitable.*
☒	☐	For Bayesian analysis, information on the choice of priors and Markov chain Monte Carlo settings
☒	☐	For hierarchical and complex designs, identification of the appropriate level for tests and full reporting of outcomes
☐	☒	Estimates of effect sizes (e.g. Cohen's *d*, Pearson's *r*), indicating how they were calculated

Our web collection on statistics for biologists contains articles on many of the points above.

Software and code

Policy information about availability of computer code

Data collection	Zeiss ZEN software v2.3<br>Leica Application Suite v4.2.0<br>CellSens Imaging Software 2<br>Image Lab 6.0.1 Software<br>NanoZoomer-XR Digital slide scanner<br>ViiA 7 Real-Time PCR System v1.3<br>Illumina NextSeq<br>TCGAbiolinks R package 2.18.0
Data analysis	ZEN lite 2012 software Service Pack 2<br>Huygens Professional software (Scientific Volume Imaging B.V.) engine 20.10.0p2<br>Fiji analysis software (based on ImageJ v: 2.1.0/1.53c)<br>NDP.view 2, version 2.7<br>QuPath version v0.1.2<br>Image Lab 6.0.1 Software<br>ImageJ version 1.52.n<br>ViiA 7 Real-Time PCR System v1.3<br>GraphPad Prism 8<br>Adobe Illustrator CC, 2020<br>GSEA java desktop application for Mac version 4.0.3<br>R version 3.6.0<br>FastQC v0.11.9<br>Cutadapt v1.18<br>R package LUMI 2.38.0

STAR version 2.5.3a
featureCount Release 1.6.3
DESeq2 version 1.4.02
TCGABiolinks 2.18.0
EnrichR
iGenomes
MaxEntropy algorithm

For manuscripts utilizing custom algorithms or software that are central to the research but not yet described in published literature, software must be made available to editors and reviewers. We strongly encourage code deposition in a community repository (e.g. GitHub). See the Nature Research guidelines for submitting code & software for further information.

Data

Policy information about availability of data

All manuscripts must include a data availability statement. This statement should provide the following information, where applicable:

- Accession codes, unique identifiers, or web links for publicly available datasets
- A list of figures that have associated raw data
- A description of any restrictions on data availability

The RNAseq datasets from the TCGA-SKCM analysed during the current study are available on The Cancer Genome Atlas (TCGA; RRID:SCR_003193) and were downloaded 07/10/19 using TCGAbiolinks.

The RNAseq data from CCLE melanoma cell lines analysed during the current study are available on CCLE website (portals.broadinstitute.org/ccle).
The transcriptomic data from the LMC analysed during the current study were generated by the University of Leeds in connection with the project 'The Leeds Melanoma Cohort'; otherwise known as Melanoma Follow-up and Case-Control Family Study (REC reference number 01/03/057). These data are available within the European Genome-phenome Archive at the European Bioinformatics Institute (accession number EGAS00001002922)

The RNAseq data generated from BPA+/+ and BPA-/- samples are publicly available under the GEO accession number GSE151134.

The authors declare that all other data supporting the findings of this study are available within the paper and its supplementary information files. Source data for figure(s) [1-6, Supplementary 2-8] are provided with the paper as a separate "Data Source" folder, which includes an Excel file with raw data for each figure in separate sheets, as well as a Pdf file with the uncropped versions of any gels or blots presented in this study.

Field-specific reporting

Please select the one below that is the best fit for your research. If you are not sure, read the appropriate sections before making your selection.

☒ Life sciences ☐ Behavioural & social sciences ☐ Ecological, evolutionary & environmental sciences

For a reference copy of the document with all sections, see nature.com/documents/nr-reporting-summary-flat.pdf

Life sciences study design

All studies must disclose on these points even when the disclosure is negative.

Sample size

Sample sizes for in vivo experiments were determined using the following criteria, as stated in the Materials and Methods section:
1) To determine sample size of the in vivo studies involving transgenic mice, a qualitative power analysis was performed using the formula: sample size = 2(Za/2+Za)2×P(1-P)/(p1-p2)2, where Za/2= type I error of 5% (=1.96, from Z table), Za= 0.842 at power 80% (from Z table), p1-p2= difference in proportion of events in two groups, P= pooled prevalence, i.e., (prevalence in case group [p1] + prevalence in the control group [p2])/2. Sample size values were adjusted for 10% attrition.
2) Sample size was calculated quantitatively using the formula: sample size = 2SD2×(Za/2+Za)2/d2, where SD= standard deviation (from previous studies (48)), Za/2= type I error of 5% (=1.96, from Z table), Za= 0.842 at power 80% (from Z table), d=effect size. All sample size values were adjusted for 10% attrition.
No statistical method was used to predetermine sample size for the in vitro experiments. Our sample size were as standard 3 independent experiments. For experiments where small differences were quantified, 4 independent experiments were conducted. The number of experiments are denoted in figure legends. These sample sizes were sufficient as the experiments were stable and reproducible. Untreated control cells (vehicle or cells transfected with siScr) were included as controls for each specific experiment.

Data exclusions

No data were excluded.

Replication

Reproducibility of the in vivo experiments related to tumor growth and kinetics (in transgenic mice) was confirmed in different litters coming from different parents and also after backcrossing. Experiments were repeated at least 3 times independently (unless otherwise stated) and were reproducible. Reproducibility of in vitro data was confirmed by the use of different melanoma cell lines and also by performing experiments at different passages (at a specific cell line level); all in vitro experiments were carried out at least in triplicates.

Randomization

Randomization was performed for all the in vivo experiments in which cells or drugs (Vehicle or FAKi) were injected. For the experiments other than in vivo studies, samples were randomized to the different treatment groups (cell type/ chemical treatment/knockdown conditions). Fields and cells acquired by microscopy for subsequent analysis were randomly selected.

Blinding

Blinding was not applied for the in vivo experiments dedicated to the analysis of tumor growth and kinetics or to the effect of FAKi on tumor growth as the researchers were aware of the genotype or of the experimental conditions. However, blinding was performed for all the

histological, pathological and immunostaining analyses performed by the pathologist on the tumor sections of the mice.

Reporting for specific materials, systems and methods

We require information from authors about some types of materials, experimental systems and methods used in many studies. Here, indicate whether each material, system or method listed is relevant to your study. If you are not sure if a list item applies to your research, read the appropriate section before selecting a response.

Materials & experimental systems

n/a	Involved in the study
☐	☒ Antibodies
☐	☒ Eukaryotic cell lines
☒	☐ Palaeontology and archaeology
☐	☒ Animals and other organisms
☒	☐ Human research participants
☒	☐ Clinical data
☒	☐ Dual use research of concern

Methods

n/a	Involved in the study
☒	☐ ChIP-seq
☒	☐ Flow cytometry
☒	☐ MRI-based neuroimaging

Antibodies

Antibodies used

Beta Tubulin (9F3), 1:5,000 (WB), Cell Signaling Technology (MA, USA), cat# 2128S, lot# 7, RRID: AB_823664;
Beta-Catenin (D10A8), 1:1,500 (WB), Cell Signaling Technology (MA, USA), cat# 8480T, lot# 5, RRID: AB_11127855;
Actin (AC-15), 1:40,000 (WB), Novus Biologicals (CO, USA), cat# NB600-501, lot# 64M4789V, RRID: AB_10077656;
AMBRA1 (G-6), 1:1,000 (WB), Santa Cruz Biotechnology (TX, USA), cat# sc-398204, lot# D0214;
Ambra1, 1:1,000 (WB), Merck-Millipore (MA, USA), cat# ABC131, lot# RA1207257, RRID: AB_2636939;
Atg7 (D12B11), 1:2,000 (WB), Cell Signaling Technology (MA, USA) cat# 8558S, lot# 2, RRID: AB_10831194;
CASP-3, 1:1,000 (WB), Cell Signaling Technology (MA, USA), cat# 9662S, lot# 15, RRID: AB_331439;
c-MYC (9E10), 1:500 (IP, WB), Santa Cruz Biotechnology (TX, USA), cat# sc-40, lot# F1917;
CDH1 (24E10), 1:1,000 (WB), Cell Signaling Technology (MA, USA), cat# 3195S, lot# 13, RRID: AB_2291471;
CDH2 (D4R1H), 1:1,000 (WB), Cell Signaling Technology (MA, USA), cat# 13116S, lot# 4, RRID: AB_2687616;
Cyclin D1 (SP4), 1:2,000 (WB), 1:400 (IHC), 1:500 (IF), Abcam (UK), cat# ab16663, lot# GR3256069-45, AB_443423;
FAK (D2R2E), 1:1,000 (WB), Cell Signaling Technology (MA, USA), cat# 13009S, lot# 1, RRID: AB_2798086;
HA (HA-7), 1:500 (IP), Merck-Millipore (MA, USA), cat# H3663, lot# 066M4837V, RRID: AB_262051;
Ki67 (SP6), 1:1,000 (IHC), 1:250 (IF), abcam (UK), cat# ab16667, lot# GR3255131-5, RRID: AB_302459;
LC3 (D11), 1:2,500 (WB), Cell Signaling Technology (MA, USA), cat# 3868S, lot# 13, RRID: AB_2137707;
PARP-1 (C-2-10), 1:1,000 (WB), Enzo Life Sciences (NY, USA), cat# BML-SA250, lot# 08021216, RRID: AB_2160745;
p-c-Myc-S62, 1:150 (IHC, IF), antibody developed and validated by the Prof. Sears laboratory;
pFAK-Y397 (D20B1), 1:1,000 (WB), 1:200 (IF), Cell Signaling Technology (MA, USA), cat# 8556S, lot# 1, RRID: AB_10891442;
pFAK-Y576, 1:750 (WB), 1:200 (IF), ThermoFisher Scientific (MA, USA), cat# PA5-104964, lot# VJ3099363, RRID: AB_2816437;
p62, 1:500 (IF), MBL (MA, USA); cat# PM045, lot# 021, RRID: AB_1279301
pSRC-Y416 (D49G4), 1:1,000 (WB), Cell Signaling Technology (MA, USA), cat# 6943S, lot# 4, RRID: AB_10013641;
S100, ready-to-use, Dako (CA, USA), cat# IS504, lot# 20076816, RRID: AB_2811056;
SNAI1 (C15D3), 1:1,500 (WB), Cell Signaling Technology (MA, USA), cat# 3879S, lot# 12, RRID: AB_2255011
SRC (32G6), 1:1,000 (WB), Cell Signaling Technology (MA, USA), cat# 2123S, lot# 5, RRID: AB_2106047
Vimentin (D21H3), 1:4,000 (WB), Cell Signaling Technology (MA, USA), cat# 5741S, lot# 6, RRID: AB_10695459
Vinculin (VIN-11-5), 1:4,000 (WB), Sigma-Aldrich (MO, USA), cat# V4505, lot# 074M4810V, RRID: AB_477617;
Vinculin (hVIN-1), 1:500 (IF), Sigma-Aldrich (MO, USA), cat# V9131, RRID: AB_477629.
Goat anti-Rabbit IgG (H+L) Cyanine5, 1:400 (IF), ThermoFisher Scientific (MA, USA), cat# A-10523, RRID: AB_2534032
Goat anti-Rabbit IgG (H+L) Alexa Fluor 568, 1:400 (IF), ThermoFisher Scientific (MA, USA), cat# A-11011, RRID:AB_143157
F(ab')2-Goat anti-Rabbit IgG (H+L) Cross-Adsorbed Secondary Antibody, Alexa Fluor 647, 1:400 (IF), ThermoFisher Scientific (MA, USA), cat# A-21246, RRID: AB_2535814

Validation

Beta Tubulin (9F3):
Validations: orthogonal (supplier).
Validation for application: WB.
Validation for species reactivity: Mus Musculus.
Source: https://www.citeab.com/antibodies/122895-2128-tubulin-9f3-rabbit-mab?utm_campaign=Widget+All+Citations&utm_medium=Widget&utm_source=Cell+Signaling+Technology&utm_term=Cell+Signaling+Technology

Beta-Catenin (D10A8):
Validations: orthogonal and biological strategies (supplier); knockdown and relative expression (literature).
Validation for application: WB.
Validation for species reactivity: Mus Musculus and Human.
Source: https://www.citeab.com/antibodies/125282-8480-catenin-d10a8-xp-rabbit-mab?des=a4fbd0e40f4e9c27

Actin (AC-15):
Validations: knockdown and relative expression (literature).
Validation for application: WB.
Validation for species reactivity: Human.
Source: https://www.citeab.com/antibodies/2304864-a5441-monoclonal-anti-actin?des=b3cdaf5d45ed4fea

AMBRA1 (G-6):
Validations: knockdown and relative expression. No signal detected on mice melanoma tumor samples.
Validation for application: WB.
Validation for species reactivity: Human.
Source: this study.

Ambra1:
Validations: in vivo knockout.
Validation for application: WB.
Validation for species reactivity: Mus Musculus.
Source: this study.

Atg7 (D12B11):
Validations: knockdown (supplier); relative expression (literature).
Validation for application: WB.
Validation for species reactivity: Human.
Sources: this study (knockdown) and https://www.citeab.com/antibodies/125378-8558-atg7-d12b11-rabbit-mab?des=d7b2e7436a03e55f

CASP-3:
Validations: relative expression (literature).
Validation for application: WB.
Validation for species reactivity: Mus Musculus and Human.
Source: https://www.citeab.com/antibodies/126298-9662-caspase-3-antibody?des=e0fe3e37068a473f

c-MYC (9E10):
Validation for application: IP and WB
Validation for species reactivity: Human
Sources: https://www.citeab.com/antibodies/2390803-sc-40-c-myc-antibody-9e10?des=3f53a719d0631ab5

CDH1 (24E10):
Validations: orthogonal and biological strategies (supplier); relative expression (literature).
Validation for application: WB.
Validation for species reactivity: Mus Musculus and Human.
Source: https://www.citeab.com/antibodies/124887-3195-e-cadherin-24e10-rabbit-mab?des=6bb7bedc235bfaf2

CDH2 (D4R1H):
Validations: orthogonal and biological strategies (supplier); relative expression (literature).
Validation for application: WB.
Validation for species reactivity: Mus Musculus and Human.
Source: https://www.citeab.com/antibodies/2043096-13116-n-cadherin-d4r1h-xp-rabbit-mab?des=5e167f19445ac601

Cyclin D1 (SP4):
Validations: Knockout (literature).
Validation for application: WB, IF, IHC.
Validation for species reactivity: Mus Musculus
Source: https://www.citeab.com/antibodies/725642-ab16663-anti-cyclin-d1-antibody-sp4?des=1fb6380993d227b1

FAK (D2R2E):
Validations: knockdown.
Validation for application: WB.
Validation for species reactivity: Mus Musculus and Human.
Source: this study.

HA (HA-7):
Validation for application: IP.
Validation for species reactivity: Human.
Source: https://www.citeab.com/antibodies/2304948-h3663-monoclonal-anti-ha?des=a866f1aed8aed5d2

Ki67 (SP6):
Validations: knockout (supplier).
Validation for application: IHC and IF.
Validation for species reactivity: Mus Musculus.
Source: https://www.citeab.com/antibodies/1896847-ab16667-anti-ki67-antibody-sp6?des=2b8a42a6b304c70b

LC3 (D11):
Validations: orthogonal, knockdown and biological strategies (supplier).
Validation for application: WB.
Validation for species reactivity: Mus Musculus and Human.
Source: https://www.citeab.com/antibodies/123587-3868-lc3b-d11-xp-rabbit-mab?des=03235a1407d07d91

PARP-1 (C-2-10):
No validation available.
Validation for application: WB.
Validation for species reactivity: Human.

Source: https://www.citeab.com/antibodies/308134-bml-sa250-parp-1-c-2-10?des=644a3e056ddfde74

p-c-Myc-S62
Antibody developed and validated by Prof. Sears laboratory
Validation for application: IHC and IF.
Validation for species reactivity: Mus Musculus

pFAK-Y397 (D20B1)
Validations: reduced immunofluorescence signal upon siFAK in SK-Mel-5 melanoma cells.
Source: this study.
Validation for application: WB and IF.
Validation for species reactivity: Mus Musculus and Human.

pFAK-Y576
Validation for application: WB and IF.
Validation for species reactivity: Mus Musculus and Human.
Source: https://www.citeab.com/antibodies/10483155-pa5-104964-phospho-fak-tyr576-polyclonal-antibody?des=2819f01f2e350f9c

p62
Validation for application: IF.
Validation for species reactivity: Mus Musculus.

pSRC-Y416 (D49G4):
Validations: biological strategies (supplier).
Validation for application: WB.
Validation for species reactivity: Mus Musculus and Human.
Source: https://www.citeab.com/antibodies/125939-6943-phospho-src-family-tyr416-d49g4-rabbit-mab?des=c82f1558cc242b9c

S100:
Validations: biological strategies (supplier). Specific to melanoma cells in lymph nodes metastases.
Validation for application: IHC and IF.
Validation for species reactivity: Mus Musculus.
Source: supplier, this study (specificity in melanoma metastases located at the lymph nodes).

SNAI1 (C15D3):
Validations: none available.
Validation for application: WB.
Validation for species reactivity: Mus Musculus and Human.
Source: https://www.citeab.com/antibodies/123609-3879-snail-c15d3-rabbit-mab?des=40ee877e46b12f5f

SRC (32G6):
Validations: none available.
Validation for application: WB.
Validation for species reactivity: Mus Musculus and Human.
Source: https://www.citeab.com/antibodies/122890-2123-src-32g6-rabbit-mab?des=62017efa01dfd985

Vimentin (D21H3):
Validations: orthogonal (supplier); relative expression (literature).
Validation for application: WB.
Validation for species reactivity: Mus Musculus and Human.
Source: https://www.citeab.com/antibodies/125713-5741-vimentin-d21h3-xp-rabbit-mab?des=68472d28d86833fe

Vinculin (VIN-11-5):
Validations: none available.
Validation for application: WB.
Validation for species reactivity: Human.
Source: https://www.citeab.com/antibodies/2304929-v4505-monoclonal-anti-vinculin?des=66248acc77460ee7

Vinculin (hVIN-1):
Validations: none available.
Validation for application: IF.
Validation for species reactivity: Human.
Source: https://www.citeab.com/antibodies/1038439-v9131-monoclonal-anti-vinculin?des=7fff5a6ee66a245d

Eukaryotic cell lines

Policy information about cell lines

Cell line source(s)	MeWo (ATCC® HTB-65™; RRID: CVCL_0445)<br>SK-Mel-2 (ATCC® HTB-68™; RRID: CVCL_0069)<br>SK-Mel-5 (ATCC® HTB-70™; RRID: CVCL_0527)<br>European Searchable Tumor Line Database (ESTDAB, https://www.ebi.ac.uk/ipd/estdab/; RRID:SCR_007746) were provided by Per Guldberg (Molecular Diagnostics Laboratory, Danish Cancer Society Research Center, Copenhagen, Denmark).
Authentication	Cell lines were authenticated by the provider with STR profiling.

Mycoplasma contamination	All the cell lines were routinely tested for Mycoplasma contamination at thawing, during subculture and prior to cryopreservation using a PCR-based detection method (eurofins Genomics, DE). All the cell lines used in this study were tested negative to Mycoplasma contamination.
Commonly misidentified lines (See ICLAC register)	None of the misidentified cell lines has been used in this study.

Animals and other organisms

Policy information about studies involving animals; ARRIVE guidelines recommended for reporting animal research

Laboratory animals	The laboratory animals used in this study belong to the species Mus Musculus, strain C57Bl/6, both male and female and experiments/treatments were performed in a time range spanning from postnatal day 1 up to postnatal day 350, depending on the experimental settings/conditions, as described in the manuscript.
Wild animals	The study did not involve wild animals.
Field-collected samples	The study did not involve samples collected from the field.
Ethics oversight	All animal care and mice experiments were performed in compliance with institutional guidelines and with protocols approved by the Danish animal experiments inspectorate (Dyreforsøgstilsynet, 2015-15-0201-00586 and 2020-15-0201-00578).

Note that full information on the approval of the study protocol must also be provided in the manuscript.
