# Supplementary material for: Loss of Ambra1 promotes melanoma growth and invasion
Source: Nat Commun. 2021 May 5;12:2550. doi: 10.1038/s41467-021-22772-2 (PMC8100102; doi:10.1038/s41467-021-22772-2)

Figure 2

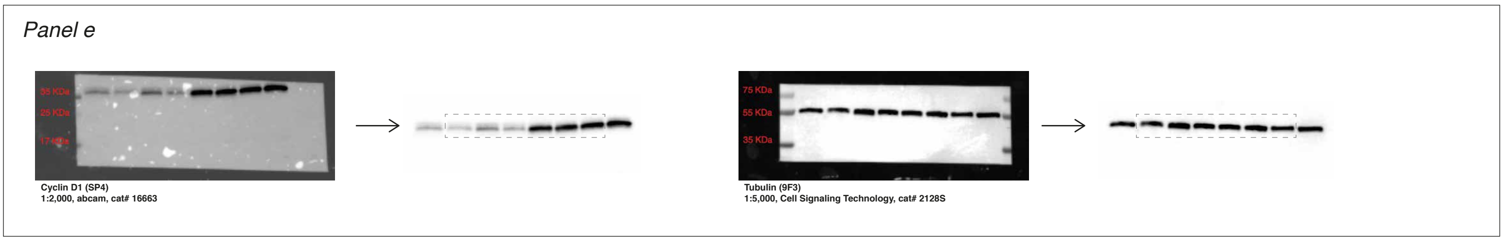

Figure 3

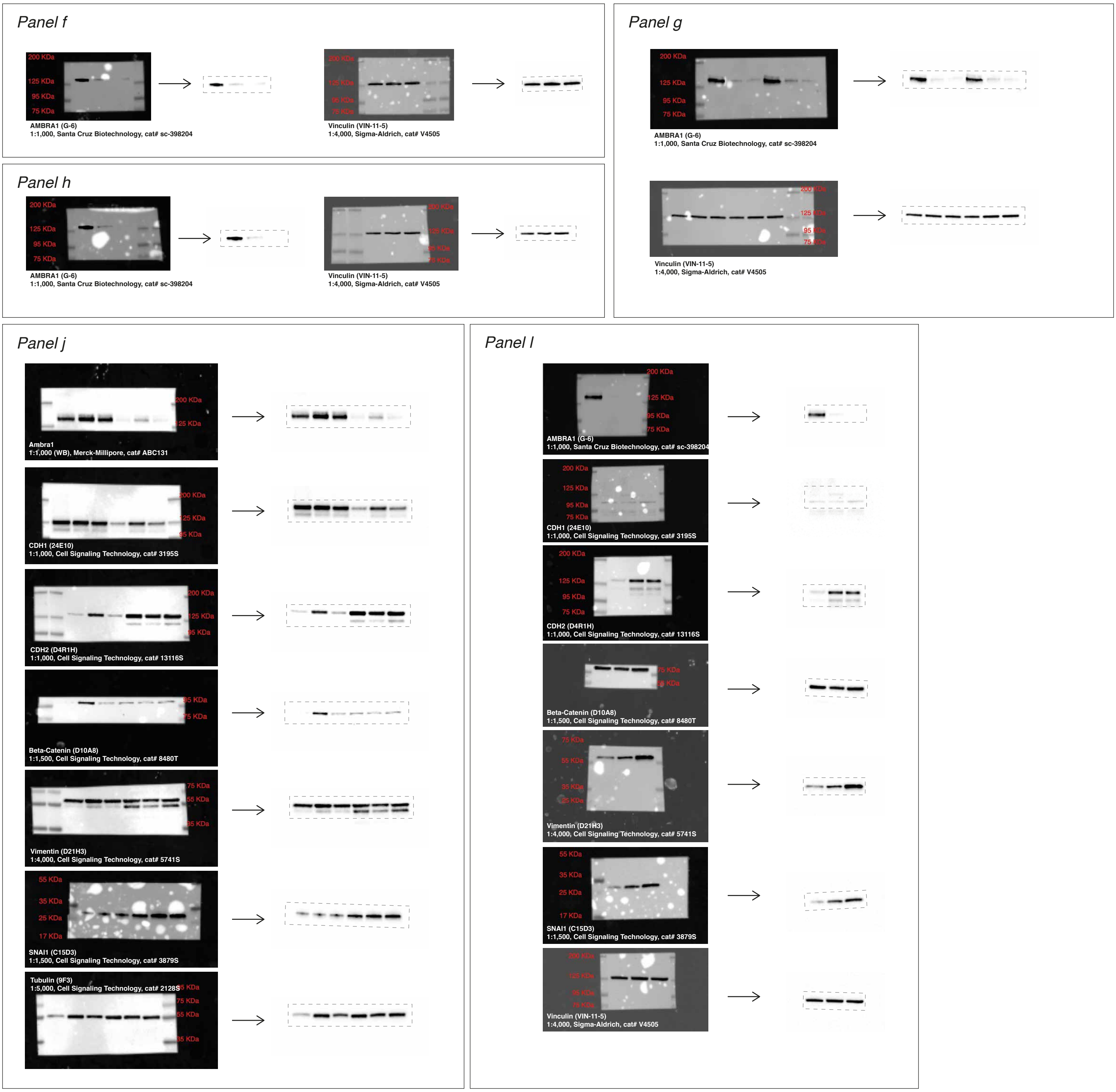

Figure 5

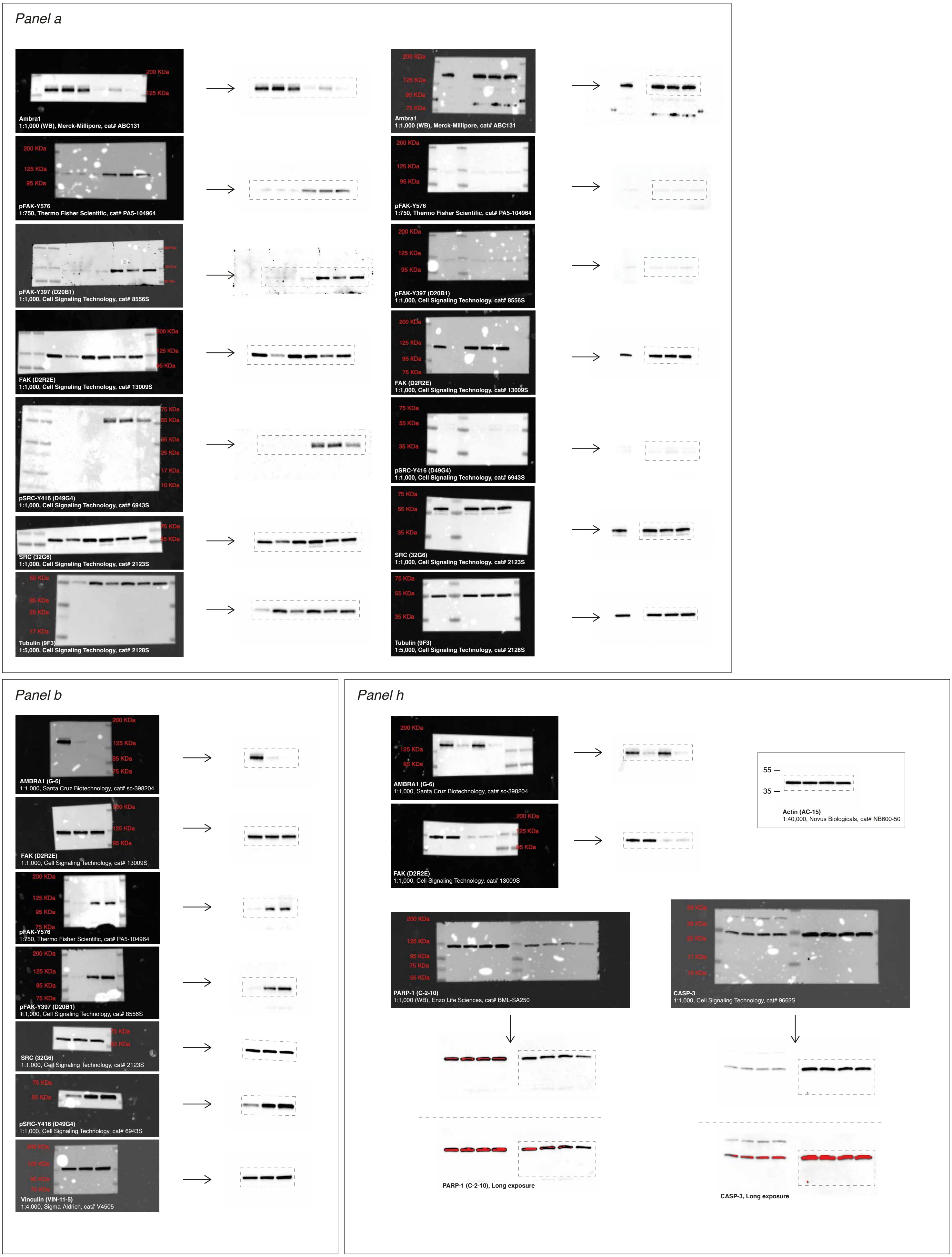
Panel a
Ambra1
1:1,000 (WB), Merck-Millipore, cat# ABC131
pFAK-Y576
1:750, Thermo Fisher Scientific, cat# PA5-104964
pFAK-Y397 (D20B1)
1:1,000, Cell Signaling Technology, cat# 8556S
FAK (D2R2E)
1:1,000, Cell Signaling Technology, cat# 13009S
pSRC-Y416 (D49G4)
1:1,000, Cell Signaling Technology, cat# 6943S
SRC (32G6)
1:1,000, Cell Signaling Technology, cat# 2123S
Tubulin (9F3)
1:5,000, Cell Signaling Technology, cat# 2128S
Panel b
AMBRA1 (G-6)
1:1,000, Santa Cruz Biotechnology, cat# sc-398204
FAK (D2R2E)
1:1,000, Cell Signaling Technology, cat# 13009S
pFAK-Y576
1:750, Thermo Fisher Scientific, cat# PA5-104964
pFAK-Y397 (D20B1)
1:1,000, Cell Signaling Technology, cat# 8556S
SRC (32G6)
1:1,000, Cell Signaling Technology, cat# 2123S
pSRC-Y416 (D49G4)
1:1,000, Cell Signaling Technology, cat# 6943S
Vinculin (VIN-11-5)
1:4,000, Sigma-Aldrich, cat# V4505
Panel h
AMBRA1 (G-6)
1:1,000, Santa Cruz Biotechnology, cat# sc-398204
FAK (D2R2E)
1:1,000, Cell Signaling Technology, cat# 13009S
55 —
35 —
Actin (AC-15)
1:40,000, Novus Biologicals, cat# NB600-50
PARP-1 (C-2-10)
1:1,000 (WB), Enzo Life Sciences, cat# BML-SA250
CASP-3
1:1,000, Cell Signaling Technology, cat# 9662S
PARP-1 (C-2-10), Long exposure
CASP-3, Long exposure

Figure 6

Panel a

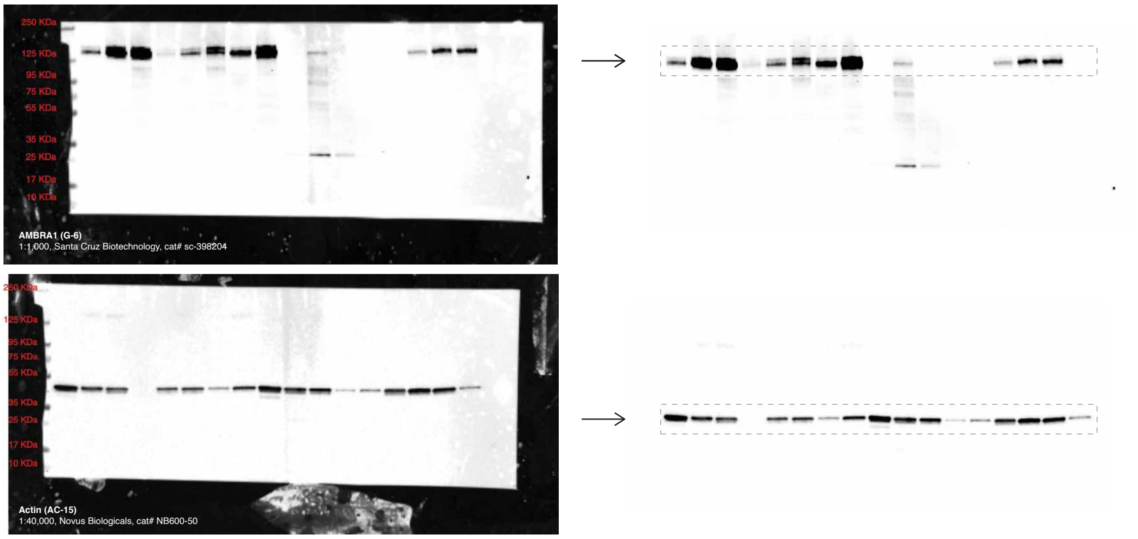

Figure S2

Panel a

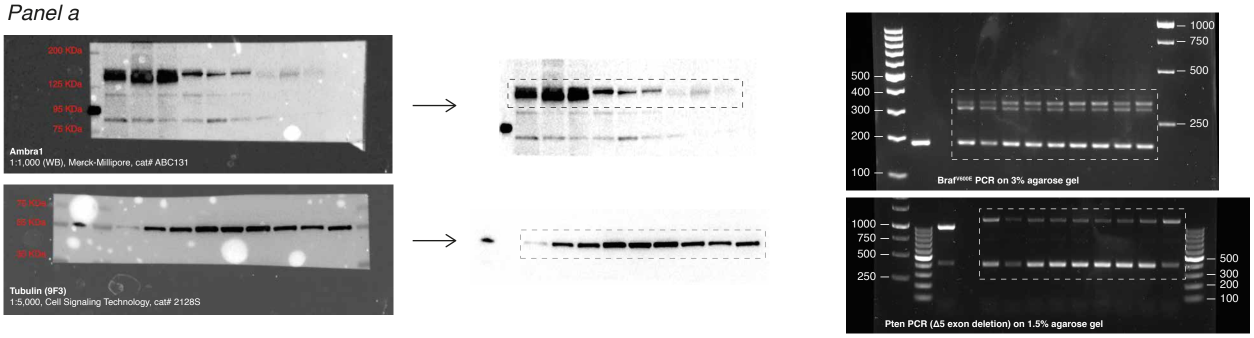

Panel m

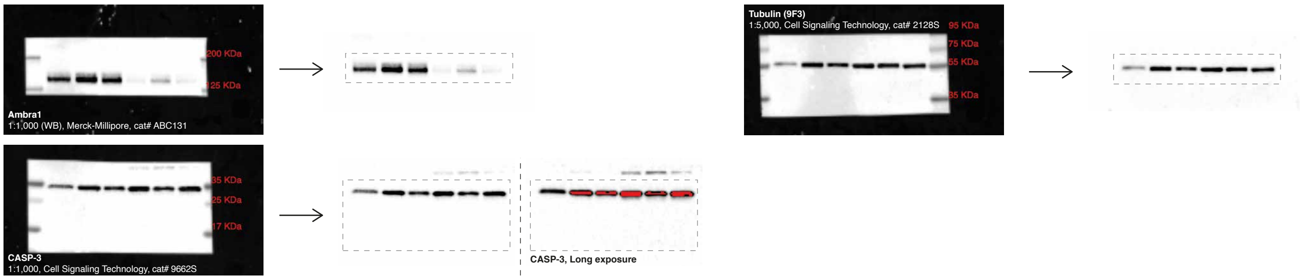

Figure S4

Panels l, m

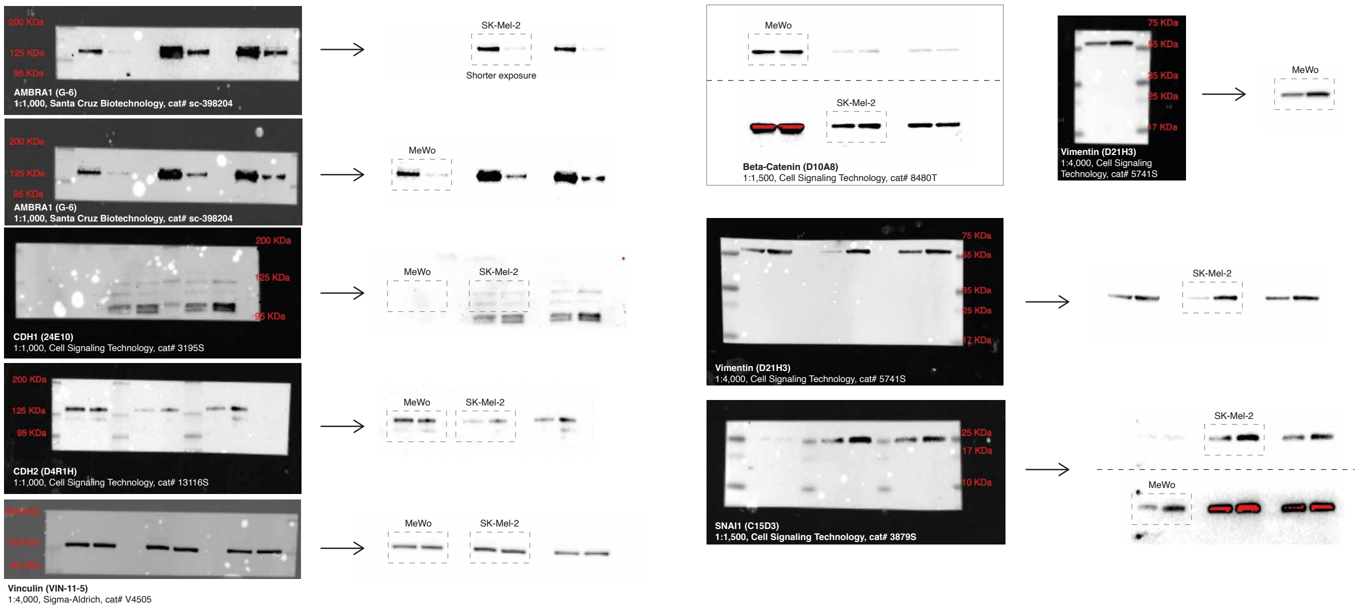

Figure S5

Panels c, d

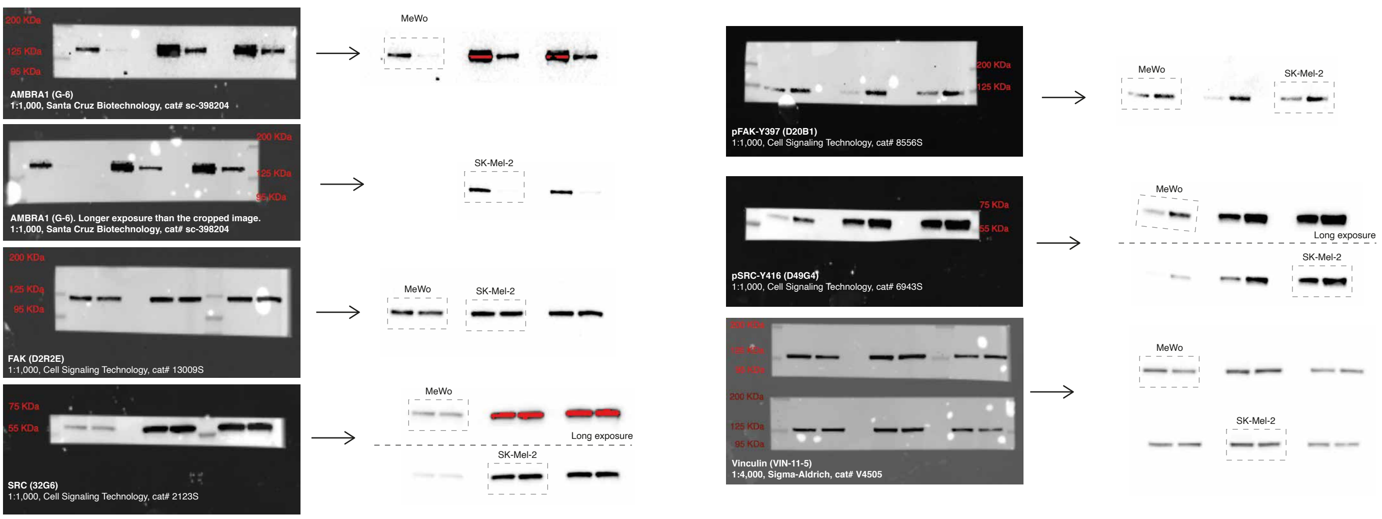

Panel j

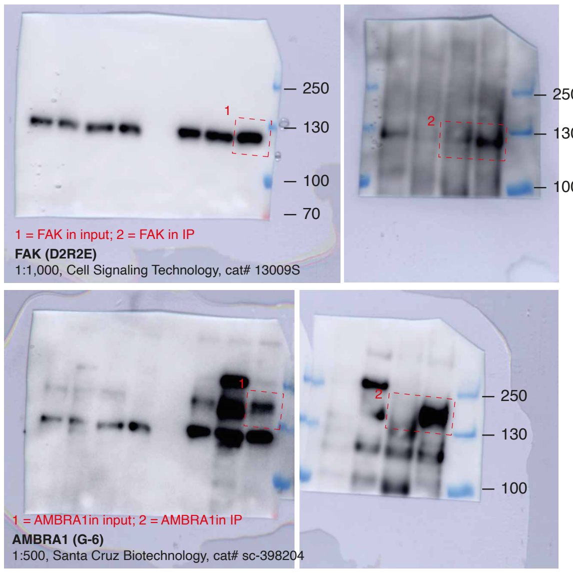

Panel k

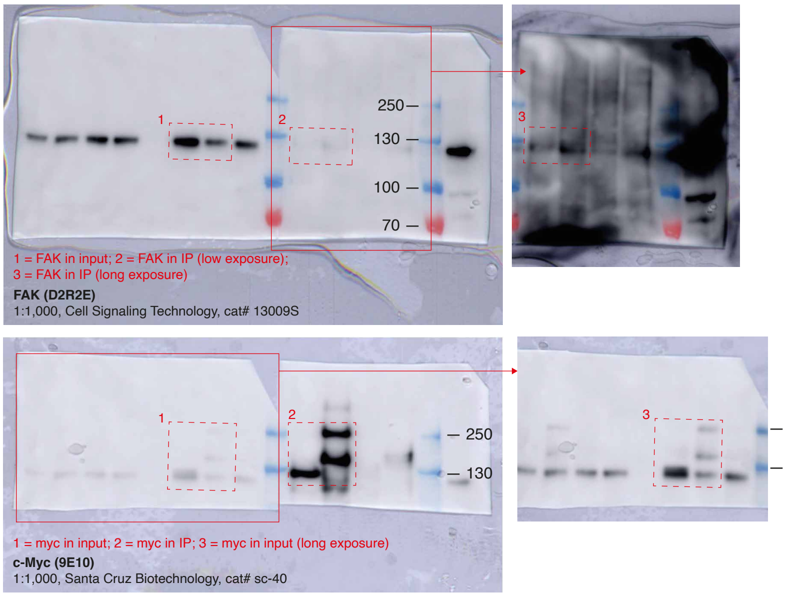

Figure S5

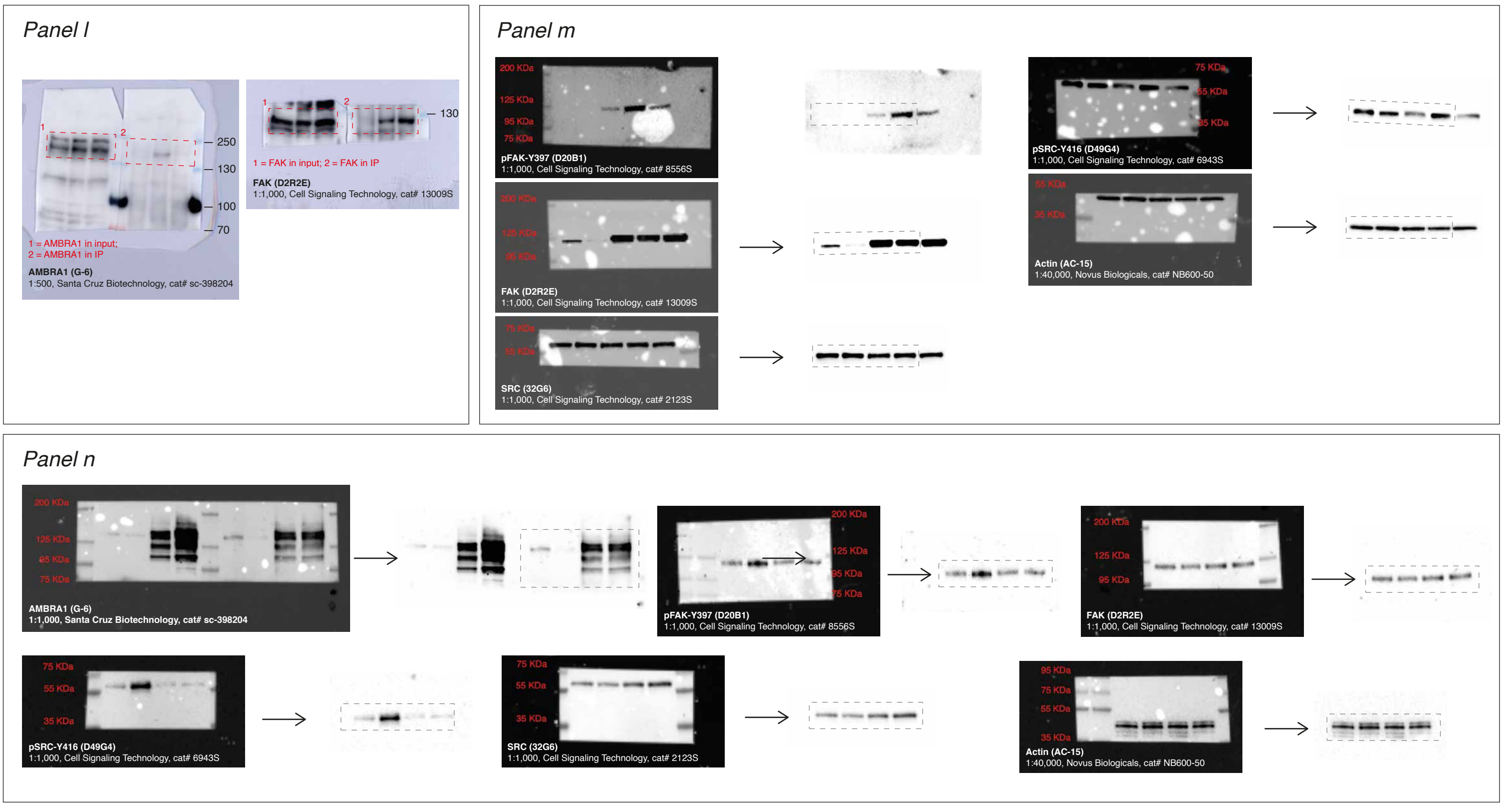

Figure S6

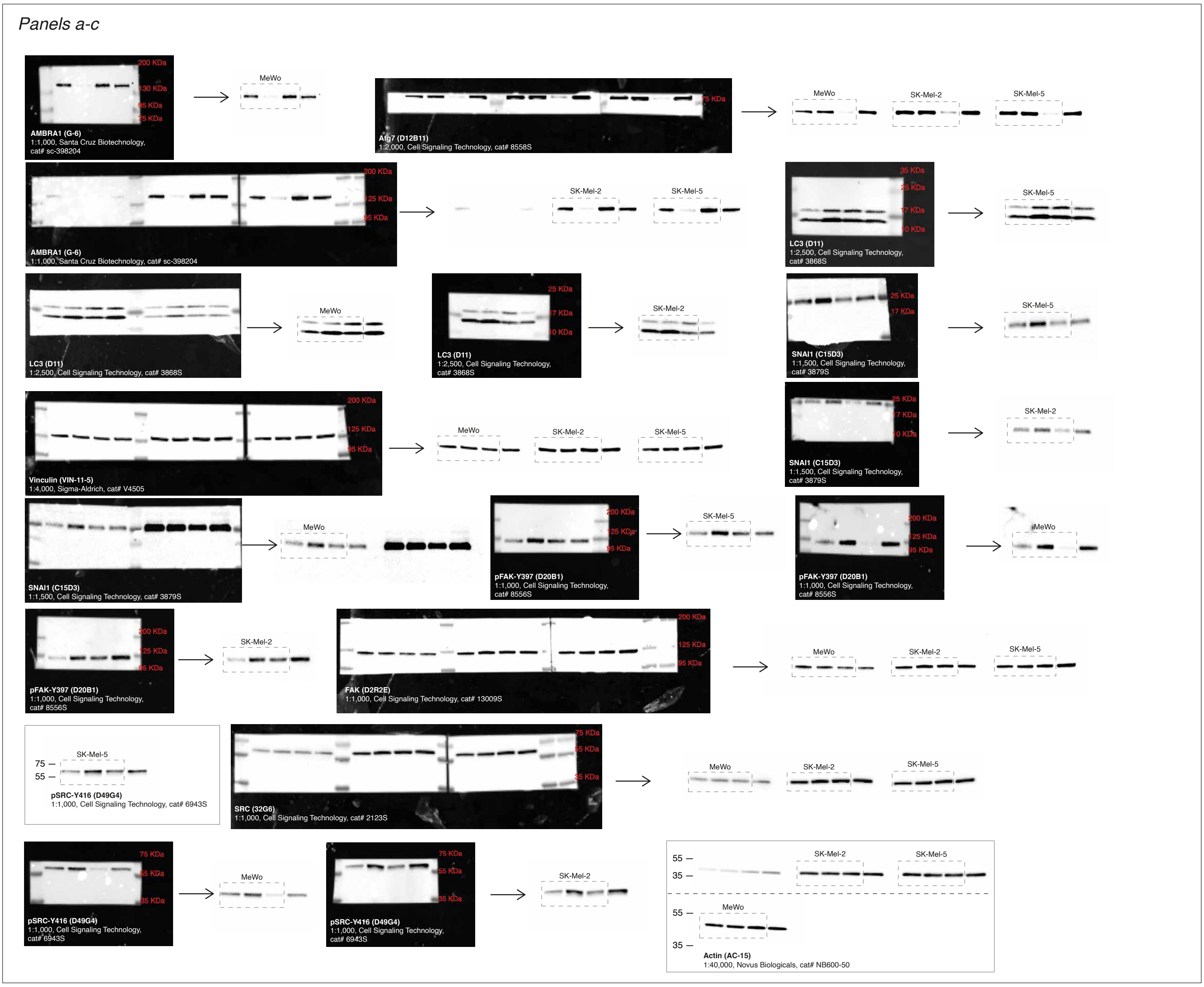

Figure S6

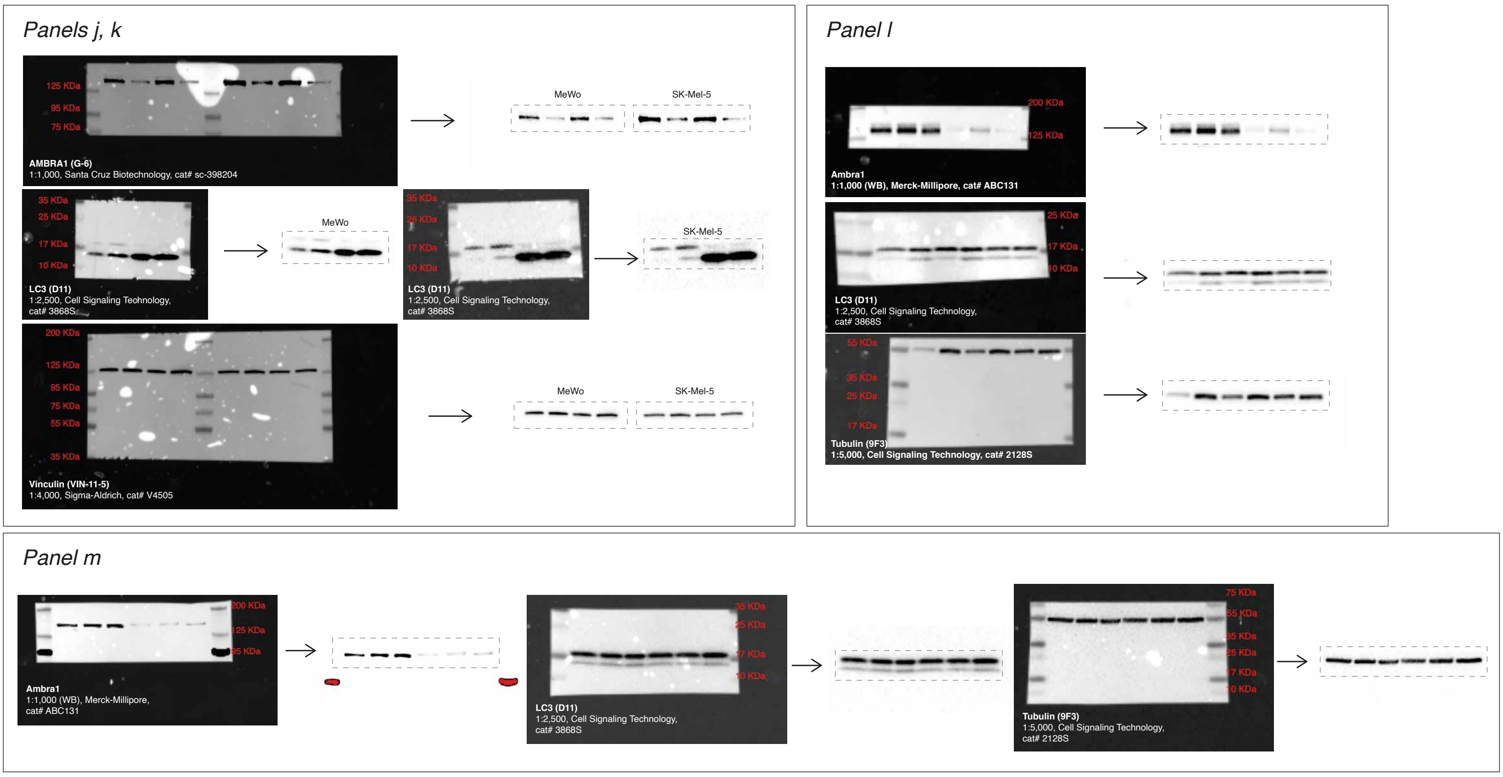

Figure S7

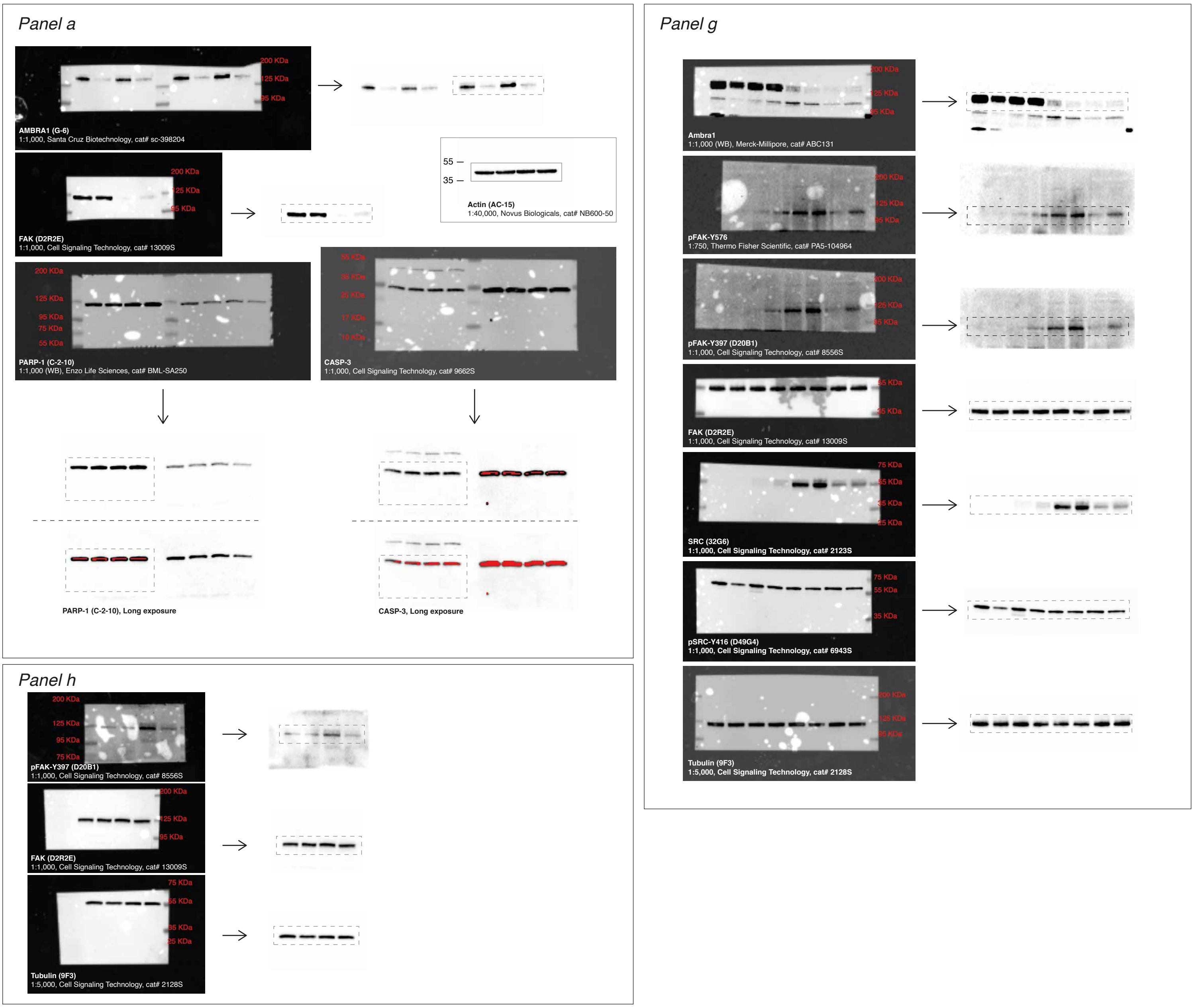

Figure S8

Panel b

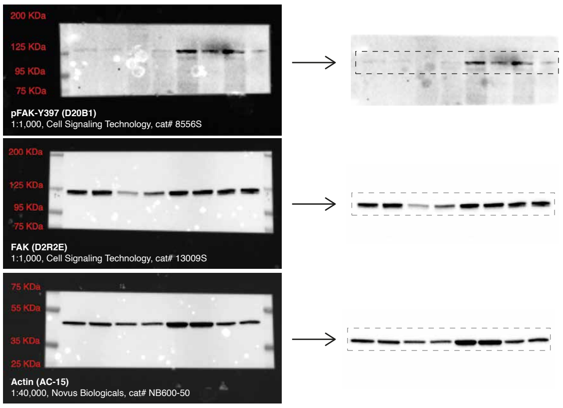

Supplement: Supplementary file 7 — Source Data [file 41467_2021_22772_MOESM7_ESM.zip › Source Data/Uncropped WB.pdf]
