# Supplementary material for: Loss of Ambra1 promotes melanoma growth and invasion
Source: Nat Commun. 2021 May 5;12:2550. doi: 10.1038/s41467-021-22772-2 (PMC8100102; doi:10.1038/s41467-021-22772-2)
Supplement: Supplementary file 2 — Peer Review File [file 41467_2021_22772_MOESM2_ESM.pdf]

Editorial Note: Parts of this Peer Review File have been redacted as indicated to maintain the confidentiality of unpublished data.

REVIEWER COMMENTS

Reviewer #1 (Remarks to the Author):

Loss of Ambra1 promotes melanoma growth and invasion

In this manuscript, Di Leo L et al investigated the role of AMBRA1 in melanoma tumorigenesis. The authors demonstrate that the absence of AMBRA1 promotes melanoma development and confers accelerated aggressiveness due to an increase in invasiveness-related gene signature. Moreover, the authors establish that the inhibition of FAK1, whose phosphorylation is induced by AMBRA1 deletion, can reduce the invasive phenotype mediated upon AMBRA1 absence, in an autophagy-independent manner. In all, the current study points to a novel role for AMBRA1 in melanoma growth and metastatic potential and highlights a possible therapeutic strategy where FAK1 inhibitors may be used in AMBRA1 low expressing tumors.

To substantiate authors conclusions and the overall impact of this study, the following changes are suggested.

Major

The authors suggest that AMBRA1 plays a role in different stages of melanoma, from the early phase of melanocytic proliferation in the non-malignant lesions to the metastatic potential of malignant melanoma. Yet, no direct evidence is provided in support of this model or claims. Authors should provide respective experiments which establish the importance of AMBRA1 in each of these phases.

Mechanistically, it is not clear how AMBRA1 contributes to the control of FAK1 phosphorylation. A clear study establishing the underlying mechanism is warranted. This is important in light of the fact that earlier studies already reported on AMBRA1 interaction with FAK1 and SRC. The implications of this mechanistic link for melanomagenesis, at its different stages, would be important.

Additional comments

Figure 1 Panels J,H, I
• Is the proliferative advantage, mediated by AMBRA1, relates to its role in autophagy? Since the possible link to autophagy was not assessed in the BPA-/- model (Fig s6d,e), it is important to substantiate possible link to autophagy seen here and in BA-/- mice, where AMBRA1 deletion elicits a different phenotype.
• Can the proliferative changes be linked with other reported AMBRA1 functions, such as the PP2A-cMYC signaling pathway?
• What happens to FAK1 phosphorylation status in the melanocytic nevi displayed in the BA-/- mice?
• Since the authors conclude that FAK1 has a central role in the AMBRA1-mediated tumorigenesis, these points need to be addressed.

Figure 3
Panel a – Gene ontology from RNAseq data highlights 4 pathways in BPA-/- mice, related to invasiveness. Are these the only pathways identified by the RNAseq? It would be interesting to know if other well-established AMBRA1 regulated genes / networks were affected in BPA-/- mice. A more comprehensive RNAseq/GO data should be presented.

Panel f - It is not clear if the two sets of images at the bottom labeled siAMBRA1 represent two different siRNA. If so, the lowest set of images does not show the effect in the gelatin invadopodia assay as shown in the middle panels, and the quantification of the right panel only shows the count for one set of siRNA. The result should be confirmed using another siRNA. Also, the efficiency of AMBRA1 depletion with different siRNA needs to be shown.

Panels g and h - Only one siRNA has been used (here as well as in other experiments). Use of additional siRNA should be included. Also, please confirm the degree of AMBRA1 KD.

Figure 4
Panel a – Only a representative picture of the iLNs is presented here. Authors should provide the quantification of the total amount and size of iLNs metastasis in BSA+/+ and BSA-/- mice.
Also, S100 staining suggest that the secondary tumors in the iLNs are bigger and more metastatic. Since the authors state in the next figures that the more invasive phenotype depends on FAK1 phosphorylation mediated by AMBRA1 deletion, it is important that authors establishes what happens to the FAK1 signaling in these secondary tumors?

Panel d - Here the author indicates that macroscopic lung metastases were not detected in the syngeneic model. Given the effect in vitro and the EMT-related gene set enrichment seen upon AMBRA1 deletion, how do the authors explain the lack of a visible metastatic effect in vivo at the time point analyzed (3 months)?

Figure 5
Panel c - the IF staining shows increase phosphorylation of FAK1 Y397. The set of images at the bottom panels do no show clear positive staining as claimed. Specify if the cells used in this experiment are the same cells used for panel b; the efficiency of the KD of AMBRA1 needs to be indicated.

Panel h - More siRNA for FAK1 (as well as for AMBRA1, as mentioned before) should be used to substantiate authors conclusions.

Panel k and l – The pharmacological inhibition of FAK1 in AMBRA1 deleted background shows a significant reduction of tumor growth. Since the syngeneic sBPA+/+ mice do not develop tumors (shown in figure 1 panel e), the model used here only can be applied to the syngeneic sBPA-/- mice.

- The inhibitor of FAK1 should be tested in both genetic settings to compare the effect of FAK1 inhibitor in the absence/presence of AMBRA1. If possible, the GEMM mouse model, where tumor development occurs in both genetic backgrounds, should be tested.
- In addition to the pharmacological inhibition model, a syngeneic model of BPA+/+/BPA-/- mice derived cells or human melanoma cells manipulated ex vivo for the KD of FAK1 should be used.

[REDACTED]

Panel i and j - The FAK1 KD rescues the phenotype in wound healing and migration assay supporting a central role for FAK1 in the AMBRA1-mediated invasive phenotype in vitro. The same should be demonstrated to occur in vivo.

Does the genetic or pharmacological inhibition of FAK1 reduce the effect of AMBRA1 deletion in lymph node metastasis and/or in the melanoma cell homing in the lung?

Reviewer #2 (Remarks to the Author):

Di Leo and colleagues report on Autophagy/Beclin1 regulator 1 (Ambra1) as a tumor suppressor in melanoma. Their study builds on previous findings that Ambra1, a scaffold protein, is predicted to be a tumor suppressor, with deficiency impairing autophagy and inducing neuroepithelial hyperplasia. Additionally, their group previously reported that ambra1 controls cell proliferation by regulating c-Myc stability by an interaction with protein phosphatase 2A. In the current study they use an established mouse melanoma model (oncogenic Braf and Pten-null) crossed for Ambra1 +/+, +/- and -/- to support their conclusions on tumor suppressor activity of Ambra1. Their study is comprehensive with significant new data. Several sections, however, as indicated below, need additional clarity.

1. An overall concern for significance and impact is linking the tumor suppressor function of Ambra1 that the authors convincingly show with experimental conditions and genetic landscape data with patient-derived metastatic melanoma – not clonal melanoma cell lines . As the authors state in the Introduction, loss or decreased abundance of Ambra1 is not associated with any stage/feature of melanoma.

2. If the BP mouse is a model for melanoma, why were no tumors observed, including with the syngeneic model? Is it merely because of the shorter time analyzed with Ambra1-nulls?

3. The EMT-like phenotype with Ambra1 silencing in Fig. 3 is interesting and well presented. For immunoblots, quantification of the indicated n=3 should be included and kD for indicated proteins should be checked. Snail should run at ~ 30 not 25 kD.

4. For data in Fig. 4, the conclusion that Ambra1 depletion promotes melanoma metastasis should be more circumspect based on tail-vein delivery bypassing tissue invasion.

5. Increased FAK autophosphorylation with Ambra1 deficiency is clearly presented in Fig. 5; however, this does not indicate increased FAK kinase activity, which is more accurately determined by FAK-pY576. This determination should be included to conclude that FAK activity is increased. As indicated for Fig. 3, quantification data of multiple immunoblots should be included. Also, confirming in vivo efficacy of FAKi by immunoblotting for FAK auto or kinase domain phosphorylation would be stronger. Finally, based on previous findings linking Ambra1 to FAK, what new mechanistic understanding do these findings reveal?

6. The authors should address a general concern on the complex mouse genotypes. Distinct floxed loci can recombine with different efficiencies, and sometimes with little concordance in which cells undergo recombination (https://pubmed.ncbi.nlm.nih.gov/23441020/). Hence, confirming that recombination of the Braf, Pten , and Ambra1 alleles occurs in the same cells, or with equal efficiencies is prudent.

Reviewer #3 (Remarks to the Author):

In this study, the authors provided data for a role of an autophagy gene Ambra1 (Autophagy/Beclin1 regulator 1) in melanoma development. They show that conditional deletion of Ambra1 increased tumor development in the Braf/Pten-mutated mouse models of melanoma (i.e. BPA-/- mice), and decreased their overall survival. They also show that Ambra1 deletion increased tumor cell motility, invasion and EMT to promote melanoma progression and metastasis. Lastly, they show that Ambra1 deletion increased FAK activation and signaling in melanoma cells. Overall, the study lacks mechanistic depth and insights at this stage. FAK inhibitors have been widely reported to inhibit tumor growth in a variety of models. Furthermore, some of the phenotypic assays appear confounding. Specific comments are as follows:

1) The fact that Ambra1 deletion leads to increased proliferation in pre-malignant lesions (Figs. 1h-i) but decreased proliferation in tumors (Figs. 2a-b) is perplexing. Without more convincing evidence to indicate a plausible reason for this phenomenon, it is difficult to draw any meaningful conclusion.

2) The efficiency of Ambra1 deletion should be verified by western blot and IHC for the protein in conditional KO mice models in Fig. 1.

3) The transcriptomic analysis of bulk tumors in figure 3 could be due to changes in stromal composition (i.e. fibroblasts) rather than changes in tumor cell gene expression. Increased expression of ECM related genes is typical of fibroblasts. This may explain the more subtle changes in gene expression when the experiments were performed in human melanoma cell lines (Figs. 3k-l).

[REDACTED]

5) It seems rather surprising that FAK silencing does not affect wound closure (fig. 5i) nor cell migration (fig. 5j). How does this compare to published observations?

6) Figures 5k-l need to be carried out with BPA +/+ cells as well. If FAK inhibitor treatment could decrease tumor growth similarly in both BPA+/+ and BPA-/- cells, then this would undermine the interpretation that FAK is important predominantly in AMBRA deficient tumors.

7) Also, pharmacological inhibitors in general have off-target effects. Thus, genetic experiments are needed to complement the FAK inhibitor experiments.

Point-by-Point Rebuttal Letter

<u>Reviewer #1</u>:

> ***In this manuscript, Di Leo L et al investigated the role of AMBRA1 in melanoma tumorigenesis. The authors demonstrate that the absence of AMBRA1 promotes melanoma development and confers accelerated aggressiveness due to an increase in invasiveness-related gene signature. Moreover, the authors establish that the inhibition of FAK1, whose phosphorylation is induced by AMBRA1 deletion, can reduce the invasive phenotype mediated upon AMBRA1 absence, in an autophagy-independent manner. In all, the current study points to a novel role for AMBRA1 in melanoma growth and metastatic potential and highlights a possible therapeutic strategy where FAK1 inhibitors may be used in AMBRA1 low expressing tumors.***
> ***To substantiate authors conclusions and the overall impact of this study, the following changes are suggested.***
> ***Major***
> ***The authors suggest that AMBRA1 plays a role in different stages of melanoma, from the early phase of melanocytic proliferation in the non-malignant lesions to the metastatic potential of malignant melanoma. Yet, no direct evidence is provided in support of this model or claims. Authors should provide respective experiments which establish the importance of AMBRA1 in each of these phases.***

We thank the Reviewer for this comment and do agree with her/him that some aspects related to the role of *Ambra1* in the different stages of melanoma development – spanning from non-malignant lesions to accumulation of melanocytic lesions and, further, to tumor progression – were not accurately investigated. Accordingly, we performed new experiments, which are hereinafter described.

1) ***Mechanistically, it is not clear how AMBRA1 contributes to the control of FAK1 phosphorylation. A clear study establishing the underlying mechanism is warranted. This is important in light of the fact that earlier studies already reported on AMBRA1 interaction with FAK1 and SRC. The implications of this mechanistic link for melanomagenesis, at its different stages, would be important.***

We thank the Reviewer for this comment. As requested, we have now performed new experiments aimed at understanding the molecular mechanism(s) underlying AMBRA1-mediated FAK1 activation. In the present version of the manuscript, we have included co-immunoprecipitation analyses providing clear evidence that FAK1 and AMBRA1 do interact in melanoma cells, both endogenously (**Fig. S5j**) and upon expression of a plasmid coding for a tagged form of AMBRA1 (AMBRA1-myc) (**Fig. S5k**). Moreover, we demonstrate that this interaction is functional to the hyperphosphorylation of FAK1 and SRC, as well as to the invasive phenotype observed in melanoma cells. Indeed, similarly to what observed upon AMBRA1 silencing, the re-expression of a mutant form of FAK1 (FAK1$^{P876A/P882A}$), which was previously reported – and herein confirmed by co-immunoprecipitation – to be unable to bind AMBRA1 (**Fig. S5l**, *Schoenherr C. et al., Elife*

2017), caused the activation of FAK1 signaling (**Fig. S5m**) and enhanced the migratory ability of melanoma cells (**Fig. S7d**).

Based on the evidence arguing for an increased phosphorylation of c-Myc in *Ambra1*-deficient melanoma (see point 3), we also investigated if PP2A activity could contribute to FAK1 activation. Indeed, previous studies reported that PP2A, whose activity is enhanced by Ambra1 (Cianfanelli *et al., Nat Cell Biol*, 2015), regulates focal adhesions (Young, Rita *et al., Int J Cancer*, 2002; Janssens *et al., J Cell Sci*, 2016). Our new results indicate that the re-expression of the mutant form of AMBRA1, which is uncapable of binding PP2A (*AMBRA1^{PXP}, Cianfanelli V. et al., Nat Cell Biol 2015*; *Becher J. et al., Dev Cell 2018*), did not affect the phosphorylation state of both FAK1 and SRC (**Fig. S5n**), therefore excluding any involvement of AMBRA1-related PP2A activity in the regulation of FAK1 phosphorylation upon AMBRA1 depletion.

Additional comments

2) ***Figure 1 Panels J,H, I***
   • Is the proliferative advantage, mediated by AMBRA1, relates to its role in autophagy? Since the possible link to autophagy was not assessed in the BPA-/- model (Fig s6d,e), it is important to substantiate possible link to autophagy seen here and in BA-/- mice, where AMBRA1 deletion elicits a different phenotype.

We thank the Reviewer for this additional relevant comment. In the revised manuscript, we demonstrate that the autophagy-related function of Ambra1 is not responsible for the hyperproliferative phenotype observed in non-malignant lesions of Braf mutated mice (**Fig. S2f, j**), thus confirming previous evidence obtained in BPA mice.

As also requested by Reviewer 3 (point 1), we have looked into this aspect in greater depth and better defined the proliferative phenotype of early non-malignant lesions and late metastatic melanoma lacking *Ambra1*. We kindly invite the Reviewer to refer to that point for further clarifications.

3) ***Can the proliferative changes be linked with other reported AMBRA1 functions, such as the PP2A-cMYC signaling pathway?***

We thank the Reviewer for this very nice suggestion. As she/he hypothesized, we have observed increased phosphorylation of c-Myc (p-c-Myc-S62) in absence of *Ambra1* in both *Braf/Pten*-mutated melanoma (**Fig. 2f, g**) and (though not significant) in *Braf*-mutated melanocytes (**Fig. S2f, h**). This evidence allows speculating that Ambra1 deficiency is linked to c-Myc phosphorylation in our settings and, very likely, underlies the hyperproliferative phenotype observed in the different phases of melanomagenesis.

Concerning any other possible mechanism(s) linking Ambra1 to hyperproliferative phenotype, it is worthwhile to mention that our laboratory has very recently discovered that Ambra1 regulates Cyclin D1 stability by interacting with the E3 ligase DDB1/cullin4, thus controlling cell cycle progression (*Maiani E. et al., recently accepted in Nature*). This finding opens up to a new mechanism of regulation of cell proliferation mediated by Ambra1, which has been found highly conserved both in physiological and pathological conditions. We therefore sought to investigate if this mechanism was also activated in our models of melanoma. In this new version of the

manuscript, we provide evidence that Cyclin D1 significantly accumulates upon *Ambra1* deficiency, *i.e.*, in $BPA^{-/-}$ tumors (**Fig. 2e-g**) and in $BA^{-/-}$ non-malignant lesions (**Fig. S2f, g**), arguing for the hyperproliferative phenotype observed in Ambra1-deficient mice being also related to Cyclin D1 stability.

4) ***What happens to FAK1 phosphorylation status in the melanocytic nevi displayed in the BA-/- mice?***

We thank the Reviewer for this comment. As mentioned above, we observed that phospho-FAK1 is slightly increased also in $BA^{-/-}$ mice (**Fig. S2f, i**), implying a role for FAK1 signaling in the hyperpigmentation and hyperproliferation observed upon *Ambra1* deficiency.

5) ***Since the authors conclude that FAK1 has a central role in the AMBRA1-mediated tumorigenesis, these points need to be addressed.***

We are very grateful to the Reviewer for her/his comments. We hope that the new lines of evidence will convince her/him in regards to our conclusions.

Figure 3

6) ***Panel a – Gene ontology from RNAseq data highlights 4 pathways in BPA-/- mice, related to invasiveness. Are these the only pathways identified by the RNAseq? It would be interesting to know if other well-established AMBRA1 regulated genes / networks were affected in BPA-/- mice. A more comprehensive RNAseq/GO data should be presented.***

We apologize with the Reviewer for not having included the complete gene ontology analysis in the previous manuscript. Now, we have added a supplementary table (**Table S2**) in which we show the full list of activated pathways in $BPA^{-/-}$ *versus* $BPA^{+/+}$ tumors obtained with different tools (GO, KEGG, Panther, Reactome, Wikipathways, *adj pvalue <0.01*). Precisely, the pathways shown in **Fig. 3a** have been selected as they are found at the top of the list (lower *adj pvalue*) and appeared in more than one list (GO, KEGG, Panther, Reactome, Wikipathways). As evident in **Table S2**, GO terms related to the well-established AMBRA1-regulated genes/networks (*e.g.* autophagy, mitophagy) did not appear within our list. Data in Table S2 confirm the results reported in **Fig S6**, indicating that the autophagy function of AMBRA1 is unrelated to its novel role in regulating migration and invasiveness in melanoma.

7) ***Panel f - It is not clear if the two sets of images at the bottom labeled siAMBRA1 represent two different siRNA. If so, the lowest set of images does not show the effect in the gelatin invadopodia assay as shown in the middle panels, and the quantification of the right panel only shows the count for one set of siRNA. The result should be confirmed using another siRNA. Also, the efficiency of AMBRA1 depletion with different siRNA needs to be shown. Panels g and h - Only one siRNA has been used (here as well as in other experiments). Use of additional siRNA should be included. Also, please confirm the degree of AMBRA1 KD.***

We understand the Reviewer’s concern and thank her/him for giving us the opportunity to better explain the methodological aspects of the experiment shown in **Fig. 3f**. The middle and bottom

panels are just two representative images of one set of AMBRA1 siRNA. However, in line with Reviewer's request, we have now performed additional experiments using another siRNA targeting (the 5'-UTR region of) *AMBRA1* (*siAMBRA#2*) for both the gelatin invadopodia assay (**Fig. 3f**), the wound healing assay (**Fig. 3g**), and the transwell migration assay (**Fig. 3h**). For all these assays, we have also performed *ad hoc* quantifications, which are now shown in an updated version of the graphs, as well as in WB analyses, which confirm the degree of AMBRA1 silencing (**Fig. 3f-h**).

Figure 4

8) ***Panel a – Only a representative picture of the iLNs is presented here. Authors should provide the quantification of the total amount and size of iLNs metastasis in BSA+/+ and BSA-/- mice.***

We thank the Reviewer for her/his comment. Indeed, the mouse model applied in this manuscript has long and widely been used by the melanoma community (*Dankort et al., Nat Genet 2009; Damsky et al., Cancer Cell 2011; Hooijkaas et al., Am J Pathol 2012; Claps et al., Cell Rep 2016*). Therefore, in order to evaluate metastases and quantify the metastatic burden in LNs, we have carefully followed the already set and commonly-accepted methodology. It is based on calculating the ratio between the area of pigmented S100^{+} cells (tumor metastases) on the area of the entire LN, since the macroscopic analysis of the entire LN would not allow a correct quantification of metastases. Specifically in our case, we have quantified 3 sections for each lymph node which were representative of the whole organ; then, values obtained were summed in order to obtain a single data point for each mouse (please, refer to the "*Immunohistochemistry (IHC) and Immunofluorescence (IF) on Tissue Sections*" paragraph of "*Supplementary Material and Methods*" for further details). We hope the Reviewer will agree with us and be convinced by this quantification on LNs sections. However, in order to meet the request of the Reviewer, we have also measured the area of each LN section and calculated the average LN area in each group (**Fig. 4b**). Doing so, we have also been able to quantitatively assess that $BPA^{-/-}$ LNs are larger in size than $BPA^{+/+}$ LNs.

9) ***Also, S100 staining suggest that the secondary tumors in the iLNs are bigger and more metastatic. Since the authors state in the next figures that the more invasive phenotype depends on FAK1 phosphorylation mediated by AMBRA1 deletion, it is important that authors establishe what happens to the FAK1 signaling in these secondary tumors?***

We thank the Reviewer for this suggestion. As requested, we have now performed and included this analysis in the revised version of the manuscript (**Fig. S5e**). Interestingly, we have observed that FAK1 phosphorylation is maintained at higher levels in the LNs of $BPA^{-/-}$ mice (**Fig. S5e**).

10) ***Panel d - Here the author indicates that macroscopic lung metastases were not detected in the syngeneic model. Given the effect in vitro and the EMT-related gene set enrichment seen upon AMBRA1 deletion, how do the authors explain the lack of a visible metastatic effect in vivo at the time point analyzed (3 months)?***

We thank the Reviewer for this comment. In line with what explained in the manuscript, the syngeneic mice injected in the tail vein with $BPA^{+/+}$- and $BPA^{-/-}$-derived cells did not form macroscopically visible nodules in the lungs. This could be due to the fact that the number of cells

injected (0.25×10^6) was not sufficient for the development of macroscopic nodules in the timeframe of our experiment (3 months).
As regards the transgenic mouse model, the Danish legislation and common practice caring about animal wellness in mouse experiments impose us to sacrifice the mice when tumors reach a size that is defined as [$(L+W)/2 \geq 12$]*, where L=length and W=width. Therefore, this strict rule does not enable us to follow tumor progression for a time that is long enough for melanoma to spread to distal organs, such as lungs. For this practical reason, we were able to only observe metastases in the LNs of our transgenic mice.
We apologize for the mistake we made in the Materials and Methods section where we wrongly reported [$(L \times W)/2 \geq 12$].

Figure 5

11) ***Panel c - the IF staining shows increase phosphorylation of FAK1 Y397. The set of images at the bottom panels do no show clear positive staining as claimed. Specify if the cells used in this experiment are the same cells used for panel b; the efficiency of the KD of AMBRA1 needs to be indicated.***

We thank the Reviewer for giving us the opportunity to clarify this aspect. The set of images shown in the bottom panels of the previous **Fig. 5c** indicates two representative images of the same *siAMBRA1*. However, as already requested by the Reviewer, and in order to strengthen our findings, we have repeated the same experiment and the related analyses on focal adhesions by using another siRNA targeting the 5'-UTR region of *AMBRA1* (*siAMBRA#2*). The IF panels, as well as the quantifications provided in **Fig. S5f-i**, have been updated accordingly. Moreover, the WB shown in **Fig. 5b** can be considered as a *bona fide* control for the AMBRA1 silencing.

12) ***Panel h - More siRNA for FAK1 (as well as for AMBRA1, as mentioned before) should be used to substantiate authors conclusions.***

We thank the Reviewer for this comment. With the aim to dissect the molecular mechanism of AMBRA1-mediated FAK1 activation, we have used another set of custom-made *siRNAs* for FAK1 (*siFAK#2*) (**Fig. S5m** and **S7d**). Also, we would like to point out that the *siFAK1* used in the experiments shown in **Fig. 5h-j** and in **Fig. S7a-c** is a pool of *siRNAs* (MISSION® esiRNA human PTK2, Sigma-Aldrich, MO, USA; cat# EHU077321), meaning that it contains *siRNAs* targeting different regions of FAK1.

13) ***Panel k and l – The pharmacological inhibition of FAK1 in AMBRA1 deleted background shows a significant reduction of tumor growth. Since the syngeneic sBPA+/+ mice do not develop tumors (shown in figure 1 panel e), the model used here only can be applied to the syngeneic sBPA-/- mice.***
The inhibitor of FAK1 should be tested in both genetic settings to compare the effect of FAK1 inhibitor in the absence/presence of AMBRA1. If possible, the GEMM mouse model, where tumor development occurs in both genetic backgrounds, should be tested.

We thank the Reviewer for this suggestion, understand her/his concern on the use of syngeneic models and do agree with her/him that the GEMM model would be a valuable alternative for this

purpose. Therefore, we attempted to administer *FAKi* in our transgenic mice. However, soon after injection, both Ambra1-WT and -KO mice showed several signs of distress (*e.g.* weight loss). In compliance with the local regulations, the mice had to be euthanized and the experiment could not be completed. This response/reaction to *FAKi* was ascribed to the age of the mice when the treatment started (approx. 5 weeks of age, as established by our procedure for tamoxifen induction) and is untimely compared to the age of the mice used in the syngeneic model (11-12 weeks). Therefore, we apologize for not being able to address this specific Reviewer's request.
Likely, the number of cells injected, as well as the timeframe of the experiment (in accordance to the strict Danish legislation on this matter), did not allow us to observe any tumors developing in mice injected with $BPA^{+/+}$-derived cells. To overcome these limitations, we have performed an additional syngeneic experiment in which mice have been injected with 5×10^6 cells in order to ensure development of tumors in both genetic backgrounds and within a shorter timeframe. Similar to the experiment described in **Fig. 5k**, mice received a daily dose of *FAKi* when tumors were measurable (details are reported in *Material and Methods*). As expected, the kinetics of tumor growth was faster in these mice than in those injected with 2×10^6 cells. Therefore, tumors reached earlier the maximum tolerated size, and drugs administration could be performed for only 10 days after tumors were first measured (*versus* 21 days of the experiment shown in new **Fig. 5l**). In addition – and unfortunately – 3 out of the 5 *FAKi*-treated $sBPA^{-/-}$ mice had to be euthanized after 5 days of treatment as a consequence of tumor ulceration. Nevertheless, and of the highest importance, the kinetics of tumor growth in $sBPA^{-/-}$ mice in this second syngeneic experiment (**Fig. S7e**) fully recapitulate those previously obtained (**Fig. 5l**). More importantly, in this new experiment, we observed that tumor growth in $sBPA^{-/-}$ mice *i*) was faster and *ii*) significantly affected by *FAKi* treatment than in $sBPA^{+/+}$ mice, where, indeed, no relevant reduction of the kinetics (**Fig. S7e, f**) and tumor weight (**Fig. 5m)** were observed. This evidence strongly supports that the activation of FAK1 signaling is specific to *Ambra1*-deficient tumors.

14) In addition to the pharmacological inhibition model, a syngeneic model of BPA+/+/BPA-/- mice derived cells or human melanoma cells manipulated ex vivo for the KD of FAK1 should be used.

We agree in principle with the Reviewer that dissecting the effects of Fak1 genetic manipulation *in vivo* would be interesting. Indeed, in agreement with her/his request, we have started to manipulate murine YUMM1.7 and 1.1 melanoma cells (*Meeth K. et al., Pigment Cell Melanoma Res 2016*) either with *shAmbra1* or *shFak1,* alone or in combination. However, unfortunately, unforeseen technical issues, exacerbated by COVID-19 pandemics, have negatively affected the proceeding of this experiment. However, in order to assess this point of the Reviewer, we did not give up and decided to perform experiments taking advantage of the panel of human melanoma cells that we have shown to naturally express low levels of AMBRA1, as shown in **Fig. 6a, b**. In detail, we have carried out a dose-response assay with *FAKi* in 15 melanoma cell lines for 24 h. Our results revealed a striking correlation between AMBRA1 protein expression levels and sensitivity to *FAKi* (**Fig. 6j** and **S8a**), *i.e.* cells that express low levels of AMBRA1 are more sensitive to *FAKi*. Moreover, we also observed that non-lethal doses of *FAKi* (**Fig. S8b, c**) are capable of rescuing the invasive/migratory capacity in a selection of AMBRA1-low-expressing human melanoma cell lines

(**Fig. 6k; S8d**). This evidence, although not *in vivo*, further proves that AMBRA1-induced FAK1 signaling underlies the invasive phenotype of melanoma cells and highlights FAK1 as a possible new therapeutic target in AMBRA1-low melanomas. We hope that the Reviewer will understand our difficulties, and share the view that this evidence has been fully corroborated by our alternative approach.

[REDACTED]

16) ***Panel i and j - The FAK1 KD rescues the phenotype in wound healing and migration assay supporting a central role for FAK1 in the AMBRA1-mediated invasive phenotype in vitro. The same should be demonstrated to occur in vivo.***
Does the genetic or pharmacological inhibition of FAK1 reduce the effect of AMBRA1 deletion in lymph node metastasis and/or in the melanoma cell homing in the lung?

We agree with this Reviewer on the fact that dissecting the *in vivo* effect of Fak1 inhibition in Ambra1-mediated invasive phenotype would be of great interest. Unfortunately, for the reasons explained in point 13, we could not perform this specific experiment *in vivo* in our transgenic model. Also, we collected and performed immunostaining for S100 in the LNs from syngeneic mice treated with *FAKi* to look for tumor metastases, but we were not able to observe any metastasis in these samples, most likely as a consequence of the short timeframe of the experiment, imposed by the current European legislation on animal welfare.

Nevertheless, in order to address Reviewer's concerns, we investigated the effect of *FAKi* treatment both in the human melanoma cells with different expression levels of AMBRA1 (already discussed

in point 14) and *ex vivo*, in the primary murine melanoma cells derived from BPA tumors (Bdmc$^{+/+}$ and Bdmc$^{-/-}$). In particular, non-lethal doses of *FAKi* were able to rescue both the migrative and invasive phenotypes specifically in Bdmc$^{-/-}$ cells (**Fig. 5n, o** and **S7h**), suggesting that *FAKi* may potentially affect the metastatic capacity also *in vivo*. Again, we hope the Reviewer will appreciate our effort, despite obvious difficulties.

<u>Reviewer #2</u>:

> ***Di Leo and colleagues report on Autophagy/Beclin1 regulator 1 (Ambra1) as a tumor suppressor in melanoma. Their study builds on previous findings that Ambra1, a scaffold protein, is predicted to be a tumor suppressor, with deficiency impairing autophagy and inducing neuroepithelial hyperplasia. Additionally, their group previously reported that ambra1 controls cell proliferation by regulating c-Myc stability by an interaction with protein phosphatase 2A. In the current study they use an established mouse melanoma model (oncogenic Braf and Pten-null) crossed for Ambra1 +/+, +/- and -/- to support their conclusions on tumor suppressor activity of Ambra1. Their study is comprehensive with significant new data. Several sections, however, as indicated below, need additional clarity.***

We are honored that the Reviewer appreciated the novelty of our study and apologize with her/him for missing information. In the revision of the manuscript, we have now tried to clarify all the Reviewer's requests.

1) ***An overall concern for significance and impact is linking the tumor suppressor function of Ambra1 that the authors convincingly show with experimental conditions and genetic landscape data with patient-derived metastatic melanoma – not clonal melanoma cell lines. As the authors state in the Introduction, loss or decreased abundance of Ambra1 is not associated with any stage/feature of melanoma.***

We thank the Reviewer for her/his comment, which certainly deserves some explanations. In the introduction we wanted to underline that *no studies have been yet carried out in order to associate the levels of AMBRA1 expression with disease progression in melanoma patients*. We apologize for this lack of clarity and therefore reformulated the sentence in the revised version of the manuscript as follows: «…*Although this can argue for AMBRA1 being prognostic of melanoma outcome, a direct role of AMBRA1 in this disease has not yet been investigated*…».

Moreover, in the context of another study, we are currently evaluating the possibility that AMBRA1 could represent a prognostic biomarker in human melanoma. This study is in close collaboration with Prof. Lovat's group at Newcastle University. Briefly, the semi-quantitative automated immunohistochemical (IHC) analysis of tumoral AMBRA1 expression is being determined in a powered cohort of primary AJCC stage I/II melanomas and correlated with 12-year clinical follow-up data. However, since this analysis is part of a joint ongoing clinical study with Lovat's group, we would prefer not to include this analysis in the present manuscript. Nevertheless, we have now analyzed and included transcriptomic data from primary melanoma samples of the Leeds Melanoma Cohort (LMC) in the revised version of this manuscript. This new dataset comprises a larger number of melanoma patients (n=703), only including primary melanoma, with respect to TCGA-SKCM dataset (n=473). Interestingly, GSEA analyses revealed that AMBRA1_Low subgroups are significantly enriched for ECM, EMT and FA genes, as well as for genes linked to invasiveness in melanoma, also in this dataset (**Fig. 6i**), further supporting our hypothesis.

2) ***If the BP mouse is a model for melanoma, why were no tumors observed, including with the syngeneic model? Is it merely because of the shorter time analyzed with Ambra1-nulls?***

We thank the Reviewer for her/his comment Admittedly, we assume that the timeframe used in our approach is too short, mostly in relation to the low number of *BPA*$^{+/+}$ cells injected in C57BL6 recipient mice. To confirm this, we injected more cells (*i.e.*, 5×10^{6}) and, as a consequence, tumor growth could be observed also in *sBPA*$^{+/+}$ syngeneic mice (**Fig. 5m** and **S7e, f).** For a more extensive and detailed explanation of these new results, we kindly ask the Reviewer to refer to our response to issue 13 of Reviewer 1.

3) ***The EMT-like phenotype with Ambra1 silencing in Fig. 3 is interesting and well presented. For immunoblots, quantification of the indicated n=3 should be included and kD for indicated proteins should be checked. Snail should run at ~ 30 not 25 kD.***

We thank the Reviewer for this suggestion and apologize for the missing information. We have now included the quantification of the WB from **Fig. 3** in the revised version of the manuscript (please refer to densitometry in **Fig. S4h** for the bulk tumors shown in **Fig. 3j**, and in **Fig. S4j** for melanoma cells upon two different *siAMBRA1* shown in **Fig. 3l**). We also apologize for mistaking the molecular weight of Snai1, which has now been corrected. Also, as for bulk tumors, we have now provided a new and more appropriate WB analysis for Snai1 (please see new **Fig. 3j**).

4) ***For data in Fig. 4, the conclusion that Ambra1 depletion promotes melanoma metastasis should be more circumspect based on tail-vein delivery bypassing tissue invasion.***

We thank the Reviewer for this comment and agree with her/him that we probably did not stress enough the message in the old version of the manuscript. Among the different steps of the metastatic process is the dissemination of tumor cells to the LNs. In **Fig. 4** of the new version of our manuscript, we indeed provide evidence of tissue invasion by melanoma cells, this being more prominent in *BPA*$^{-/-}$ (**Fig 4c, d**), suggesting that *Ambra1* depletion does promote melanoma metastases. However – as explained in our response to point 10 of Reviewer 1 – we were not able to detect distal (*i.e.*, lung) metastases in our transgenic mice. Therefore, in order to demonstrate that *Ambra1*-deficient cells were also able to colonize distant organs by means of the blood stream, melanoma cells were IV-injected and the homing capability of melanoma cells to the lungs analyzed (**Fig. 4e-h**).

5) ***Increased FAK autophosphorylation with Ambra1 deficiency is clearly presented in Fig. 5; however, this does not indicate increased FAK kinase activity, which is more accurately determined by FAK-pY576. This determination should be included to conclude that FAK activity is increased.***

We thank the Reviewer for this very nice suggestion. As requested, we have performed new analyses and included new results in **Fig. 5a** and **S7g** (bulk tumors) and **Fig. 5b** (melanoma cells upon two different *siAMBRA1*).

6) ***As indicated for Fig. 3, quantification data of multiple immunoblots should be included.***

We apologize to the Reviewer for the missing information. As requested, the WB analyses shown in **Fig. 5a** (bulk tumors) and in **Fig. 5b** (melanoma cells upon two different *siAMBRA1*) have now been quantified and are provided in **Fig. S5a** and **S5b**, respectively.

7) ***Also, confirming in vivo efficacy of FAKi by immunoblotting for FAK auto or kinase domain phosphorylation would be stronger.***

We thank the Reviewer for this suggestion. Accordingly, we have now analyzed the *in vivo* efficacy of *FAKi* on either domain in protein extracts of bulk tumors. These results are now provided in **Fig. S7g**.

8) ***Finally, based on previous findings linking Ambra1 to FAK, what new mechanistic understanding do these findings reveal?***

We thank the Reviewer for this comment and – as also requested by Reviewer 1 – we performed new experiments to understand the molecular mechanism(s) underlying AMBRA1-mediated FAK1 activation. In the present version, we provide evidence that FAK1 and AMBRA1 do interact in melanoma cells and that this interaction is functional to the invasive phenotype of melanoma cells (**Fig. S5j-m** and **S7d**). Indeed, by introducing the mutant form of FAK1, which is unable to bind AMBRA1 (*Schoenherr C. et al., Elife 2017*) in FAK1-silenced cells, we were able to confirm the abrogation of AMBRA1-FAK1 interaction (**Fig. S5l**), as well as to mimic the phenotype observed upon AMBRA1 deletion (*e.g.*, hyper-phosphorylation of FAK1 and SRC associated with higher migration and invasion capability of melanoma cells; please see new **Fig. S5m** and **S7d**). For a more extensive and detailed explanation of these new results, we kindly ask the Reviewer to refer to our response to point 1 of Reviewer 1.

9) ***The authors should address a general concern on the complex mouse genotypes. Distinct floxed loci can recombine with different efficiencies, and sometimes with little concordance in which cells undergo recombination (https://pubmed.ncbi.nlm.nih.gov/23441020/). Hence, confirming that recombination of the Braf, Pten , and Ambra1 alleles occurs in the same cells, or with equal efficiencies is prudent.***

We thank the Reviewer for this valuable comment. We do recognize the issue related to the efficiency of Cre-mediated recombination and have therefore tested it for *Braf*, *Pten* and Ambra1 in $BPA^{+/+}$, $BPA^{+/-}$ and $BPA^{-/-}$ tumors. The new panel *a* of **Fig. S2 (S2a)** shows the presence of *Braf* and *Pten floxed alleles* ($Braf^{CA}$ and Δ*5 Pten*) as well as the expression levels of Ambra1 in all three genotypes. We hope that these data will convince the Reviewer that Cre-mediated recombination efficiently works in our system.

<u>Reviewer #3</u>:

> ***In this study, the authors provided data for a role of an autophagy gene Ambra1 (Autophagy/Beclin1 regulator 1) in melanoma development. They show that conditional deletion of Ambra1 increased tumor development in the Braf/Pten-mutated mouse models of melanoma (i.e. BPA-/- mice), and decreased their overall survival. They also show that Ambra1 deletion increased tumor cell motility, invasion and EMT to promote melanoma progression and metastasis. Lastly, they show that Ambra1 deletion increased FAK activation and signaling in melanoma cells. Overall, the study lacks mechanistic depth and insights at this stage. FAK inhibitors have been widely reported to inhibit tumor growth in a variety of models. Furthermore, some of the phenotypic assays appear confounding. Specific comments are as follows:***

1) ***The fact that Ambra1 deletion leads to increased proliferation in pre-malignant lesions (Figs. 1h-i) but decreased proliferation in tumors (Figs. 2a-b) is perplexing. Without more convincing evidence to indicate a plausible reason for this phenomenon, it is difficult to draw any meaningful conclusion.***

We thank the Reviewer for this comment and do agree with her/him that we did not thoroughly explain the phenomenon in the previous version of our manuscript. We show that the proliferative rate of WT tumors is slightly higher than in the KO after 42 days from tumor induction, when KO tumors are clearly bigger and more invasive than the WT (**Figs. 1a-d, 2a-b, 4a-d**). To better investigate this phenomenon, we have now performed Ki67 immunostaining in $BPA^{+/+}$ and $BPA^{-/-}$ tumors at same Breslow thickness, both immediately after tumors were measurable (≤ 2 mm) and when tumors reached the maximum allowed size imposed by the Danish legislation (≥ 4 mm). By means of this analysis, we have been able to observe that the rate of proliferation is always similar between $BPA^{+/+}$ and $BPA^{-/-}$ at same Breslow thickness (**Fig. 2c, d**). Moreover, at more advanced state (≥ 4 mm), we observed an overall decrease in the number of Ki67^{+} cells, independently on the genotype analyzed (**Fig. 2c, d**), indicating that proliferation decreases when tumors reach the maximum size. Therefore, what makes the difference in the absence of *Ambra1* is the shorter timeframe needed to reach the maximum allowed tumor size and enhanced invasive state of melanoma. Taken together, these lines of evidence allow us to conclude that KO tumors have a proliferative advantage which we have now demonstrated to be associated with the function of Ambra1 on c-Myc and Cyclin D1, as described in the reply to <u>point 3, Reviewer 1</u> (**Fig. 2e-g**). Additionally, we have also performed histological analyses of iLNs of $BPA^{+/+}$ mice at their maximum tolerated tumor size, which was reached later than the KO (59 days). By means of this approach, we revealed a trend in the pigmented areas (*i.e.* metastases) which was comparable – though lower – with the observations made in $BPA^{-/-}$ mice at 42 days (**Fig. 4a-d**). Most importantly, the invasive advantage of $BPA^{-/-}$ tumors was in this case ascribed to the activation of Fak1 signaling. In fact, the phospho-activation of Fak1 (as well of Src) was specific to $BPA^{-/-}$ and did not change in protein extracts of $BPA^{+/+}$ bulk tumors at 42 and 59 days (**Fig. 5a, Fig. S7g**).

Overall, these findings strongly support that *Ambra1* deletion does confer both a proliferative and an invasive advantage to melanoma. We really hope that these new sets of data fully clarify the

Reviewer’s concern and prove her/him that *Ambra1* can be considered a *bona fide* melanoma suppressor.

2) ***The efficiency of Ambra1 deletion should be verified by western blot and IHC for the protein in conditional KO mice models in Fig. 1.***

We thank the Reviewer for this suggestion and do apologize for the inaccuracy. We have now included a WB analysis for Ambra1 protein levels in $BPA^{+/+}$, $BPA^{+/-}$ and $BPA^{-/-}$ tumors, as shown in **Fig. S2a**. Unfortunately, and after several tests, we have not been able to identify a good-quality and/or commercially-available antibody raised against Ambra1 for IHC/IF in murine tissues. This prevented us from performing the requested analyses on either BPA or BA mice. We hope the Reviewer will understand this omission, but -in the absence of a reliable antibody, this remains a task that we cannot perform.

3) ***The transcriptomic analysis of bulk tumors in figure 3 could be due to changes in stromal composition (i.e. fibroblasts) rather than changes in tumor cell gene expression. Increased expression of ECM related genes is typical of fibroblasts. This may explain the more subtle changes in gene expression when the experiments were performed in human melanoma cell lines (Figs. 3k-l).***

We understand the Reviewer’s concerns, and we do agree with her/him that the changes in gene expression observed in $BPA^{-/-}$ tumors could also be due to the stromal composition of the tumor. However, the evidence that increased expression of EMT markers occurs both in human melanoma cells silenced for AMBRA1 (**Fig. 3k**; **Fig. S4k**) – which was also accompanied by increase digestion of the matrix (**Fig. 3f**) – and in Ambra1 KO tumor-derived primary cells (**Fig. S4i**) argue for melanoma cells mostly contributing to the invasive phenotype.

[REDACTED]

[REDACTED]

5) ***It seems rather surprising that FAK silencing does not affect wound closure (fig. 5i) nor cell migration (fig. 5j). How does this compare to published observations?***

We thank the Reviewer for the keen observation and apologize for the inaccuracy. We have now carefully revised our raw data and statistical analyses and updated the quantifications shown in **Fig. 5i, j** and **Fig. S7b, c** accordingly. Moreover, we would also like to direct the attention of the Reviewer to new data that we have produced in the context of other experiments requested during this revision, which required the use of another *siRNA* against FAK1 (*siFAK#2*) in human melanoma cells. In these conditions as well, we could appreciate a reduction in cell migration upon FAK1 silencing (please, see new **Fig. S7d**.).

6) ***Figures 5k-l need to be carried out with BPA +/+ cells as well. If FAK inhibitor treatment could decrease tumor growth similarly in both BPA+/+ and BPA-/- cells, then this would undermine the interpretation that FAK is important predominantly in AMBRA deficient tumors.***

We thank the Reviewer for this suggestion and agree with her/him that the response to *FAKi* must be assessed also in syngeneic mice injected with *BPA*$^{+/+}$-derived cells. Therefore, we have performed an additional syngeneic experiment in which we injected $5{\times}10^6$ cells/mouse, with the aim of ensuring tumor development in both genetic backgrounds. Mice received a daily dose of *FAKi* when tumors were measurable but, due to a higher number of injected cells, tumors reached maximum tolerated size much faster than in the syngeneic model with $2{\times}10^6$ cells/mouse. For this reason, drugs administration was performed for only 10 days. Nevertheless, the effect of *FAKi* on

tumor growth was clearly specific to *sBPA*$^{-/-}$ mice, as no significant difference in the kinetics of tumor growth was observed in *FAKi- versus Vehicle*-treated *sBPA*$^{+/+}$ mice (**Fig. S7e, f**). These results strongly support the relevance of Fak1 signaling axis specifically in *Ambra1*-deficient tumors. For a more extensive and detailed explanation of this section, we kindly ask the Reviewer to refer to our response to point 13 of Reviewer 1, who raised similar concerns about this experiment.

7) ***Also, pharmacological inhibitors in general have off-target effects. Thus, genetic experiments are needed to complement the FAK inhibitor experiments.***

We thank the Reviewer for this comment. As mentioned above (our response to Reviewer 1, point 14), though fully understanding the wish of the Reviewer(s) and agreeing on the relevance of these results in supporting our claims, we have not been able to perform this very experiment mostly as a consequence of unforeseen technical issues and delays related to the COVID-19 pandemics.
However, as an attempt to address the request from the Reviewers, we have performed experiments taking advantage of the panel of human melanoma cells that we have shown to naturally express low levels of AMBRA1 (shown in **Fig. 6a, b**). Our dose-response curves upon *FAKi* treatment in 15 melanoma cell lines, revealed that AMBRA1 low-expressing melanoma cells are more sensitive to *FAKi* (**Fig. 6j**) and that non-lethal doses of *FAKi* (**Fig. S8b, c**) could specifically rescue the invasive/migratory capacity of a selection of these AMBRA1-low-expressing human melanoma cell lines (**Fig. 6k; Fig. S8d**). This evidence, though not properly addressing the Reviewer's concern, further supports the hypothesis that AMBRA1-induced FAK1 signaling underlies the invasive phenotype of melanoma cells, and points out FAK1 as a possible new therapeutic target in AMBRA1-low melanomas.

REVIEWERS' COMMENTS

Reviewer #1 (Remarks to the Author):

authors have addressed most of the points raised by the reviewers and the revised manuscript is notably improved.

Reviewer #2 (Remarks to the Author):

The authors are to be commended for thoroughly responding to previously indicated,including generating new data and effectively editing the text to clarify previously unclear comments and conclusions.

Reviewer #3 (Remarks to the Author):

The authors have addressed most of the previous concerns. The manuscript is significantly improved. However, the following remaining points should be addressed before acceptance for publication.

For the previous point #3, the authors responses are reasonable, although not directly addressed the concern. The authors should include a discussion on this point along the lines of their responses in the revised manuscript.

Similarly, the previous point #7 was not addressed directly due to technical issues and pandemic related delays. While the use of cell lines with different levels of Ambra1 is helpful, they do not alleviate the previous concern. This caveat of the study should be acknowledged in the revised text.

Point-by-Point Rebuttal Letter

<u>Reviewer #1</u>:

> ***Authors have addressed most of the points raised by the reviewers and the revised manuscript is notably improved.***

We are deeply grateful to the Reviewer for the comments and suggestions that have arisen during the revision process and, at the same time, honored that the efforts made to improve the quality of our data and of our manuscript have been appreciated.

<u>Reviewer #2</u>:

> ***The authors are to be commended for thoroughly responding to previously indicated, including generating new data and effectively editing the text to clarify previously unclear comments and conclusions.***

We are profoundly thankful to the Reviewer for the commendation as well as for the recommendations suggested during the revision phase of our manuscript and pleased for the appreciation of the new data generated and text editing.

<u>Reviewer #3</u>:

> ***The authors have addressed most of the previous concerns. The manuscript is significantly improved. However, the following remaining points should be addressed before acceptance for publication.***

We really appreciate the Reviewer for the comments and concerns raised during the revision phase of our manuscript. We also apologize for the missing information and, as requested, we have now addressed the new points in the revised version of the manuscript, as described hereinafter.

1) ***For the previous point #3, the authors responses are reasonable, although not directly addressed the concern. The authors should include a discussion on this point along the lines of their responses in the revised manuscript.***

We thank the Reviewer for this concern and have now included this point in the discussion of the revised manuscript, as follows: «Of note, though these changes could be largely related to the stromal composition of the tumor (*e.g.*, fibroblasts), the evidence that increased expression of EMT markers occurs both in human melanoma cells silenced for AMBRA1 and in *Ambra1* KO tumor-derived primary cells argues for a contribution of melanoma cells.».

2) ***Similarly, the previous point #7 was not addressed directly due to technical issues and pandemic related delays. While the use of cell lines with different levels of Ambra1 is helpful, they do not alleviate the previous concern. This caveat of the study should be acknowledged in the revised text.***

We thank the Reviewer and apologize for missing information. In the revision of the manuscript, we have now included this statement, as follows: «On the other hand, we demonstrated that the pharmacological inhibition of FAK1 signaling was sufficient to revert the growth of *Ambra1*-deficient *Braf/Pten*-driven melanomas *in vivo*. Moreover, a reduced invasiveness as well as an increased sensitivity to *FAKi* was observed in null or low-expressing AMBRA1 melanoma cells treated with *FAKi in vitro*. Although this evidence should be validated *in vivo* through FAK1 genetic ablation in order to circumvent possible off-target effects of pharmacological inhibition, concomitant silencing of FAK1 and AMBRA1 *in vitro* confirmed the reduced propensity of melanoma cells to migrate and invade.».
